# Switchable protection and exposure of a sensitive squaraine dye within a redox active rotaxane

**DOI:** 10.1038/s42004-024-01312-1

**Published:** 2024-10-04

**Authors:** Janos Wasternack, Hendrik V. Schröder, J. Felix Witte, Mihkel Ilisson, Henrik Hupatz, Julian F. Hille, Marius Gaedke, Arto M. Valkonen, Sebastian Sobottka, Alexander Krappe, Mario Schubert, Beate Paulus, Kari Rissanen, Biprajit Sarkar, Siegfried Eigler, Ute Resch-Genger, Christoph A. Schalley

**Affiliations:** 1 Institut für Chemie und Biochemie, Freie Universität Berlin, Arnimallee 20, 14195 Berlin, Germany; 2 Institut für Chemie und Biochemie, Freie Universität Berlin, Arnimallee 22, 14195 Berlin, Germany; 3 University of Jyvaskyla, Department of Chemistry, Survontie 9 B, 40014 Jyväskylä, Finland; 4 Institut für Chemie und Biochemie, Freie Universität Berlin, Fabeckstr. 34-36, 14195 Berlin, Germany; 5 Institut für Chemie und Biochemie, Freie Universität Berlin, Altensteinstraße 23A, 14195 Berlin, Germany; 6 Institut für Chemie und Biochemie, Freie Universität Berlin, Takustraße 3, 14195 Berlin, Germany; 7 Institut für Anorganische Chemie, Universität Stuttgart, Pfaffenwaldring 55, 70569 Stuttgart, Germany; 8 Bundesanstalt für Materialforschung und -prüfung (BAM), Biophotonics, Richard Willstätter Straße 11, 12489 Berlin, Germany

**Keywords:** Interlocked molecules, Molecular machines and motors

## Abstract

In nature, molecular environments in proteins can sterically protect and stabilize reactive species such as organic radicals through non-covalent interactions. Here, we report a near-infrared fluorescent rotaxane in which the stabilization of a chemically labile squaraine fluorophore by the coordination of a tetralactam macrocycle can be controlled chemically and electrochemically. The rotaxane can be switched between two co-conformations in which the wheel either stabilizes or exposes the fluorophore. Coordination by the wheel affects the squaraine’s stability across four redox states and renders the radical anion significantly more stable—by a factor of 6.7—than without protection by a mechanically bonded wheel. Furthermore, the fluorescence properties can be tuned by the redox reactions in a stepwise manner. Mechanically interlocked molecules provide an excellent scaffold to stabilize and selectively expose reactive species in a co-conformational switching process controlled by external stimuli.

## Abstract (web summary)

Incorporating sensitive chromophores into interlocked systems that are mechanically responsive to external stimuli is attractive for tuning of the dye’s optical properties and stability. Here, the authors report the mechanically controlled protection and deprotection of a squaraine dye within a [2]rotaxane, governed by macrocycle shielding/de-shielding upon chloride addition or oxidation.

## Introduction

From RNA enclosed in capsid shells^1,2^ to reactive molecular centers and active sites^3^ buried deep within shielding proteins, confinement in protective environments is a fundamental strategy in nature to mitigate undesired side reactions of labile species^4,5^. In specific molecular environments within proteins, for instance, reactive molecules such as organic chromophores, radicals, or ions are not only sterically shielded, but are also stabilized through non-covalent interactions. Prime examples are β-barrels hosting labile chromophores in fluorescent proteins^6^ or organic radical ions stabilized through hydrogen bonding in flavoenzymes^7^ and photosynthetic reaction centers^8^.

**Fig. 1:**
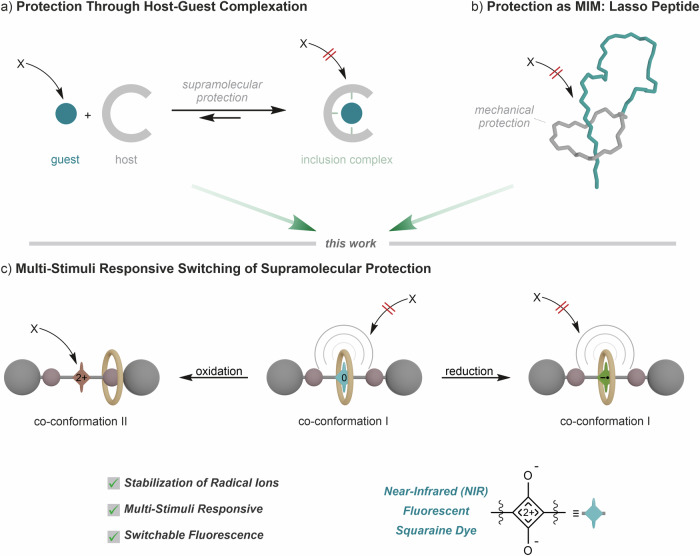
Different protecting strategies. **a** Encapsulation of a labile guest by a host molecule results in an inclusion complex in which the guest is sterically shielded from unwanted side reactions with a compound X. **b** Lasso peptide microcin J25^27^ as example of drastically increased physicochemical stability by mechanical bonding. **c** Electrochemical switching of protection by a macrocycle encircling a labile squaraine dye in a [2]rotaxane.

It remains a formidable challenge for synthetic chemists to stabilize reactive species (e.g., organic radicals) without interfering with the molecules’ functions to enable applications in biomedical imaging^9^, sensing^10^, or proximity labeling^11^. In contrast to classical covalent protecting group strategies, encapsulation by a receptor offers a supramolecular approach for the stabilization of reactive guests by non-covalent interactions (Fig. 1a). A host molecule, such as a macrocycle, confines a reactive guest and acts as a dynamically bound ‘supramolecular protecting group’. The protection strength is determined by energetic stabilization upon binding inside the host and steric shielding. The abundance of free, unprotected guest is significantly decreased upon binding inside a confined space^12–16^.

An alternative approach to increase stability involves the incorporation of reactive sites into mechanically interlocked molecules (MIMs) as seen for synthetic^17–21^ and natural^22–26^ examples like the lasso peptide microcin J25^27^ (Fig. 1b). An advantage compared to a non-interlocked complex is that the mechanical bond prevents dissociation into subcomponents and thus conformationally locks the stabilizing structure. For example, some mechanically interlocked peptides display mechanical bonds with a low degree of conformational freedom, equipping them with an unusually high physicochemical stability compared to their non-interlocked counterparts^22,27^. A synthetic example was reported by Smith et al., who applied protection within MIMs for sensitive squaraine dyes. Squaraine (Sq) dyes are important near infrared (NIR) fluorescent dyes with broad application in bioimaging^28–31^. NIR dyes offer low absorption interference by biological tissues, deeper tissue penetration, reduced scattering, and among them, Sq are of particular interest due to their highly intense NIR absorption and emission bands^31–33^. However, Sq suffer from their vulnerability to nucleophilic attack at the dye’s electrophilic C_4_O_2_ core, resulting in color fading^28^. Their enclosure by Hunter-Vögtle type tetralactam macrocycles encircling the Sq core through NH ∙ ∙ ∙ O hydrogen bonds is a useful means of protection against nucleophilic attack^28^.

A particularly interesting aspect of MIMs is that co-conformational flexibility can be intentionally designed into a molecular structure, enabling controlled large-amplitude motions of their subcomponents. This property has made MIMs preferred scaffolds for the construction of artificial molecular switches and machines^34–38^ mimicking dynamic conformational changes found in biological nanomachines. In Smith’s Sq rotaxanes, addition of chloride salts leads to the displacement of the macrocycle from the Sq station, thus switching off the protection^28–30^.

To make such switchable protection multi-stimuli responsive is the aim of this work. Besides switching by chloride addition and removal, we demonstrate that electrochemical switching can also be realized in this Sq rotaxane. We show that the energetic stabilization and steric shielding of the reactive Sq fluorophore within a conformationally dynamic MIM can be altered by electrochemical and chemical stimuli. These external stimuli either increase the stabilization by the wheel or induce a co-conformational change, resulting in the controllable exposure of this reactive site (Fig. 1c). Furthermore, the rotaxane exists in four oxidation states, which can be utilized for tuning of the optoelectronic properties of those dyes.

## Results and discussion

### Preparation and structure of Rot

**Fig. 2:**
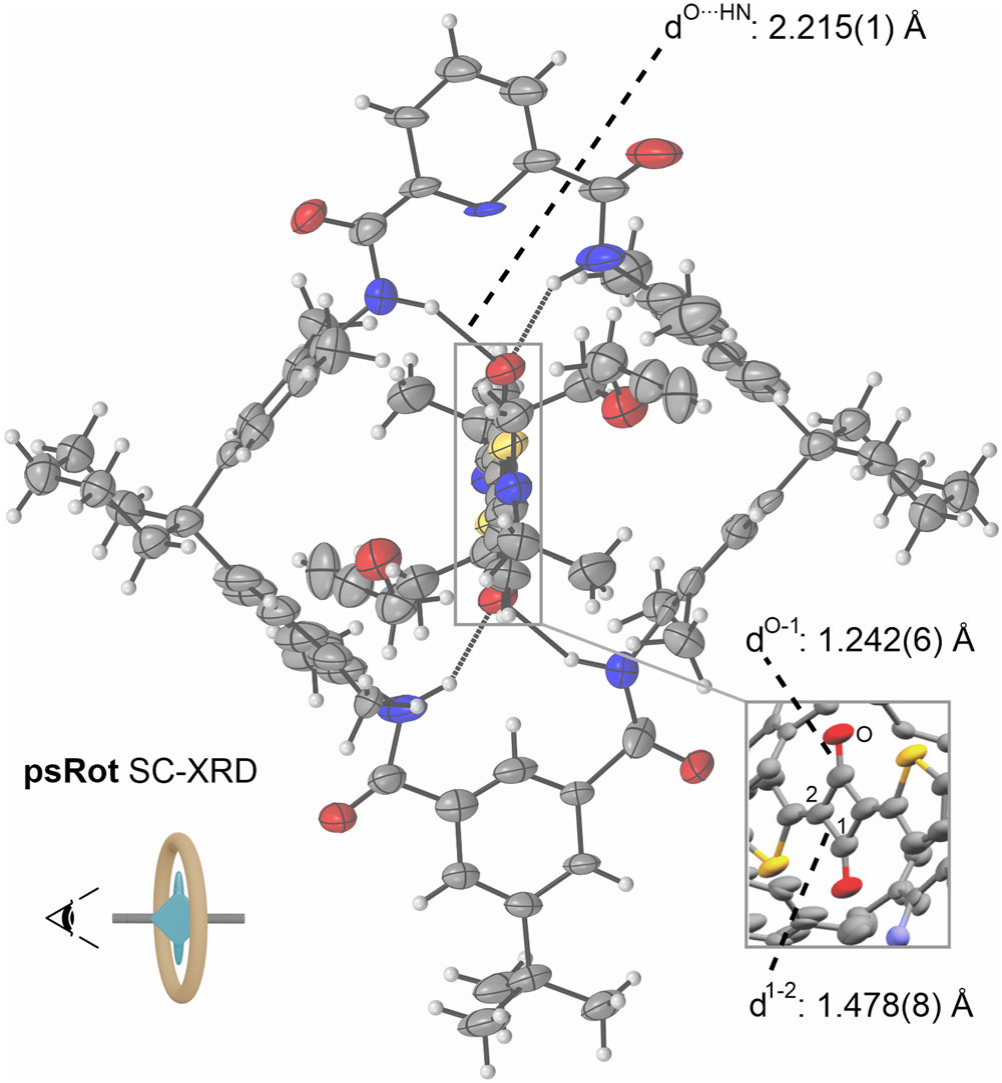
Single Crystal XRD structure of psRot with selection of bond lengths (thermal displacement parameters at 50% probability level). The perspective is indicated by the point of view icon next to the schematic depiction. The macrocycle is positionally disordered in the crystal and the pyridine dicarboxylic amide and isophthalic diamide can replace each other in their positions.

**Fig. 3:**
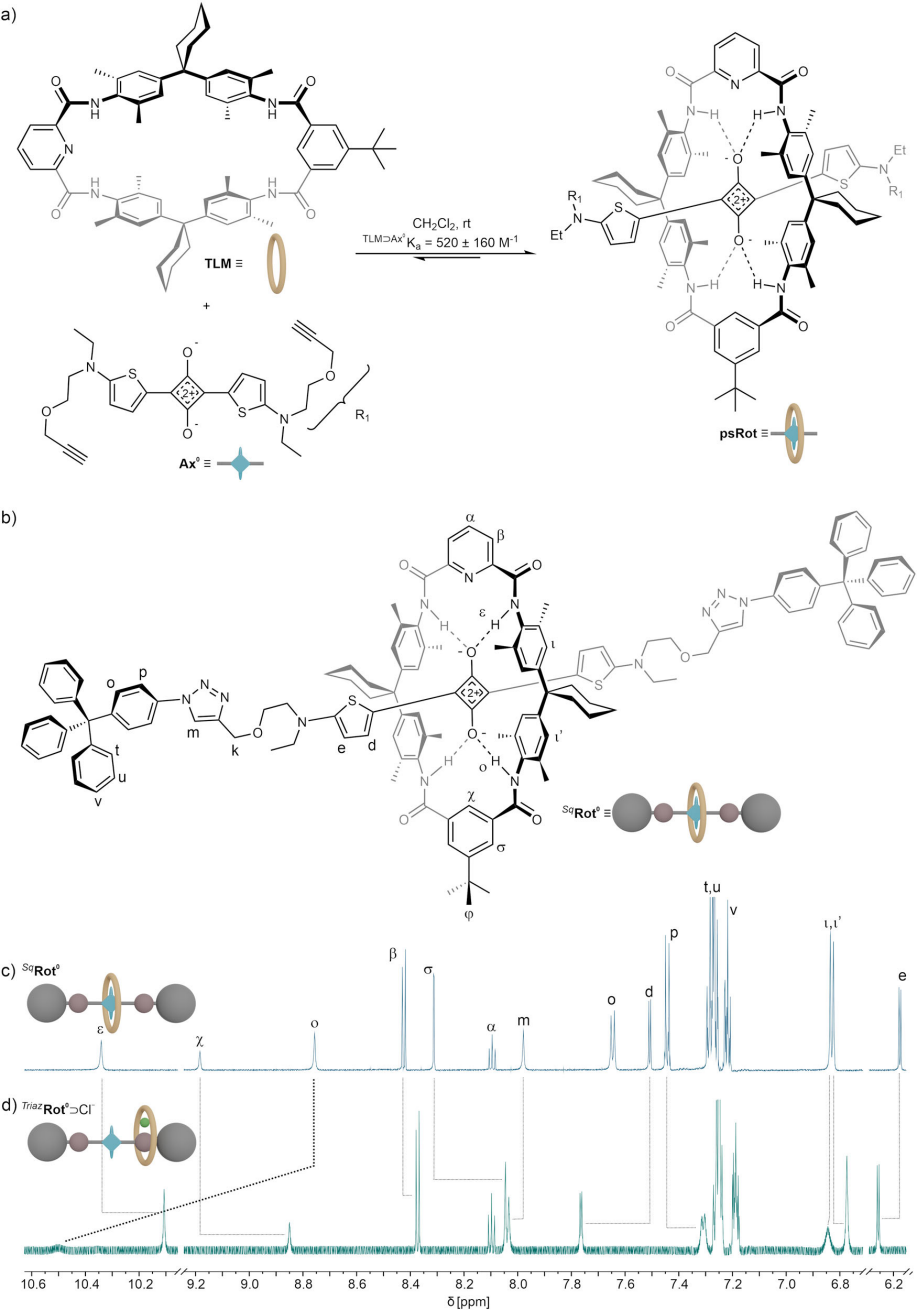
Structure of rotaxane Rot and chloride-induced shuttling. **a** Equilibrium between **Ax**, **TLM** and **psRot** & schematic depictions of these species. **b** Molecular structure of rotaxane **Rot** with schematic depiction. **c** Partial ^1^H NMR spectrum of ^Sq^**Rot**^**0**^. **d** Partial ^1^H NMR spectrum of ^Triaz^**Rot**^**0**^⊃Cl^−^ after addition of 59 equiv. n-Bu_4_NCl.

**Fig. 4:**
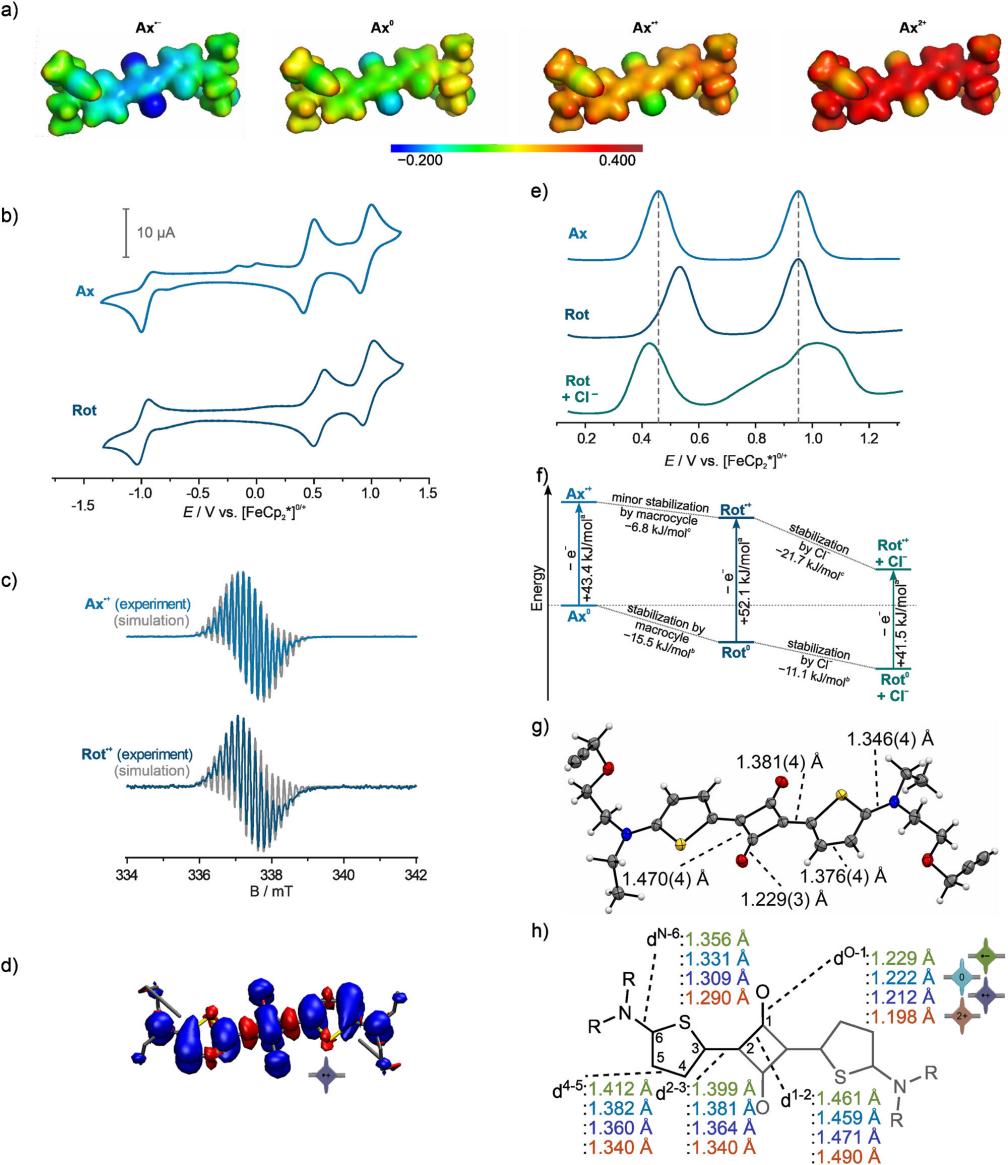
Opto-electrochemical investigation of the electronic and co-conformational properties of Ax and Rot. **a** Electrostatic potential surface maps of **Ax** in four different oxidation states. **b** Cyclic voltammograms (100 mV/s) of **Ax** and **Rot**. The second scan cycle is shown. The additional peaks at approximately −0.1 V in the second scan cycle, the i_pa_ / i_pc_ ratio, and the non-linear behavior of i_pa_ at different scan rates (inset) indicate an EC_i_ mechanism for the one-electron reduction of **Ax**. **c** EPR spectra of **Ax**^•+^ and **Rot**^•+^ (**d**) Spin density plots the radical species **Ax**^•+^, isovalue: 0.02 a.u. **e** Differential pulse voltammograms (10 mV/s scan rate, 25 mV modulation amplitude, 50 ms modulation time, 5 mV step potential, 0.5 s interval time) of **Ax,** **Rot**, and **Rot** after addition of excess n-Bu_4_NCl. The second oxidation reaction of the rotaxane in presence of chloride appears irreversible in both the DPV and CV. **f** Energy diagram, illustrating the stabilizing and destabilizing effects of the macrocycle and the presence of chloride on the first oxidation potentials of the Sq in **Ax** & **Rot**. Energies were calculated from (^a^) redox potentials: Δ*G* = *n* ∙ *F* ∙ *E*, (^b^) binding constants: Δ*G* = −*R* ∙ *T* ∙ ln(K_a_) and (^c^) according to Hess’ law Δ*G*^c^ = ΣΔ*G*^a&b^. **g** The SCXRD-structure of **Ax** (thermal displace parameters at 50% probability level). **h** Annotation of discussed bond lengths (from DFT calculations) and rotational barriers within the bis(aminothienyl)squaraine core.

[2]Rotaxane **Rot** comprises a Hunter-Vögtle type tetralactam macrocycle **TLM** and a threaded and mechanically interlocked bis(aminothienyl)squaraine axle. This macrocycle was chosen because the pyridyl group facilitates binding and preorganisation during the synthesis of **Rot** and also improves solubility of the macrocycle^39^. Wheel **TLM** and axle **Ax** form a threaded complex, pseudo[2]rotaxane **psRot** in dichloromethane with an association constant of *K*_a_ = 520 ± 160 M^-1^ at 293 K as determined by ^1^H NMR titration (SI section S2 Fig. S17). Slow evaporation of dichloromethane solutions of **Ax** and of **psRot** yielded single crystals suitable for XRD analysis. The 1:1 complex stoichiometry was confirmed by solid-state XRD structures of the **psRot** and **Ax** (Figs. 2 and 4g). The structure of **psRot** is similar to reported Sq tetralactam rotaxanes (SI section S4 Fig. S25)^28,30,40^. The Sq bound by four NH ∙ ∙ ∙ O hydrogen bonds (2.21 Å) and the bond lengths within the neutral Sq within **psRot** are very close to the ones in free **Ax**. **Rot** was obtained in 32% yield from the solution of **psRot** in an end-capping approach *via* copper-catalyzed azide-alkyne Huisgen cycloaddition of a sterically demanding trityl stopper (Figs. 3b and 8). The most stable co-conformation ^Sq^**Rot**^**0**^ involves the wheel encircling the squaraine core by forming NH ∙∙∙ O hydrogen bonds.

### Chloride switching of neutral Rot

Addition of chloride salts to a dichloromethane solution of **Rot** results in a significantly increased fluorescence intensity, likely caused by shuttling of the wheel to the vicinity of the triazol group, thereby exposing the squaraine core (Figs. S21–S23). This shuttling behavior has been observed in similar tetralactam rotaxanes^28,40^. The fluorescence quantum yields as well as the maxima of absorption and emission of the fluorescent states of **Ax** and **Rot** were measured (SI section S3, Table S1). **Ax**^**0**^ displays fluorescence with an emission maximum of λ_max_(**Ax**^**0**^) = 678 nm and an absolutely measured fluorescence quantum yield of Φ(**Ax**^**0**^) = 6.7% and **Rot**^**0**^ displays fluorescence with an emission maximum of λ_max_(**Rot**^**0**^) = 683 nm and a quantum yield of Φ(**Rot**^**0**^) = 4.0%. The lower quantum yield of **Rot**^**0**^ could be attributed to lower absorption of the shielded Sq or partial absorption of the emitted light by the wheel. Addition of 50 equiv. Bu_4_NCl to a solution of **Ax**^**0**^ leads to a decreased quantum yield of Φ(**Ax**^**0**^⊃Cl^−^) = 5.9%, while under the same conditions the quantum yield of **Rot**^**0**^ increases to Φ(**Rot**^**0**^⊃Cl^−^) = 4.2%. The increased fluorescence intensity in ^Triaz^**Rot**^**0**^⊃Cl^−^ thus likely results from increased absorbance and reduced quenching by the wheel of the deshielded squaraine. The observed properties and the chloride induced translocation of the wheel are in good alignment with similar systems^28,40^.

The chloride affinity of **Rot**^**0**^ was determined by ^1^H NMR (*K*_a_ = 84 ± 12 M^-1^) and fluorescence titrations (*K*_a_ = 90 ± 20 M^-1^) using *n*-Bu_4_NCl (Figs. S19–S22). Figure 3 shows the ^1^H NMR spectra of **Rot** before and after addition of 59 equiv. *n*-Bu_4_NCl. Binding of chloride induces the co-conformational change to ^Triaz^**Rot**^**0**^⊃Cl^−^ in which the wheel **TLM** binds a chloride ion and the triazole station, as indicated by characteristic shifts of the NH signals ε: ∆δ(ε) = 0.2 ppm and ο: ∆δ(ο) = −1.7 ppm, and the aromatic signals χ: ∆δ(χ) = 0.3 ppm and σ: ∆δ(σ) = 0.2 ppm. A binding mode of chloride in similar tetralactam - triazol rotaxanes through CH ∙∙∙Cl^−^∙∙∙HN hydrogen bonds between the triazole’s CH group and the NH groups of the tetralactam has been reported^41,42^.

Quantum chemical investigation of ^Triaz^**Rot**^**0**^⊃Cl^−^ by MD simulations using CREST, indicate hydrogen bonding between the amide NH protons of the isophthalamide and chloride, while the pyridyl bis amide participates in hydrogen bonding towards the triazol group. Weak hydrogen bonds between the triazol proton m and chloride are indicated by the comparatively low shift of m: ∆δ(m) = 0.1 ppm. This is in line with the calculated binding motif in which the triazol is tilted away from the bound chloride anion (SI section S9 Fig. S42). The presence of only one set of broadened signals suggests that translational exchange of the wheel between the two triazoles is faster than the NMR time scale. Precipitation of the chloride by addition of Na[B(Ph-3,5-(CF_3_)_2_)_4_] leads to back shuttling of the wheel onto the Sq. Reversibility of the shuttling was again confirmed by fluorescence spectroscopy (Fig. S23).

### Redox switching of Rot

We hypothesized that a similar switching between co-conformations might be realized by electrochemistry. Leigh and co-workers reported an electrochemically switchable hydrogen-bonded molecular shuttle^43^. We recently developed a series of tetrathiafulvalene rotaxanes, which are electrochemically switchable between different co-conformations^44–53^. (Quasi)reversible redox reactions have been reported for some Sq derivatives^54^.

Cyclic voltammetry in 1,2-dichloroethane (Fig. 4b and Table S2) reveals that **Ax**^**0**^ undergoes an irreversible one-electron reduction to the radical anion **Ax**^**●−**^ (*E*_pc_^-1^ = − 0.99 V against the decamethylferrocene/decamethylferrocenium couple) as well as two reversible one-electron oxidations, to the radical cation **Ax**^**●+**^ (*E*_1/2_^1^ = 0.45 V) and dication **Ax**^**2+**^ (*E*_1/2_^2^ = 0.95 V). These three redox reactions are most likely localized on the thiophene-squaraine π-system^54^.

Scan rate-dependent plots of the anodic peak potential (SI section S5 Figs. S26 and S29) indicate that the reduction of the axle (**Ax**^**0**^ → **Ax**^**●−**^) is followed by an irreversible chemical reaction step in a so-called EC_i_ mechanism. For Sq^●−^, Quantum chemical calculations at various levels of density functional theory (DFT) (SI section S9) indicate elongated O-C^1^- bonds and an increase of electron density on the oxygen atoms (Fig. 4a and h, Table S4). In **Rot**^**●−**^, both effects result in stabilization of the NH ∙ ∙ ∙ O hydrogen bonds between Sq^**●−**^ and wheel by 42 kJ/mol (Table S7). In addition to the electronic stabilization of the wheel–Sq^**●−**^ interaction, the steric shielding of Sq^**●−**^ by **TLM** contributes to an observed increase in lifetime. In contrast to the (quasi)irreversible reduction of **Ax**^**0**^, the reduction of **Rot**^**0**^ is reversible on the CV timescale (scan rate of 100 mV/s), further supporting the significant stabilization of the radical anion Sq^●−^ by rotaxanation. The lifetime of the radical anion **Rot**^**●−**^ was investigated by CV-based digital simulations (SI section S5 Fig. S29). Fitting the experimental voltammograms at different scan rates according to an EC_i_ mechanism yields an average radical lifetime of 1.7 s (**Ax**^**●−**^) and 10.8 s (**Rot**^**●−**^). However, the anodic peak (*E*_pa_) of **Rot** was overlayed by incipient solvent/electrolyte decomposition. Thus, the life-time ratio *τ*_**Rot**_/*τ*_**Ax**_ > 6.7 ± 2.8 is considered to be a lower boundary of the stabilization effect caused by rotaxanation. Organic radicals are typically highly reactive species with short lifetimes^55^. They were categorised by Ingold according to their lifetimes as transient radicals (lifetimes < 1.44 ms) and persistent radicals (lifetimes > 1.44 ms)^56,57^. For example the benzyl radical is transient with a lifetime below 1 ms^57,58^. The drastic impact of thermodynamic and steric factors on radical stability are illustrated by the existence of bench stable radicals like TEMPO. Supramolecular encapsulation is a common means of stabilisation of transient and persistent radicals^4,43,55,59–64^. A prominent example being the drastic stabilisation of a persistent tetrazine radical anion with a lifetime of 2 h through encapsulation by two cyanostar macrocycles leading to a complex with a 360-fold lifetime of 30 d^64^. Our case thus represents the stabilisation of a more reactive persistent radical.

In contrast to the strengthening of the wheel–axle interaction in **Rot** by the reduction, oxidation reduces the electron density of Sq as indicated by calculated electrostatic potential surface maps (ESPs), resulting in a weakened wheel–axle interaction in **Rot**^**●+**^ and **Rot**^**2+**^ (Fig. 4a and d and S43, Tables S4 and S7).

**Fig. 5:**
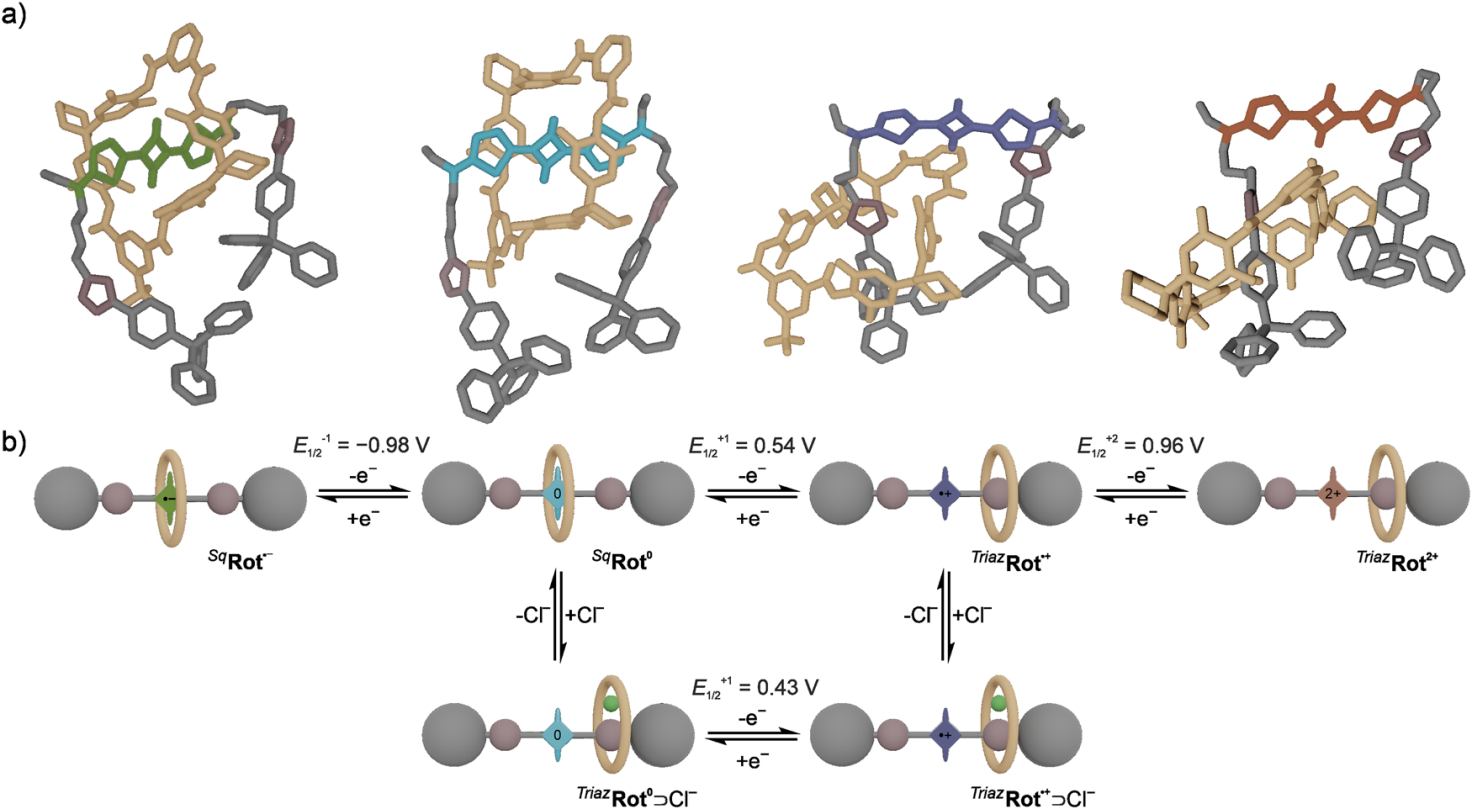
Structural investigation of the most stable co-conformational space of Rot. **a** Calculated most stable co-conformations of **Rot** in four different oxidation states with schematic depictions of each state. **b** Co-conformational electrochemical and chemical equilibrium between six states of **Rot**. Only the most stable co-conformations of each state are depicted.

The one-electron oxidation potential of **Rot**^**0**^ is indeed anodically shifted by Δ*E*_1/2_^1^ = +90 mV compared to free **Ax**^**0**^, indicating that the Sq–wheel interaction energetically hampers the first oxidation (Fig. 4e, f). We investigated the co-conformational equilibrium between ^**Sq**^**Rot** and ^**Triaz**^**Rot** in four oxidation states by exploring the co-conformational landscapes using the CREST code^65,66^ by Grimme and subsequent evaluation of energetic stabilities employing various density functional approximations (Fig. 5 and Table S7). Whereas the neutral Sq station is energetically favored over the triazole station by 29 kJ/mol, the first oxidation renders the triazole station slightly more favorable than the Sq^●+^ station by 3.5 kJ/mol. Hence, one-electron oxidation results in molecular shuttling (^**Sq**^**Rot**^**0**^ → ^**Triaz**^**Rot**^●+^) and a co-conformational distribution in which the wheel preferably adopts a distal position to Sq (Fig. 5).

Gradual oxidation of **Rot**^**0**^ in an electron paramagnetic resonance (EPR) spectroelectrochemical experiment (dichloromethane, 298 K) gave an isotropic signal at *g* = 2.003 (Fig. 4c) confirming its transition into a stable radical cation Sq^●+^. A low temperature spectrum at 223 K reveals the underlying hyperfine structure of the signal partially. The best fit, which is in excellent agreement with the experimental spectrum, was obtained with a simulation using a *g*-value of 2.004 and hyperfine coupling to two equivalent ^14^N nuclei and three sets of equivalent ^1^H nuclei totaling 14 hydrogen nuclei (four ^1^H thienyl, two ^14^N amine, eight ^1^H N-C^1^*H*_2_) (Table S3). Supported by a spin density plot derived from DFT calculations, the experimental data indicate that the radical in Sq^●+^ is delocalized over the thiophene-Sq system (Fig. 4d and Fig. S43). The low temperature EPR spectrum of singly oxidized **Rot**^**●+**^ shows very similar spectral features indicating virtually no electronic effect of the wheel on the electronic coupling of the axle-centered radical cation. Similar findings have been observed for other hydrogen-bonded radicals^4^ and could result from weakened wheel–Sq interactions in **Rot**^●+^ and/or subsequent co-conformational changes as indicated by the electrochemical experiments.

The second one-electron oxidation (**Rot**^●+^/**Rot**^**2+**^) occurs at a similar potential compared to that of the free **Ax**^●+^ (Δ*E*_1/2_^2^ = +10 mV), indicating that hydrogen bonding between Sq and the wheel in **Rot**^●+^ is significantly weakened compared to the neutral state (Fig. 4a, e, and f and 5). Consequently, the second oxidation of the deshielded Sq energetically resembles the free axle more than the first oxidation, as no further conformational equilibria contribute to the stabilization or destabilization of the system. Further evidence was derived from differential pulse voltammetry utilizing **Rot**^**0**^, both in the absence and presence of one equivalent *n*-Bu_4_NCl (Fig. 4e). As the chloride-binding wheel in complex ^**Triaz**^**Rot**^**0**^⊃Cl^−^ adopts a distal position to Sq, the first oxidation reaction displays a potential (*E*_1/2_^1^ = 0.43 V) similar to free **Ax**^**0**^. This illustrates that the increased oxidation potential in **Rot**^**0**^ is indicative of the wheels position: The first oxidation potential of **Rot**^**0**^ is higher than that of free **Ax**^**0**^, because the stabilization energy in **Rot**^**0**^ arising from rotaxanation and the intramolecular wheel–Sq hydrogen bonds, needs to be compensated upon oxidation. As the chloride-addition already leads to a co-conformation ^**Triaz**^**Rot**^**0**^⊃Cl^−^ in which the Sq is deshielded, the binding energy of the macrocycle is compensated for by the interaction between the wheel, triazole and chloride. The subsequent oxidation of the deshielded Sq thus requires a similar potential as the free axle. Further, the slightly lower oxidation potential of **Rot**^**0**^ in the presence of chloride suggests a stabilization of the oxidized **Rot**^●+^⊃Cl^−^ by the strongly coordinating chloride anion^67^. Further oxidation to **Rot**^**2+**^⊃Cl^−^ is irreversible, likely caused by a nucleophilic attack at the now sterically deprotected Sq core^28^.

Theoretical calculations show doubly oxidized **Rot**^**2+**^ to have a pronounced energetic bias towards the triazole station of 18 kJ/mol (Fig. 5 and Table S7). The reduced electron density and thus, significantly lower H-bond accepting capability of Sq^2+^ is illustrated by electrostatic potential surface maps of the stoppered axle as found within **Rot**. The negative charge accumulation at Sq within **Rot**^**●−**^ and **Rot**^**0**^ and conversely, the lack of negative charge at Sq in **Rot**^**●**+^ and **Rot**^**2+**^ are depicted in Fig. S43. Thus, the H-bond accepting ability of Sq decreases drastically from Sq^●−^ to Sq^2+^, while more negative charge is retained at the triazole groups in the oxidized states. In accordance with the electrochemical and quantum chemical data the interaction between Sq and the wheel are already weak enough in **Rot**^**●**+^ that the wheel adopts a position close to the triazole motif. Quantum chemical investigation of this binding motif suggests H-bonding between two NH protons of the wheel and the electron rich N atoms of the triazol unit (Fig. S42).

To investigate the oxidation induced shuttling (^**Sq**^**Rot**^**0**^ **→** ^**Triaz**^**Rot**^**2+**^) by 1D and 2D NMR experiments, we developed a reliable method to obtain chemically stable solutions of **Ax**^**2+**^ and **Rot**^**2+**^. Initial attempts using NOPF_6_ as oxidant in dichloromethane-*d*_2_ resulted in partial precipitation. In acetonitrile, the resulting dye solutions decompose within minutes. The solubility and lifetime in dichloromethane-*d*_2_ of the Sq^2+^ species could be drastically increased (time scale of days) by addition of the weakly coordinating anion [Al(O-C(CF_3_)_3_)_4_]^−^ ([pf]^−^) as Li[pf] during the oxidation with NOPF_6_. **Rot**^**2+**^ did not display a stabilizing effect compared to the dicationic **Ax**^**2+**^, as expected for the Sq being exposed in this state (see below for stability study). Oxidation reactions for both, **Ax** and **Rot**, are reversible, as demonstrated by using tin powder as reductant in an UV-Vis titration (SI section S7 Fig. S34).

The ^1^H NMR spectrum of **Ax**^**2+**^ displays multiple sets of signals which can be attributed to rotational isomers of **Ax**^**2+**^ (Supporting Information Section S1, S6 and S7). Quantum chemical calculations reveal a four-fold increase of rotational barriers in **Ax**^**2+**^ compared to **Ax**^**0**^ (SI section S6 Table S4 and section S9 Table S5). At room temperature these rotations are free in **Ax**^**0**^ and hindered in **Ax**^**2+**^ leading to the observed spectral behavior. The much lower rotational barriers of **Ax**^**0**^ and **Rot**^**0**^ cause decoalescence of most signals only below −30 °C (SI section S8). Based on the calculated barriers for **Ax**^**0**^, the rotation around the bonds between the Sq and the thiophene units should be frozen first, due to their higher barrier.

NOE cross peaks between proton signals of one of triazoles and methyl groups of the macrocycle indicate the wheel to be located at the triazole in **Rot**^**2+**^ (Figs. S6–S10). Multiple sets of signals and extensive peak broadening in the 1D and 2D NMR spectra reveal a slow shuttling motion of the wheel between the two triazoles. Additionally, the non-bound side of the axle likely displays rotational isomerism like **Ax**^**2+**^.

**Fig. 6:**
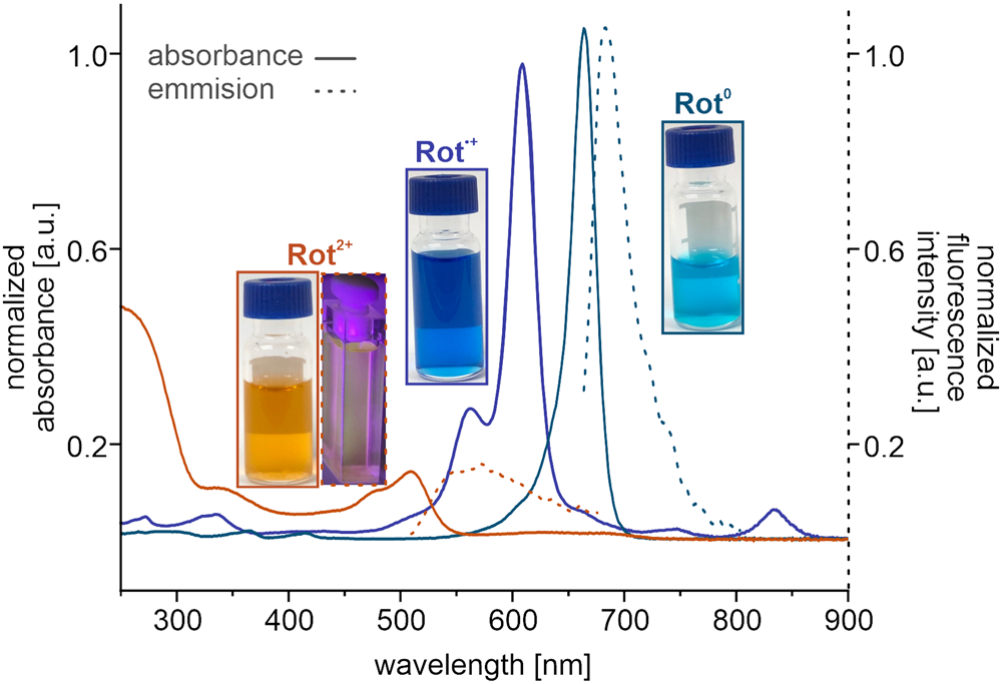
UV-Vis (solid) spectra of Rot in three oxidation states and fluorescence (dashed) spectra of Rot^0^ and Rot^2+^. The inset photographs on each curve depict a vial containing dichloromethane solution of each species, the counterion is [pf] ^–^.

The stability of both oxidized states, **Rot**^**●+**^ and **Rot**^**2+**^, on a laboratory time scale allowed us to study their optical properties (Fig. 6 and S34 and SI section S3). The first oxidation of **Rot**^**0**^ is accompanied by a hypsochromic shift of the most intense absorbance band, **Rot**^**0**^ (λ_max_ = 665 nm) to **Rot**^**●+**^ (λ_max_ = 601 nm). The strongest band is further shifted by the second oxidation from **Rot**^**●+**^ (λ_max_ = 601 nm) to **Rot**^**2+**^ (λ_max_ = 509 nm). The emission band of **Rot**^**0**^ (λ_max_ = 683 nm, Φ(**Rot**^**0**^) = 4.0%) is close to the bands of similar reported bis-(aminothienyl)squaraine rotaxanes^68^. Due to the disproportionation equilibrium from **Rot**^**•+**^ into **Rot**^**0**^ and **Rot**^**2+**^, strong emission bands of the neutral and doubly charged species limit the detection of potential fluorescence of the radical cation. Dicationic **Rot**^**2+**^ (λ_max_ = 570 nm, Φ(**Rot**^**2+**^) = 0.6%) shows weak fluorescence, a photograph of the luminescence is depicted as inset in Fig. 6. In line with the ^**Triaz**^**Rot**^**2+**^ co-conformation, the quantum yield is almost equal to that of **Ax**^**2+**^ (λ_max_ = 548 nm, Φ(**Ax**^**2+**^) = 0.5%) (SI section S3 Fig. S24 and Table S1). The change of absorption properties was supported by time-dependent DFT calculations, suggesting that charge-transfer effects upon excitation play a role in **Ax**^**0**^ and **Ax**^**●+**^. The difference density of the bright state displays a charge transfer from Sq to the neighboring thiophene moieties (Table S6).

To further elucidate the protection and deprotection in the neutral and +2 oxidation states, bleaching of the squaraine core in **Ax**^**0**^, **Rot**^**0**^, **Ax**^**2+**^ and **Rot**^**2+**^ by attack of nucleophiles was investigated using UV-Vis spectroscopy (SI section S10 Fig. 43). Tetramethylammonium hydroxide in dichloromethane proved effective in bleaching **Ax**^**2+**^ and **Rot**^**2+**^, while **Rot**^**0**^ was unaffected (Fig. S44b–d). While the half-life of **Ax**^**2+**^ (≈10 min) and **Rot**^**2+**^ (≈5 min) under these conditions is comparable, **Rot**^**0**^ was stable (Fig. S44a). Because the squaraine core in the neutral rotaxane is shielded by the macrocycle from nucleophilic attack, no significant bleaching occurs. In line with the suggested co conformation ^**Triaz**^**Rot**^**2+**^, the deshielded Sq^2+^ core bleaches similarly quickly as in the free **Ax**^**2+**^.

The shielding effect of the macrocycle has been demonstrated for similar systems^28^. As demonstrated above, low concentrations of hydroxide are not sufficient to bleach **Rot**^**0**^. At higher concentrations of OH^−^, the possibility of shuttling by binding of hydroxide in a similar motive as with chloride, cannot be excluded. Thus, for the comparison of the stability of **Ax**^**0**^ and **Rot**^**0**^, sterically demanding potassium ^*t*^butoxide was used (Fig. S44f, g). To increase the solubility of the nucleophile, 5% of ^*t*^BuOH were added to the solvent. In this mixture, free **Ax**^**0**^ was bleached (≈12 min), while **Rot**^**0**^ was affected only very little, indicating the expected protection of the squaraine core within the bound wheel (Fig. S44e).

## Summary and conclusion

In this proof-of-concept study, we show that a co-conformationally dynamic rotaxane can serve as scaffold to either stabilize or expose high-performing dye molecules used for bioimaging applications, in this case a near‐infrared squaraine dye. [2]Rotaxane **Rot** can be reversibly switched between four different redox states, resulting in two different co-conformations, in which the squaraine dye is either shielded (oxidation state 1 and 0) or exposed (oxidation state +1 and +2). Shielding results in an increase of the dye’s lifetime, whereas the exposed states display physicochemical and optoelectronic properties similar to the non-complexed dye. At the same time, the squaraine-centered redox reactions allow for a stepwise tuning of the emission between 570 and 683 nm of the fluorescent **Rot**^**0**^ and **Rot**^**2+**^ states. A potential application involves the transport of labile fluorophores—stabilized by mechanical bonding—into a location where the co-conformational equilibrium of the rotaxane is triggered leading to the exposure of the fluorophore or a different reactive species. Such a system could be useful for applications in bioimaging (e.g. for multiplexing), sensing, and proximity labeling.

## Methods

### General

NMR experiments were performed on JEOL ECX 400, JEOL ECP 500, Bruker AVANCE 500, JEOL ECZ 600, and Bruker AVANCE 700 instruments. Residual solvent signals were used as the internal standards. All shifts are reported in ppm and NMR multiplicities are abbreviated as s (singlet), d (doublet), t (triplet), m (multiplet) and br (broad).

High-resolution ESI mass spectra were recorded on an Agilent 6210 ESI-TOF mass spectrometer. HPLC grade solvents were used for sample preparation and the samples were introduced into the ion source with a flow rate of 2–4 µL/min. UV/Vis spectra were recorded on a Varian Cary 50 Bio spectrometer with a xenon lamp. Fluorescence spectra were recorded on Infinite® M Nano^+^ (Tecan Deutschland GmbH, Crailsheim, Germany) and PerkinElmer Fl 6500 spectrometers. Suprasil glass cuvettes with path-lengths of 1 cm were used. Photoluminescence quantum yields (Φ) were measured with an integrating sphere setup from Hamamatsu (Quantaurus-QY C11347-11). All measurements were performed at room temperature using 10 mm × 10 mm long neck quartz cuvettes.

All reagents and solvents were obtained from commercial sources and used without further purification. Lithium tetrakis(perfluoro-^*tert*^butoxy)aluminate was provided by Prof. Dr. Ingo Krossing and Malte Sellin (University of Freiburg, Germany). Dry solvents were purchased from Acros Organics. Deuterated solvents were purchased from Eurisotop and Deutero. Deuterated dichloromethane was dried by vigorous stirring with calcium hydride for 2 weeks at room temperature.

*N*-Ethyl-*N*-(2-(prop-2-yn-1-yloxy)ethyl)thiophen-2-amine^69^, 4-trityl-phenylazide^42^ and tetralactam macrocycle **TLM**^70^ were synthesized according to literature procedures. Thin-layer chromatography was performed on silica gel coated plates with fluorescent indicator F254 (Macherey-Nagel). For column chromatography, silica gel (0.04-0.063 mm, Macherey-Nagel) was used.

### Synthesis

Bis(aminothienyl)squaraine Axle **Ax**^68^

**Fig. 7:**
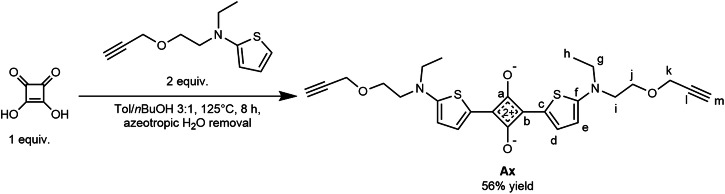
Adapted procedure for the preparation of squaraine **Ax**.^*55*^.

^**1**^**H NMR** (600 MHz, CD_2_Cl_2_): δ = 7.88 (d, *J* = 4.7 Hz, 2 H, **d**), 6.31 (d, *J* = 4.7 Hz, 2 H, **e**), 4.17 (d, *J* = 2.4 Hz, 4 H, **k**), 3.79 (t, *J* = 5.4 Hz, 4 H, **j**), 3.69 (t, *J* = 5.4 Hz, 4 H, **i**), 3.61 (q, *J* = 7.2 Hz, 4 H, **g**), 2.50 (t, *J* = 2.4 Hz, 2H, **m**), 1.30 (t, *J* = 7.2 Hz, 6 H, **h**) ppm.

^**13**^**C{**^**1**^**H} NMR** (151 MHz, CD_2_Cl_2_): δ = 178.2, 171.5, 170.0, 138.1, 115.6, 109.3, 79.6, 75.1, 67.4, 58.9, 54.2, 50.4, 12.2 ppm.

Bis(aminothienyl)squaraine axle **Ax** was prepared according to a modified literature procedure^68^ (Fig. 7): A mixture of toluene (750 mL) and *n*-butanol (250 mL) was azeotropically dried by refluxing on a Dean-Stark trap (oil bath 145 °C) for 2 h. The collected water was drained and the solvent mixture was cooled under N_2_-atmosphere. *N*-Ethyl-*N*-(2-(prop-2-yn-1-yloxy)ethyl)thiophen-2-amine (9.7 g, 47 mmol, 2.0 equiv.) and squaric acid (2.7 g, 24 mmol, 1.0 equiv.) were added. A color change from yellow to deep green to deep blue to black was observed. The reaction mixture was heated to reflux under N_2_ on the Dean-Stark trap (oil bath 145 °C) for 8 h. The solvents were removed under reduced pressure, and the metallic green residue was purified by column chromatography using dichloromethane-acetone (0% → 30% acetone) as eluent. Analytically pure **Ax** was obtained as a metallic green solid (6.50 g, 13 mmol, 56% yield), the analytical data are consistent with the reported values (Figs. S1, S2, and S16)^68^.

**HRMS (ESI):** m/z calculated for C_26_H_28_N_2_O_4_S_2_ [M]^●+^ 496.1501, found 496.1513.

Squaraine-TLM Rotaxane **Rot**

**Fig. 8:**
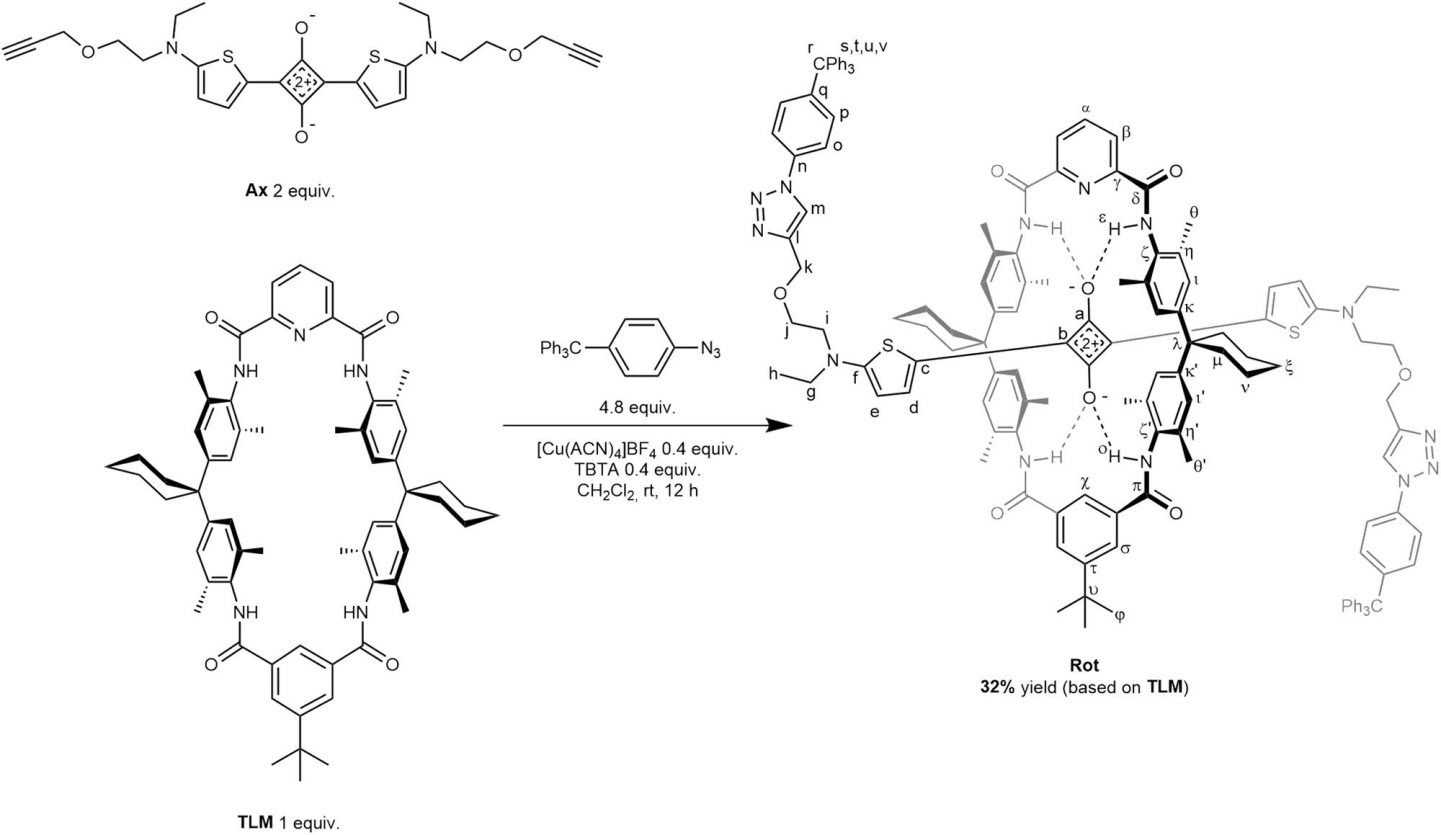
Capping-synthesis of **Rot** by Cu^II^ catalyzed azide alkyne Huisgen cycloaddition of trityl phenyl azide with the pseudo[2]rotaxane of **Ax** and **TLM** in dichloromethane. TBTA: tris((1-benzyl-4-triazolyl)methyl)amine.

Rotaxane **Rot** was prepared in the following manner (Fig. 8): A 25-ml dried Schlenk flask was charged with tetralactam macrocycle **TLM** (190 mg, 0.20 mmol, 1.0 equiv.) and **Ax** (200 mg, 0.40 mmol, 2.0 equiv.) under N_2_ counterflow. Dry dichloromethane (8 mL) was added, the flask was sealed, and the mixture was stirred for 1 h at room temperature. After that, 4-trityl-phenylazide (350 mg, 0.96 mmol, 4.8 equiv.), Cu(ACN)_4_BF_4_ (25 mg, 0.08 mmol, 0.4 equiv.) and tris((1-benzyl-4-triazolyl)methyl)amine (TBTA) (43 mg, 0.08 mmol, 0.4 equiv.) were added under N_2_ counterflow. The flask was sealed again and stirred overnight at room temperature. The mixture was purified by silica column chromatography using step gradient elution (0.8% → 2.6% methanol in dichloromethane). **Rot** eluted with 1.3% → 1.5% methanol content.

Analytically pure **Rot** was obtained as a deep blue crystalline solid (140 mg, 0.06 mmol, 32% based on **TLM**) (Fig. S11, 12, and 16).

^**1**^**H NMR** (600 MHz, CD_2_Cl_2_): δ = 10.34 (b, *NH*, 2 H, **ε**), 9.18 (b, 1 H, **χ**), 8.76 (b, *NH*, 2 H, **ο**), 8.42 (d, *J* = 7.8 Hz, 2 H, **β**), 8.31 (d, *J* = 1.4 Hz, 2 H, **σ**), 8.09 (t, *J* = 7.8 Hz, 1 H, **α**), 7.98 (s, 2 H, **m**), 7.64 (d, *J* = 8.8 Hz, 4 H, **o**), 7.51 (d, *J* = 4.7 Hz, 2 H, **d**), 7.45 (d, *J* = 8.8 Hz, 4 H, **p**), 7.25-7.30 (m, 24 H, **t & u**), 7.20-7.23 (m, 6 H, **v**), 6.83 & 6.82 (2x s, 8 H, **ι&ι’**), 6.17 (d, *J* = 4.7 Hz, 2 H, **e**), 4.62 (s, 4 H, **k**), 3.72 (t, *J* = 5.4 Hz, 4H, **j**), 3.58 (t, *J* = 5.4 Hz, 4H, **i**), 3.49 (q, *J* = 7.2 Hz, 4 H, **g**), 2.20–2.37 (m, 8 H, **μ**), 2.07 & 2.06 (2x s, 24 H, **θ&θ’**), 1.61 (m, 8 H, **ν**), 1.49 (m, 4 H, **ξ**), 1.39 (s, 9 H, **φ**), 1.19 (t, *J* = 7.2 Hz, 6 H, **h**) ppm.

^**13**^**C{**^**1**^**H} NMR** (175 MHz, CD_2_Cl_2_): δ = 179.2, 169.9, 167.7, 164.7, 162.6, 153.7, 149.7, 148.4, 147.9, 147.4, 146.7, 145.2, 139.3, 137.9, 135.4, 135.2, 135.0, 134.5, 132.7, 131.8, 131.6, 131.3, 128.8, 128.2, 126.6, 125.7, 125.4, 125.1, 123.3, 121.4, 120.0, 114.1, 109.3, 67.7, 65.3, 64.8, 53.8, 50.3, 45.2, 35.6, 34.7, 31.5, 30.1, 26.8, 23.4, 19.1, 19.0, 12.1 ppm.

**HRMS (ESI):** m/z calculated for C_139_H_137_N_13_O_8_S_2_ [M]^●+^ 2180.0154, found 2180.0108.

### Electrochemical switching

Cyclic voltammetry (CV) (Figs. S26 and S27) and differential pulse voltammetry (DPV) (Fig. S27 and Table S2) were performed on an Autolab PGSTAT302N potentiostat using a three-electrode configuration: a freshly polished glassy carbon working electrode, a platinum wire counter electrode, and a silver wire pseudoreference electrode. All measurements were conducted at least three times and with a broad range of different scan rates (10–2000 mV/s) (Figs. S26 and S29). The decamethylferrocene/decamethylferrocenium ([FeCp_2_*]^+/0^) couple was used as internal reference. Dry and nitrogen-purged 1,2-dichloroethane (DCE) as solvent, *n*-Bu_4_NPF_6_ (0.1 M) as electrolyte, and an analyte concentration of 1.0 mM were used for all measurements. Binding of chloride to **Rot**^**0**^ in the presence of Bu_4_NPF_6_ was confirmed by−ESI HRMS (Fig. S28). The error of redox potentials derived by CV and DPV measurements is estimated to be smaller than ± 10 mV.

Digital simulations (Fig. S29) were performed with the DigiElch 8 Professional software package (ElchSoft GbR) using the Butler-Volmer equation. The surface area of the working electrode was set to 0.05 cm^2^, and the starting concentration of analytes was set to 1.0 mM. The charge-transfer coefficients α were set to the initial value of 0.5, and the heterogeneous rate constants *k*_s_ were estimated by the peak separation and set between 0.01 and 0.1 cm/s. The diffusion coefficient was set to its default value of 1 × 10^-5^ cm^2^/s. The fitting model for the experimental CV data is based on an (EE)EC_i_ mechanism for the direction of a cathodic sweep (starting from the +2 oxidation state). The reaction kinetics, i. e. the rate constant of the chemical reaction step *k*_f_, of **Ax**^**●−**^ and **Rot**^**●−**^ were evaluated according to an irreversible (pseudo)first-order reaction based on four different scan rates (25, 100, 250, and 1000 mV/s).

### Quantum chemical calculations

While the available crystal structure of **Ax** could be used as a starting point for the investigation of **Ax**, for **Rot** a search of the conformational landscape was undertaken using Stefan Grimme’s GFN2-xTB^65^ and CREST^66^ codes. All subsequent structure optimizations were performed using the Turbomole program package^71^ (version 7.6) employing the PBEh-3c^72^ composite method in combination with the COSMO^73^ solvent model. Single point electronic energies were evaluated at the PBE0-D3(BJ)^74,75^, B3LYP-D3(BJ)^76^, BHLYP-D3(BJ)^77^, and ωB97X-D3^78^ levels of density functional theory, all in addition with the SMD^79^ solvent model with keyword “dichloromethane”, utilizing the ORCA program suite (version 5.0.4)^80^. A def2-QZVP^81^ basis set was used for **Ax**, a def2-TZVP basis set for **Rot** and the stoppered axle. Optical transitions were calculated for **Ax** using the same density functional approximations, but with a def2-TZVP basis set. Transition states for the two rotational barriers in **Ax** were obtained by manually displacing the respective dihedral angle and subsequent transition state optimization following the lowest eigenvalue of the Hessian (“imaginary frequency”). Negative eigenvalues above −20 cm^-1^ were neglected when assessing the quality of the transition state structures after a run had converged. Transition state runs were performed with charges 0 and +2. All other calculations were done with charges -1, 0, +1, and +2, respectively.

## Supplementary information

supplementary information

Description of Additional Supplementary Files

Supplementary Data 1

Supplementary Data 2

Supplementary Data 3

Supplementary Data 4

Supplementary Data 5

## Data Availability

Crystallographic data for the structures reported in this article have been deposited at the Cambridge Crystallographic Data Centre, under deposition numbers CCDC 2332801 (**Ax**) and 2332802 (**psRot**). Copies of the data can be obtained free of charge via https://www.ccdc.cam.ac.uk/structures/. All other relevant data generated and analyzed during this study, which include experimental, spectroscopic, crystallographic and computational data, are included in this article and the Supplementary Data 1 file. XRD structure files for **Ax** (Dataset 1, Checkcif Dataset 3), and for **psRot** (Dataset 2, Checkcif Dataset 4) are attached. All calculated structures are contained in Dataset 5.
