# Supplementary material for: Switchable protection and exposure of a sensitive squaraine dye within a redox active rotaxane
Source: Commun Chem. 2024 Oct 4;7:229. doi: 10.1038/s42004-024-01312-1 (PMC11452610; doi:10.1038/s42004-024-01312-1)
Supplement: Supplementary file 1 — supplementary information [file 42004_2024_1312_MOESM1_ESM.pdf]

Supporting Information - Switchable Protection and Exposure of a Sensitive Squaraine Dye within a Redox Active Rotaxane

Janos Wasternack[1], Hendrik V. Schröder[1], J. Felix Witte[2], Mihkel Ilisson[1], Henrik Hupatz[1], Julian F. Hille[1], Marius Gaedke[1], Arto M. Valkonen[3], Sebastian Sobottka[4], Alexander Krappe[5], Mario Schubert[6], Beate Paulus[2], Kari Rissanen[3], Biprajit Sarkar[4,7], Siegfried Eigler[5], Ute Resch-Genger[8] and Christoph A. Schalley[1]*

1 Institut für Chemie und Biochemie, Freie Universität Berlin, Arnimallee 20, 14195 Berlin, Germany
2 Institut für Chemie und Biochemie, Freie Universität Berlin, Arnimallee 22, 14195 Berlin, Germany
3 University of Jyvaskyla, Department of Chemistry, Survontie 9 B, 40014 Jyväskylä, Finland
4 Institut für Chemie und Biochemie, Freie Universität Berlin, Fabeckstr. 34-36, 14195 Berlin, Germany
5 Institut für Chemie und Biochemie, Freie Universität Berlin, Altensteinstraße 23A, 14195 Berlin, Germany
6 Institut für Chemie und Biochemie, Freie Universität Berlin, Takustraße 3, 14195 Berlin, Germany
7 Institut für Anorganische Chemie, Universität Stuttgart, Pfaffenwaldring 55, 70569 Stuttgart, Germany
8 Bundesanstalt für Materialforschung und -prüfung (BAM), Biophotonics, Richard Willstätter Straße 11, 12489, Berlin, Germany
* Correspondence: Christoph A. Schalley: c.schalley@fu-berlin.de

Table of Contents

Section S1: NMR and HRMS Spectra 1

Section S2: Affinity studies for **Ax^0**, **TLM**, **psRot**, **Rot^0** & **Rot^0⊃Cl$^-$** 14

Section S3: Additional UV-vis and Fluorescence data 17

Section S4: Single crystal X-ray diffraction (SCXRD) 18

Section S5: Electrochemical Data 20

Section S6: Data on Oxidized Axle **Ax^{2+}** 22

Section S7: Data on Oxidized Rotaxane **Rot^{2+}** 25

Section S8: Low temperature ^{1}H NMR studies of **Ax^0** & **Rot^0** 29

Section S9: DFT Calculations 30

Section S10: Stability measurements of **Ax^0**, **Rot^0**, **Ax^{2+}** and **Rot^{2+}** 33

References 34

Section S1: NMR and HRMS Spectra

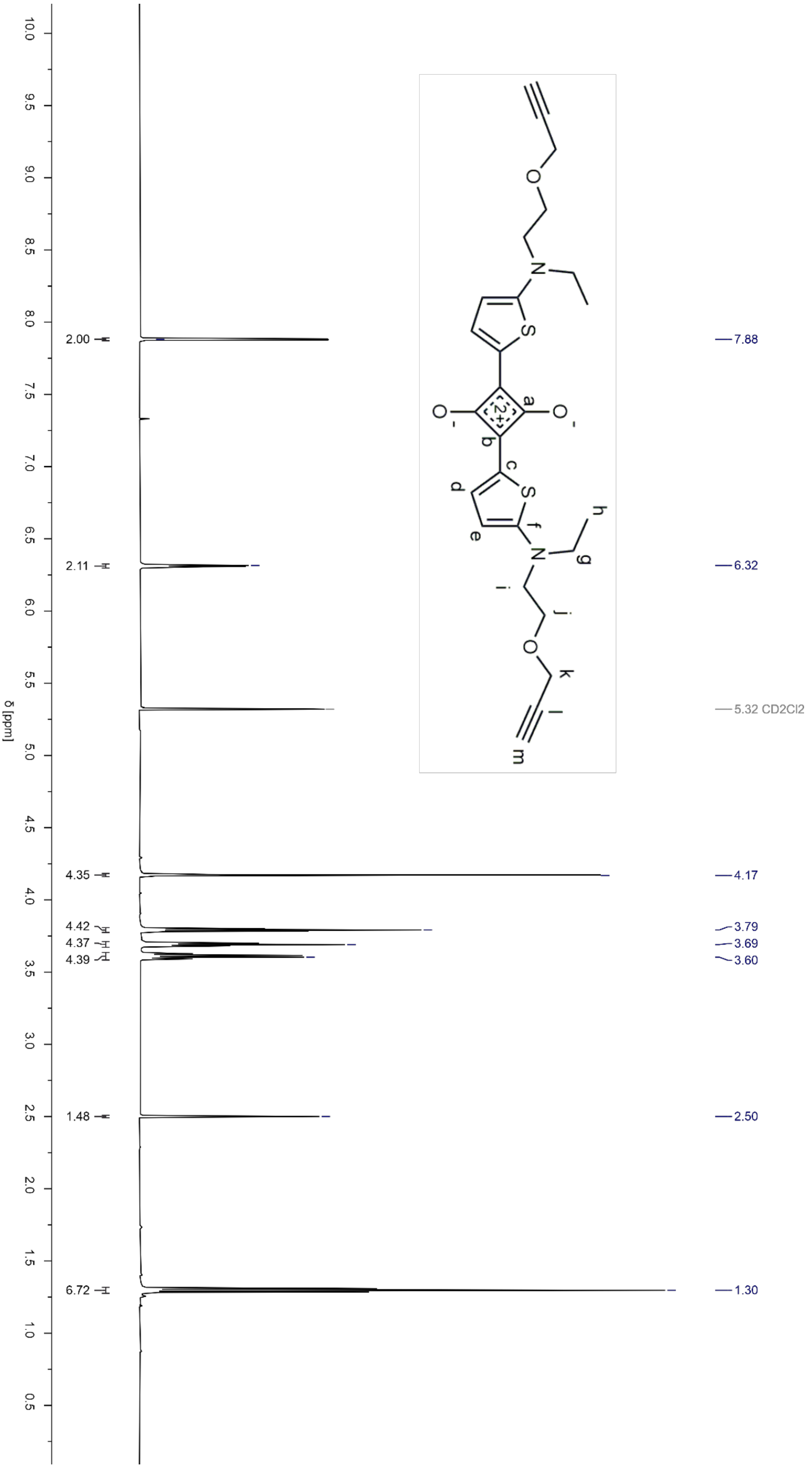

*Figure S1: ^{1}H NMR-spectrum of **Ax⁰**, 600 MHz, CD_2Cl_2, 298 K.*

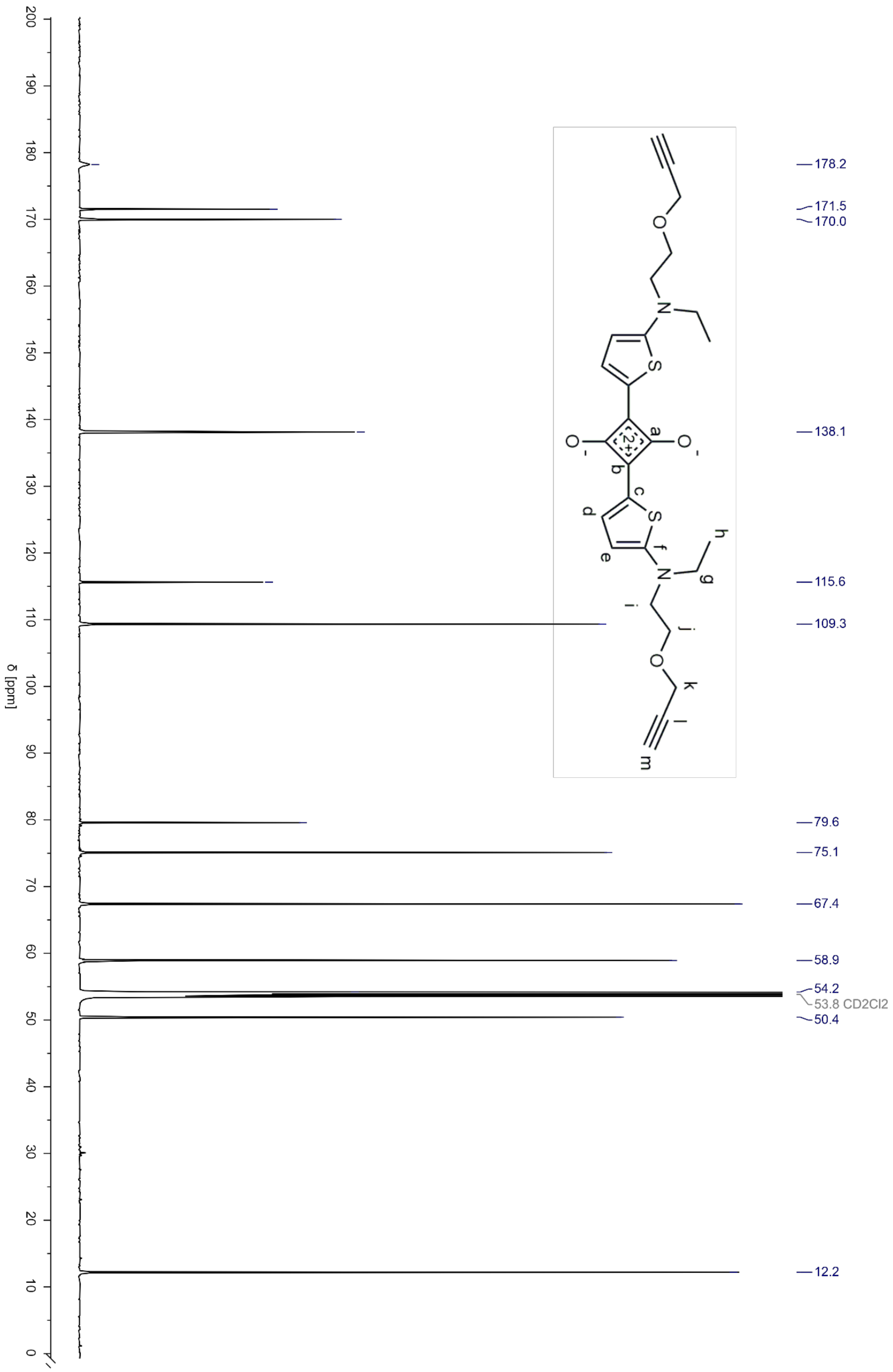

*Figure S2: $^{13}C\{^{1}H\}$ NMR-spectrum of **Ax⁰**, 151 MHz, CD_2Cl_2, 298 K.*

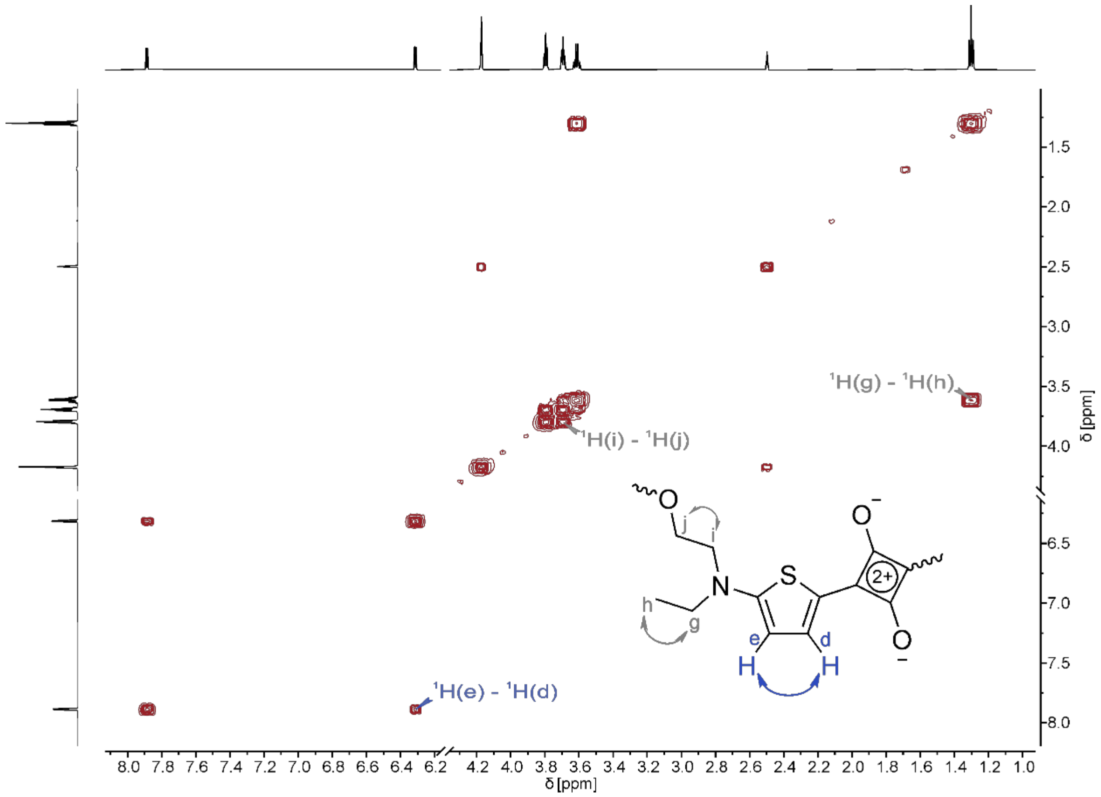

*Figure S3: ¹H,¹H COSY spectrum of **Ax⁰**, 600 MHz, CD₂Cl₂, 298 K, 4 scans, 2048 x 1640 complex points, recycle delay 1.5 s.*

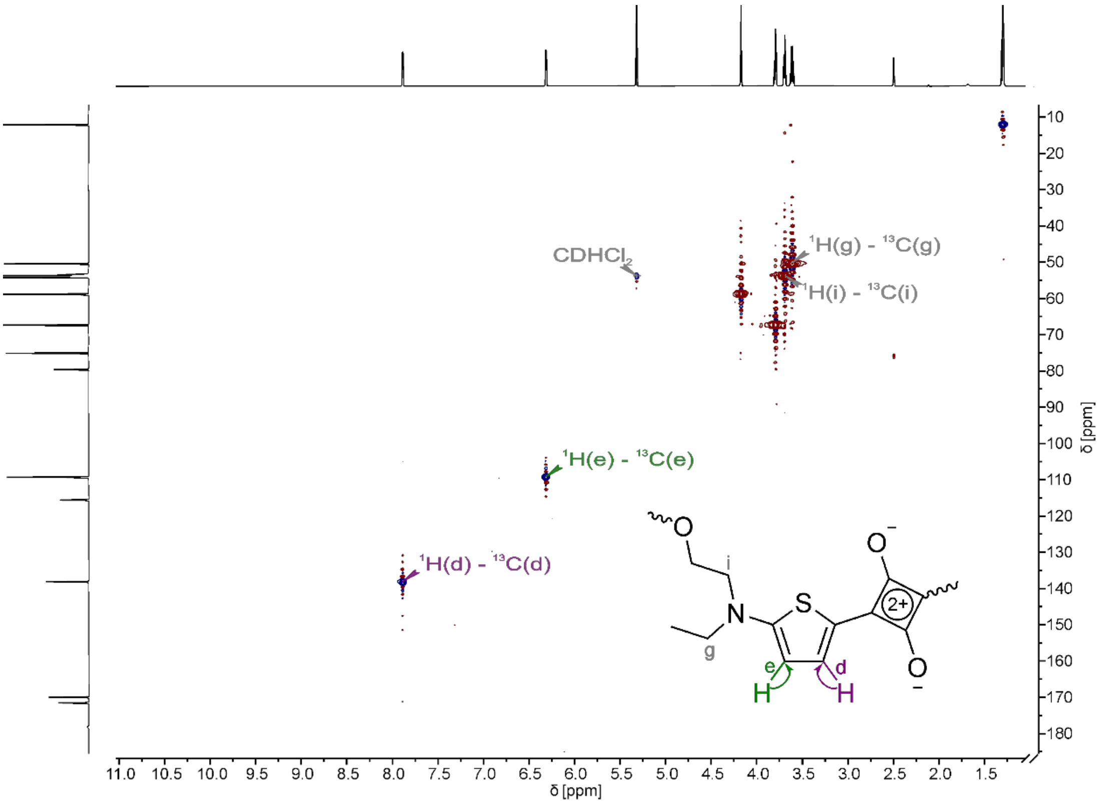

*Figure S4: ¹H,¹³C HSQC spectrum of **Ax⁰**, 600 MHz, CD₂Cl₂, 298 K, 2 scans, 1024 x 820 complex points, recycle delay 1.5 s.*

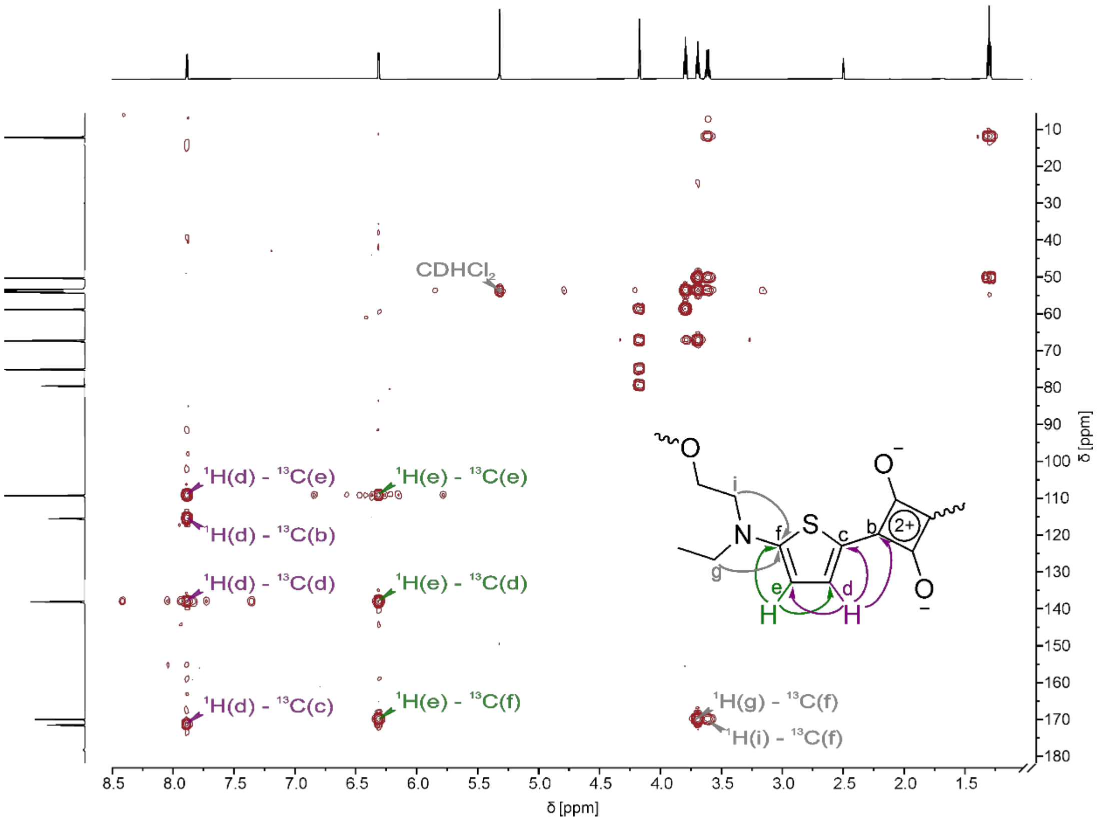

*Figure S5: $^{1}H,^{13}C$ HMBC spectrum of **Ax⁰**, 600 MHz, CD_2Cl_2, 298 K, 4 scans, 1024 x 820 complex points, recycle delay 1.5 s.*

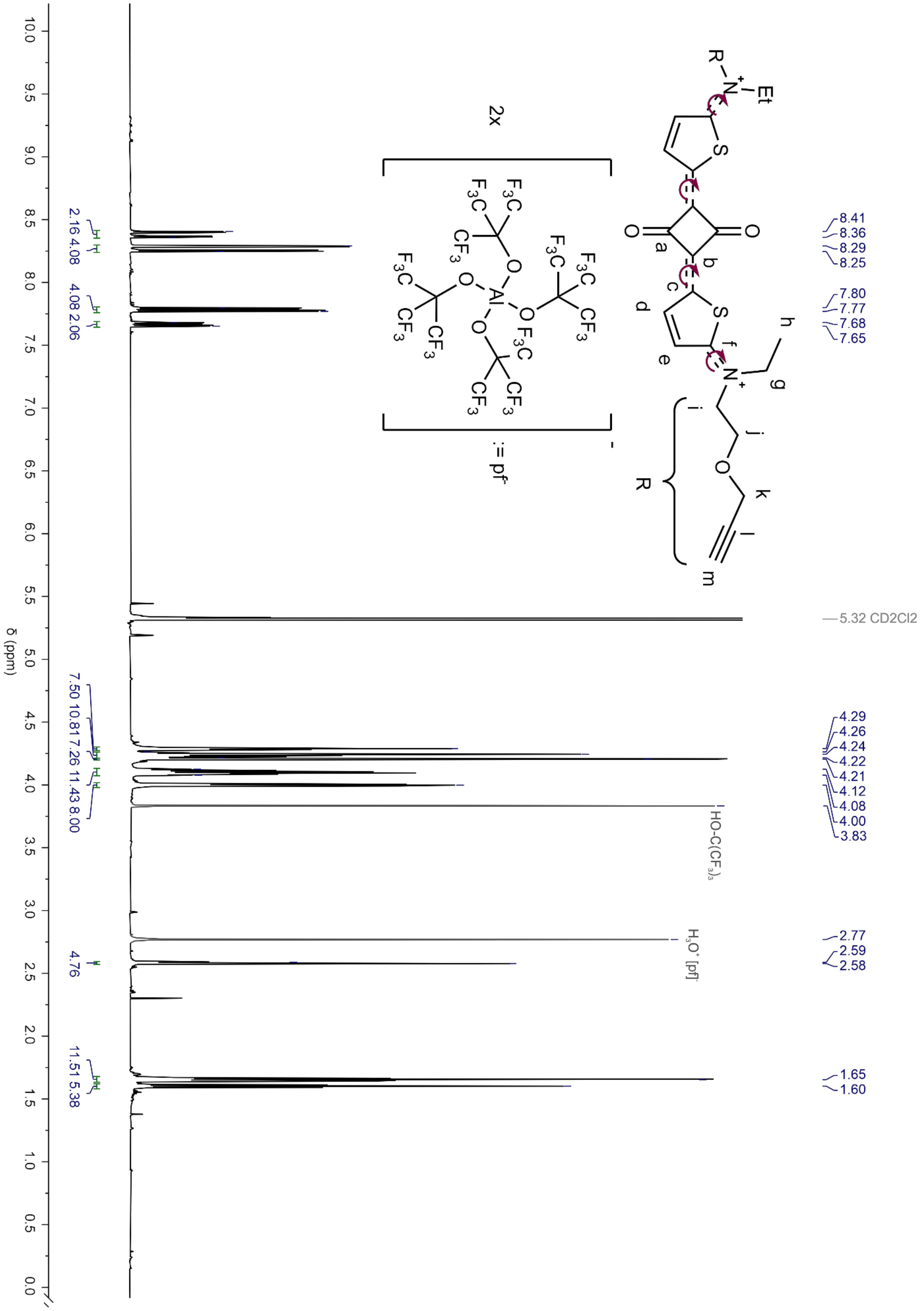

*Figure S6: ^{1}H NMR-spectrum of **Ax^{2+}**, 700 MHz, CD_2Cl_2, 298 K.*

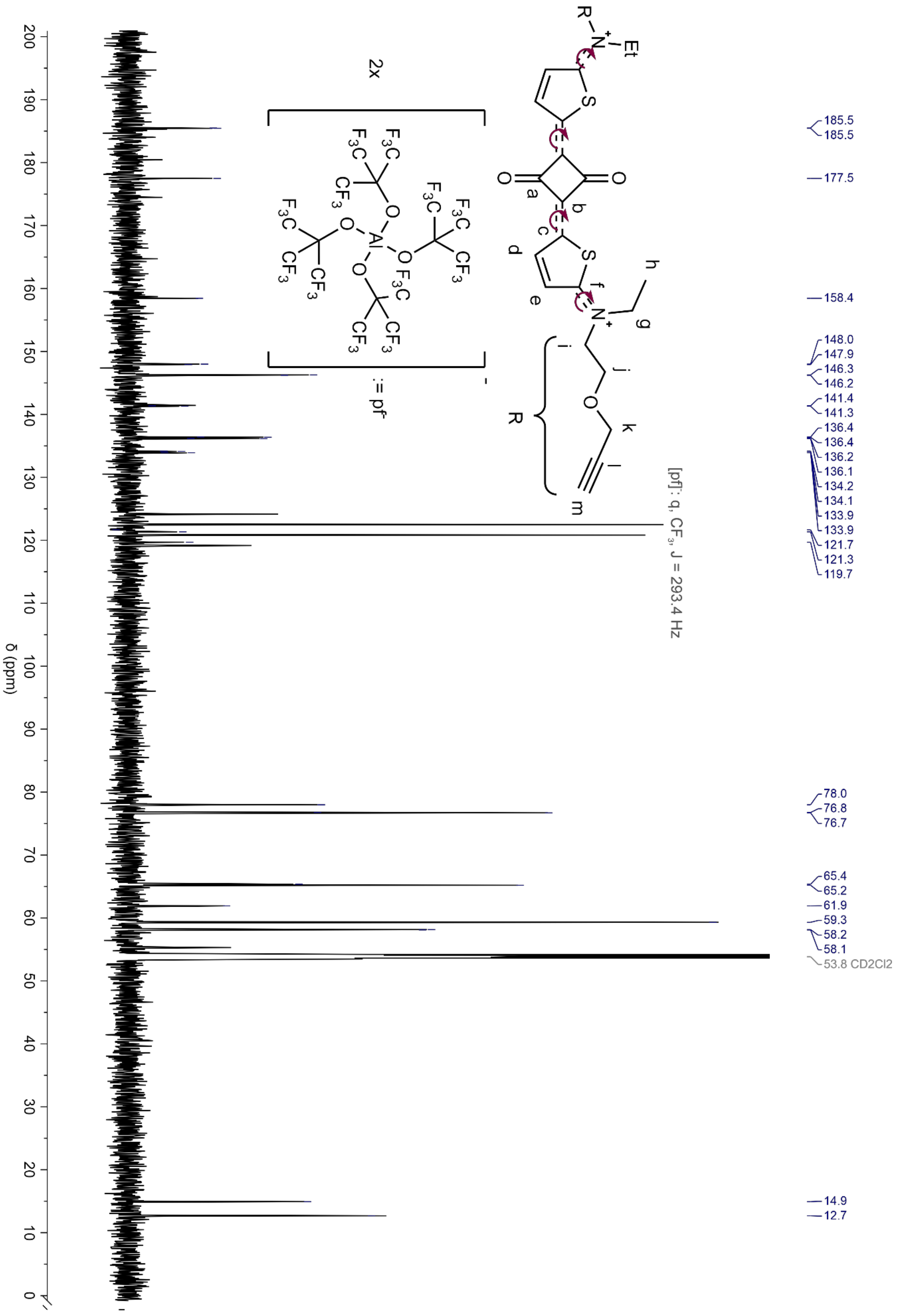

Figure S7: $^{13}C\{^{1}H\}$ NMR-spectrum of ***Ax***$^{2+}$*, 175 MHz, CD_2Cl_2, 298 K.*

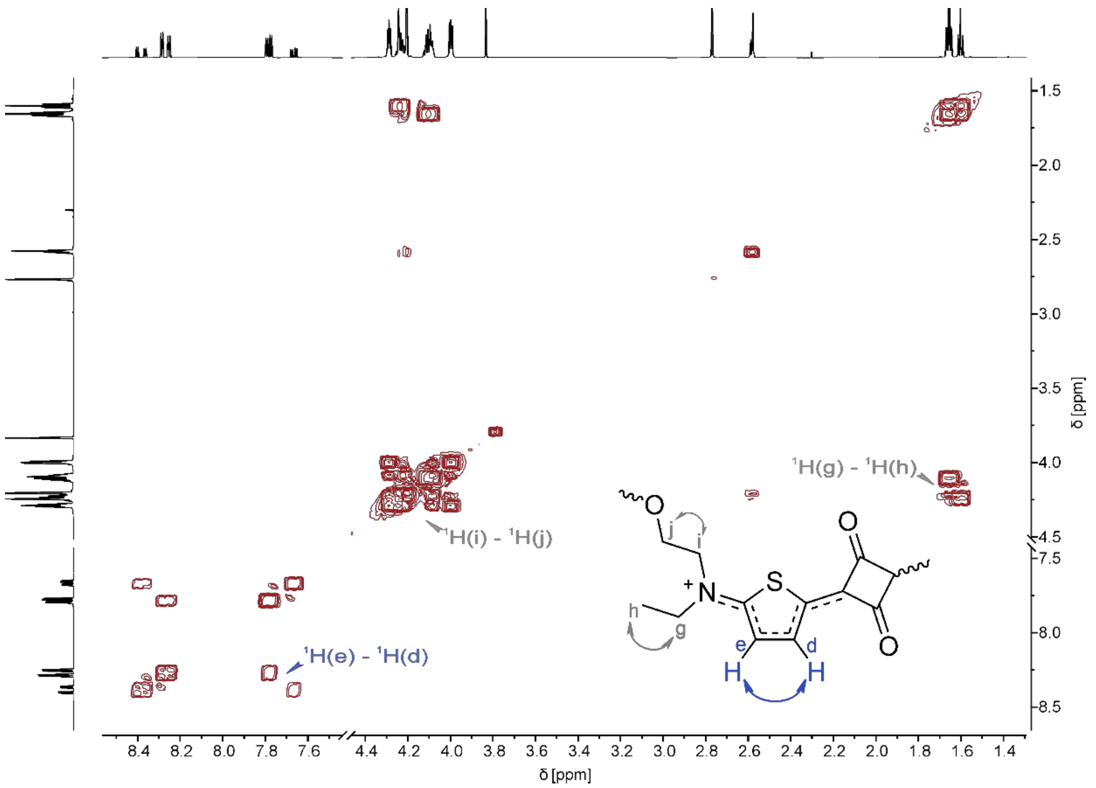

*Figure S8: ^{1}H,^{1}H COSY spectrum of **Ax^{2+}**, 600 MHz, CD_2Cl_2, 298 K, 4 scans, 2048 x 1640 complex points, recycle delay 1.5 s.*

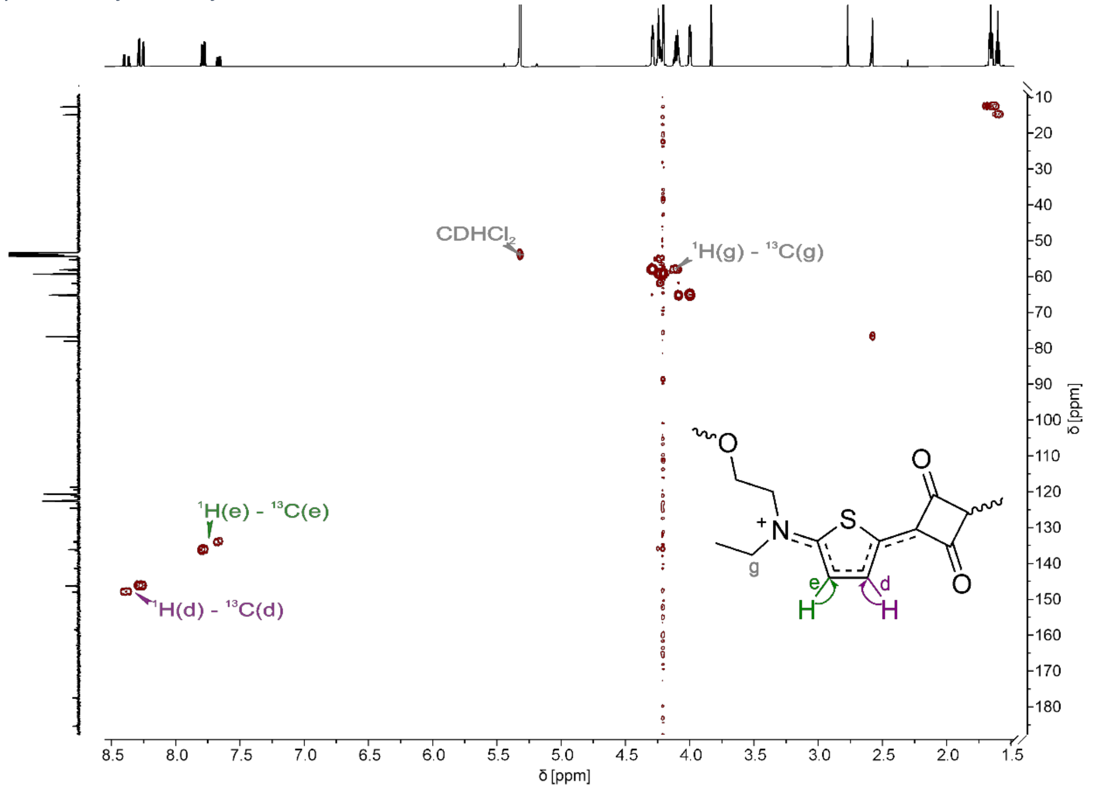

*Figure S9: ^{1}H,^{13}C HMQC spectrum of **Ax^{2+}**, 700 MHz, CD_2Cl_2, 298 K, 4 scans, 1024 x 1024 complex points, recycle delay 1.0 s.*

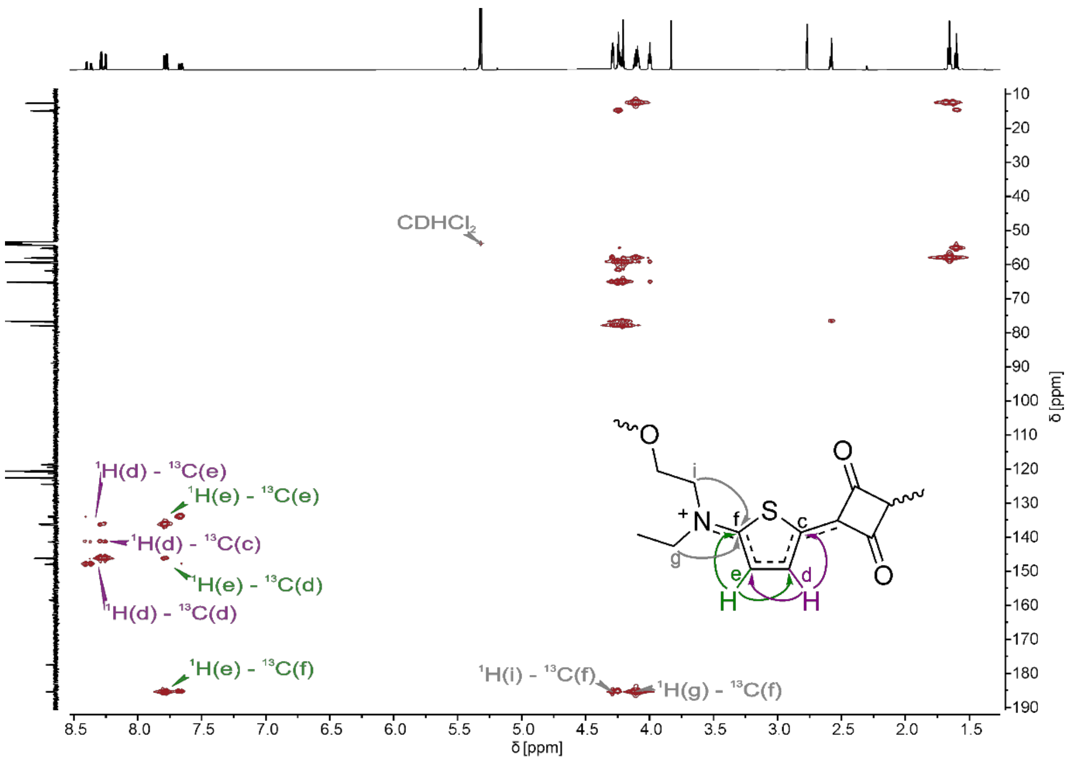

*Figure S10: $^{1}H,^{13}C$ HMBC spectrum of **Ax^{2+}**, 700 MHz, CD_2Cl_2, 298 K, 4 scans, 1024 x 1024 complex points, recycle delay 1.5 s.*

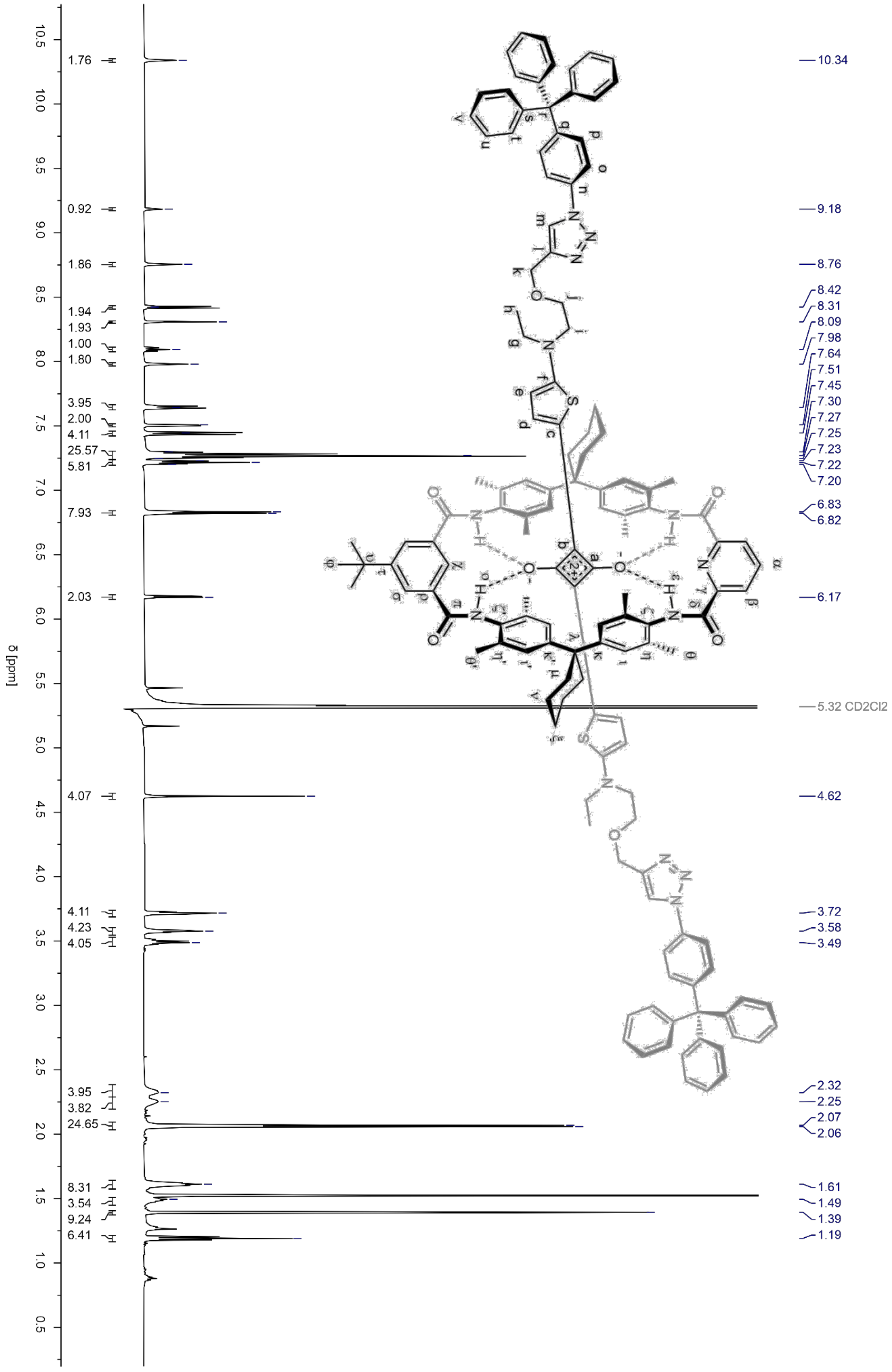

*Figure S11: ^{1}H NMR-spectrum of **Rot⁰**, 600 MHz, CD_2Cl_2, 298 K.*

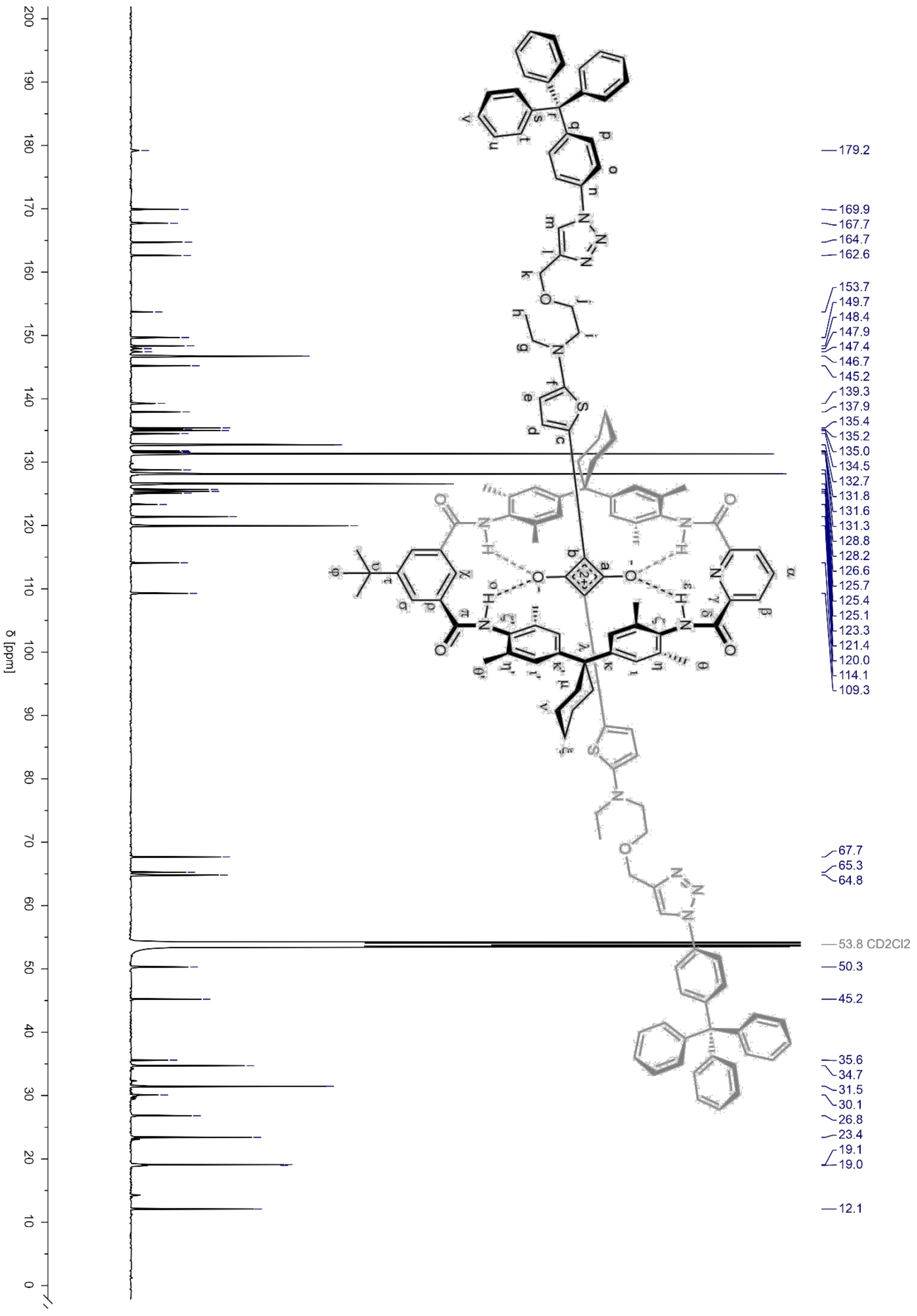

*Figure S12: $^{13}C\{^1H\}$ NMR-spectrum of **Rot^0**, 151 MHz, CD_2Cl_2, 298 K.*

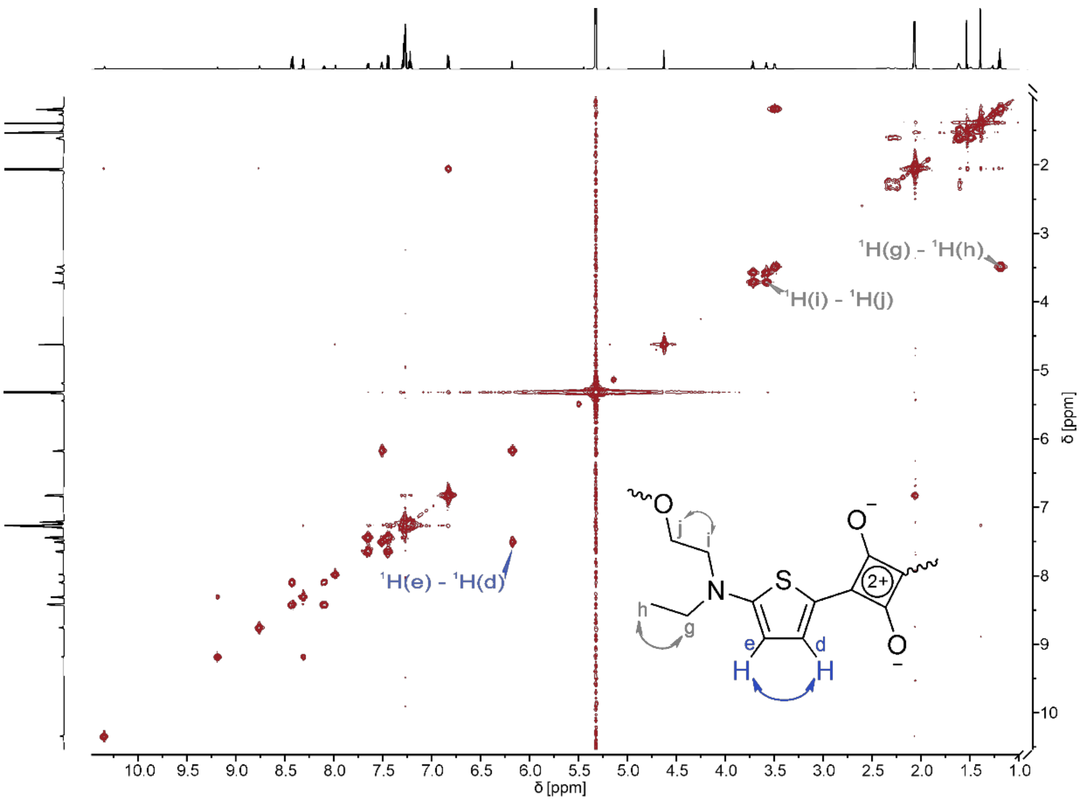

*Figure S13: 1H,1H COSY spectrum of **Rot⁰**, 500 MHz, CD_2Cl_2, 298 K, 8 scans, 1024 x 1024 complex points, recycle delay 1.5 s.*

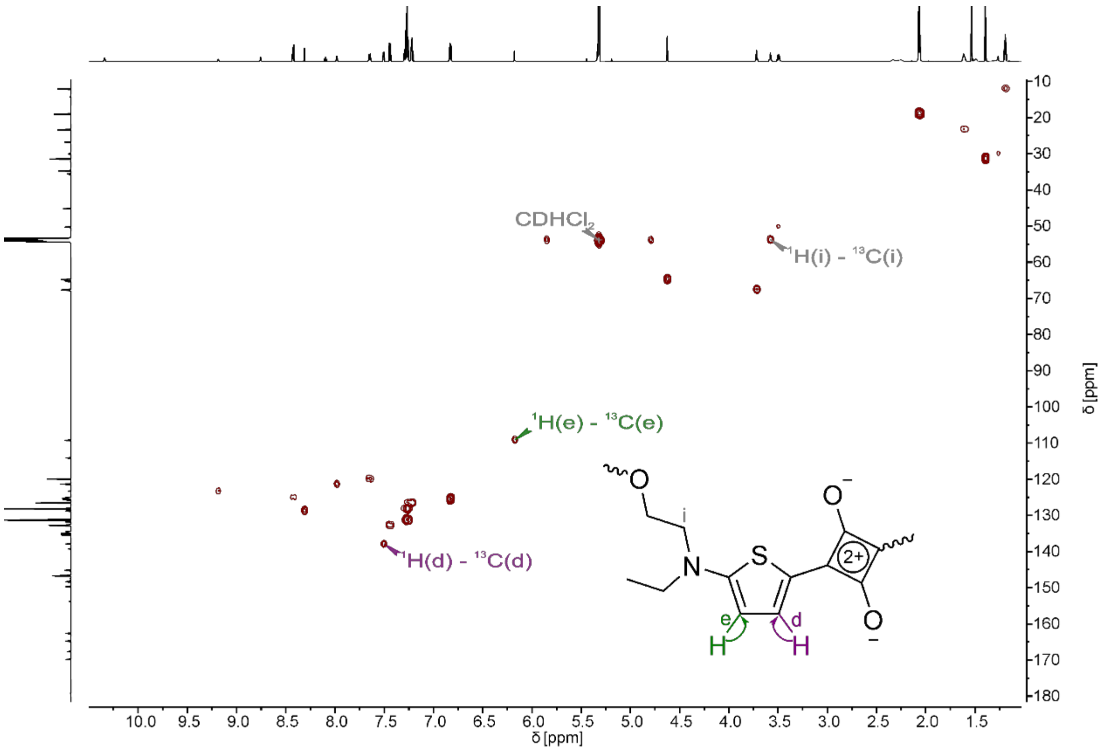

*Figure S14: 1H,13C HMQC spectrum of **Rot⁰**, 600 MHz, CD_2Cl_2, 298 K, 12 scans, 1024 x 820 complex points, recycle delay 1.5 s.*

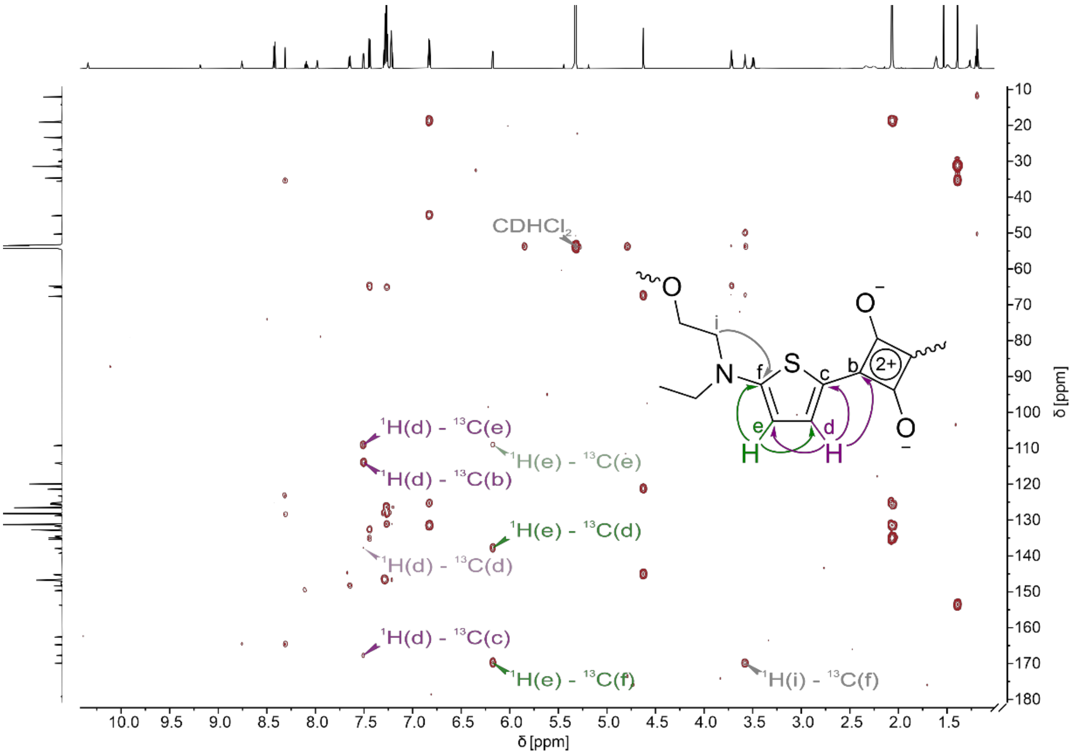

*Figure S15: $^{1}H,^{13}C$ HMBC spectrum of **Rot⁰**, 600 MHz, CD_2Cl_2, 298 K, 12 scans, 1024 x 820 complex points, recycle delay 1.5 s.*

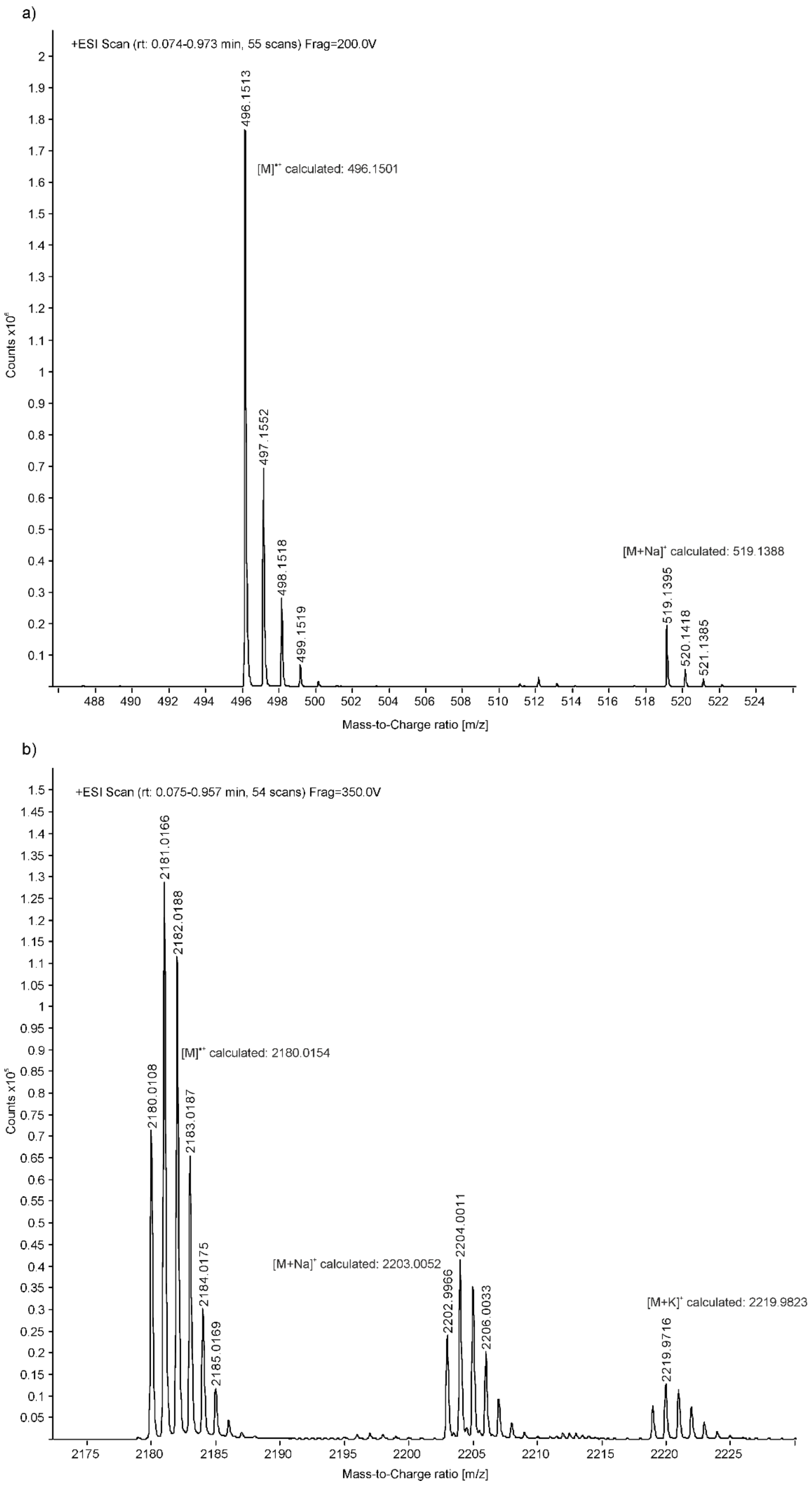

*Figure S16: HRMS spectra of a) **Ax** and b) **Rot**.*

Section S2: Affinity Studies for **Ax⁰**, **TLM**, **psRot**, **Rot⁰** & **Rot⁰⊃Cl⁻**

For the ^{1}H NMR titration of **Ax^0** and **TLM**, 13 samples were measured, each with constant c(**TLM**) = 4 mM and variation of c(**Ax^0**) = 0 – 40 mM in CD_2Cl_2. The change in chemical shift of the aromatic proton signal **σ** of **TLM** (8.19 ppm in pure **TLM**) was monitored and fitted using a 1:1 binding model.[1]

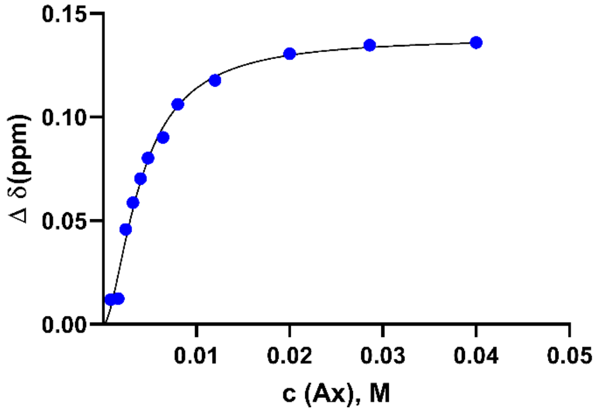

*Figure S17: Fit of the ^{1}H NMR titration of **Ax^0** and **TLM** to form **psRot** to determine K_a(**TLM⊃Ax^0**) = 520 ± 160 M^{-1} in CD_2Cl_2, 298 K, 600 MHz.*

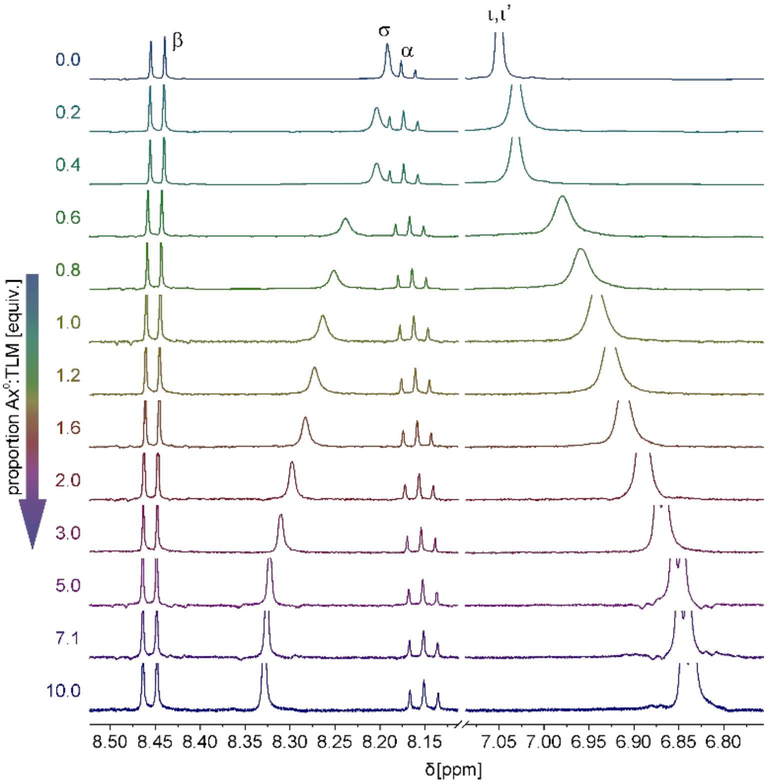

*Figure S18: Stacked partial ^{1}H NMR spectra of the titration of **Ax^0** and **TLM** to form **psRot**.*

For the ^{1}H NMR titration of **Rot⁰** and *n*-Bu_4NCl, **Rot⁰** was dissolved in CD_2Cl_2 (c(**Rot⁰**) = 2.2 mM) and a solution of *n*-Bu_4NCl in CD_2Cl_2 with c(*n*-Bu_4NCl) = 200 mM was titrated to the sample with a HAMILTON microsyringe. The change in chemical shift of the amide NH proton signal **o** (8.76 ppm in pure **Rot⁰**) was monitored and fitted using a 1:1 binding model.[1]

Additionally, a global analysis using the signals **o**, **ε** and **σ** was done using *bindfit*[2–4], providing K_a(**Rot⁰**⊃Cl^-) = 84 ± 12 M^{-1}.

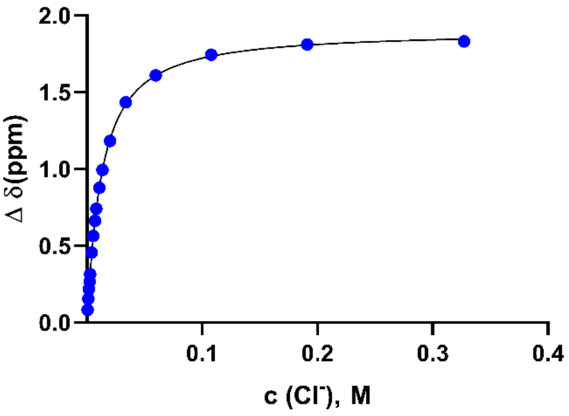

*Figure S19: Fit[1] of the ^{1}H NMR titration of **Rot⁰** with n-Bu_4NCl using the signal **o** to determine K_a(**Rot⁰**⊃Cl^-) = 88 ± 8 M^{-1} in CD_2Cl_2, 298 K, 600 MHz.*

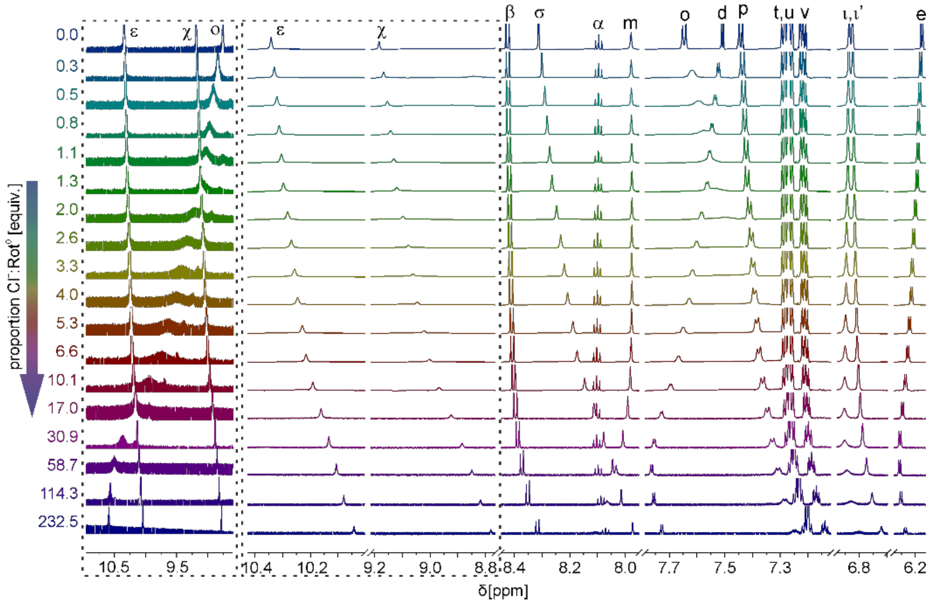

*Figure S20: Stacked partial ^{1}H NMR spectra of the titration of **Rot⁰** and n-Bu_4NCl. For better visibility of the NH signal **o,** the region between 11 - 9 ppm is depicted on the left with increased intensity.*

For the fluorescence titration, a solution of **Rot^0** in CH_2Cl_2 (c(**Rot^0**) = 310 nM) was prepared. The emission spectra were recorded and the emission peak at 677 nm was integrated. Next, a solution of *n*-Bu_4NCl and **Rot^0** in CH_2Cl_2 (c(*n*-Bu_4NCl) = 100 mM, c(**Rot^0**) = 310 nM) was added in portions, spectra were recorded, and the emission peak was integrated after each addition. Data for all titrations were fitted using least-squares non-linear fit.

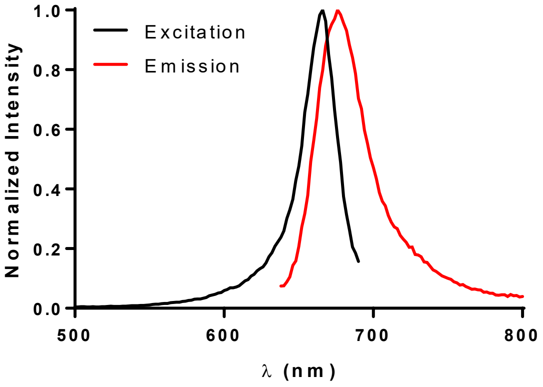

*Figure S21: Absorption and emission spectra of **Rot^0**, CH_2Cl_2, 298 K.*

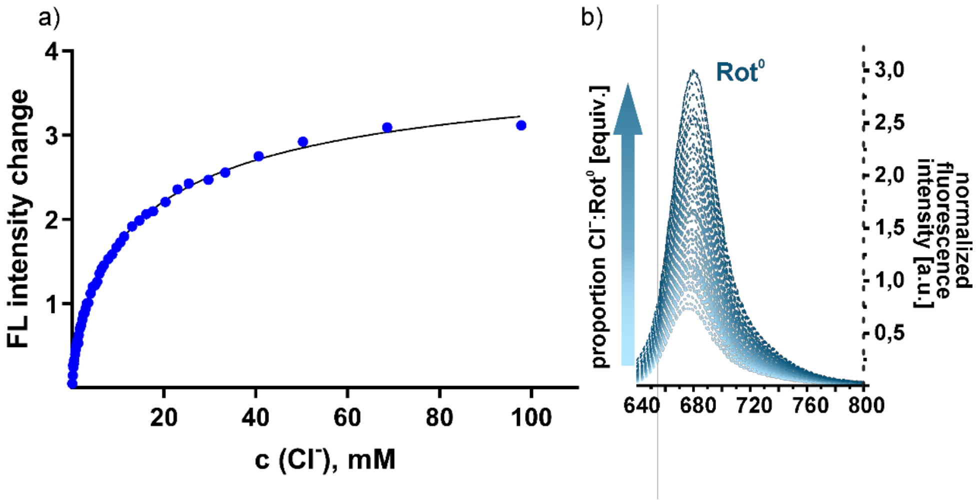

*Figure S22: a) Fit of the fluorescence titration of **Rot^0** with n-Bu_4NCl to determine K_a(**Rot^0**$\supset Cl^-$) = 90 ± 20 M^{-1} in CH_2Cl_2. b) Increasing fluorescence intensity during the titration.*

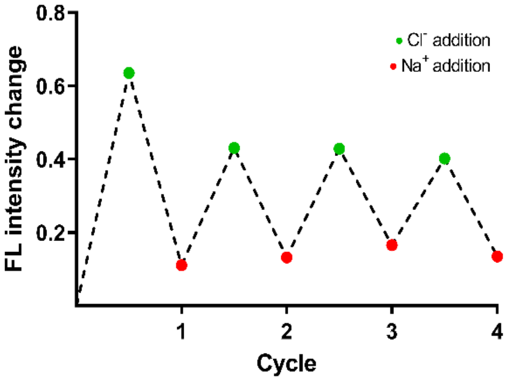

*Figure S23: The co-conformation of **Rot^0** can be reversibly switched by the addition of Cl^- (n-Bu_4NCl) and returned into the initial state by the addition of Na^+ ($Na[B(Ph3,5\text{-}(CF_3)_2)_4]$). Scattering caused by finely dispersed NaCl leads to the lowered intensity after the first cycle.*

Section S3: Additional UV-vis and Fluorescence data

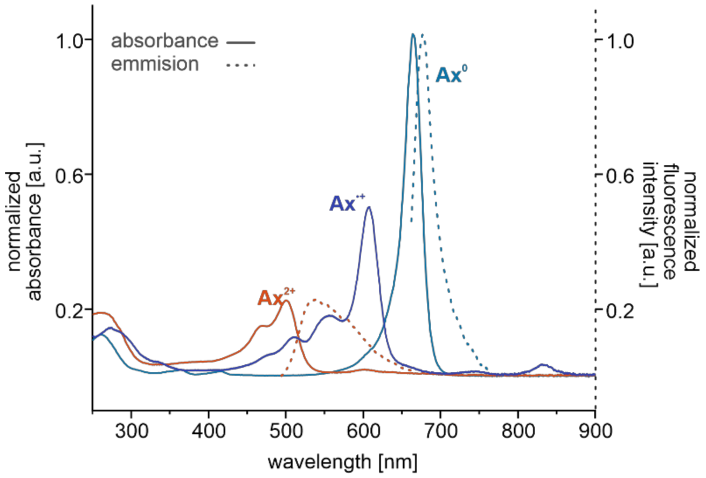

*Figure S24: UV-Vis (solid) spectra of **Ax** in three oxidation states and fluorescence (dashed) spectra of **Ax^0** and **Ax^{2+}**.*

*Table S1: Quantum yields of the fluorescent states of **Ax** & **Rot**.*

state	**Ax^0**	**Rot^0**	**Ax^0** + Cl^-	**Rot^0** + Cl^-	**Ax^{2+}**	**Rot^{2+}**
λ_{max} Emission [nm]	678	683	-	-	548	570
λ Excitation [nm]	630	630	630	630	500	500
Quantum yield Φ	**6.7 %**	**4.0 %**	**5.9 %**	**4.2 %**	**0.5%**	**0.6%**

Section S4: Single Crystal X-ray Diffraction (SCXRD)

Single crystals of **Ax^0** and pseudo[2]rotaxane **psRot** were obtained by slow evaporation of their dichloromethane solutions. In **psRot** there is 2-fold symmetry axis running through the squaraine moiety and only half of **psRot** is found in the asymmetric unit of the structure. This symmetry confirms the *trans* conformation for **Ax**, but on the other hand, the **TLM** macrocycle is not symmetric, and the crystallographic symmetry induces a huge disorder to the macrocycle. Thus, the macrocycle has two different opposite orientations in **psRot** and *tert*-butyl aryl location is equally alternated with pyridine + cocrystallized dichloromethane molecule.

The single crystal X-ray diffraction data were collected at 120(2) K with a Rigaku SuperNova dual wavelength diffractometer (Atlas CCD area detector) using mirror-monochromated Cu-Kα radiation (λ = 1.54184 Å). Data collection, reduction and gaussian (**Ax^0)** or analytical (**psRot**) face-index based absorption correction for all data were performed using CrysAlisPro[5]. All structures were solved using SHELXT[6] and refined by full-matrix least-squares on F^2 using SHELXL[7]. In all data, anisotropic displacement parameters were introduced for all atoms except hydrogen atoms. Hydrogens were calculated into their ideal positions (riding model), except amide N-H distances in **psRot**, which were set to a distance of 1.04 Å (instruction AFIX 43 1.04) previously determined for N-H by neutron diffraction.[8] Isotropic displacement parameters of 1.2 or 1.5 (for methyl groups) times that of the parent atom were used for all hydrogens. The figures were made with Mercury[9]. Only one floating origin restraint is applied in **Ax^0**, which was refined as a 2-component inversion twin. Moderate geometric anisotropic displacement parameter (ADP) restraints were applied to handle the refinement of heavily disordered **psRot**. X-ray crystallographic data, structural refinement parameters and thermal ellipsoid diagrams for all structures are reported below. Deposition numbers CCDC 2332801-2332802 contain the supplementary crystallographic data for this paper. These data are provided free of charge by the joint Cambridge Crystallographic Data Centre.

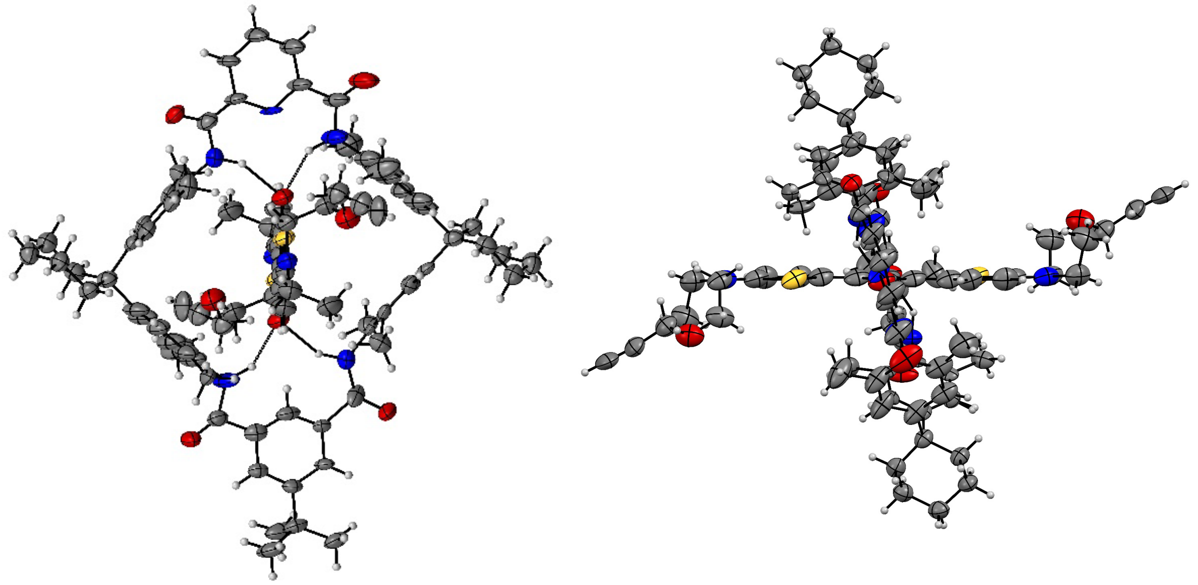

*Figure S25: SCXRD structure of **psRot** from different perspectives (thermal displace parameters at 50% probability level). The macrocycle is positionally disordered in the crystal and the pyridine dicarboxylic amide and isophthalic diamide can replace each other in their positions.*

Crystal data and refinement parameters.

Ax⁰ (CCDC 2332801): Crystal size 0.13x0.10x0.06 mm, $C_{26}H_{28}N_3O_4S_2$, M = 496.62, monoclinic, space group $P2_1$, a = 9.6863(3) Å, b = 10.8682(3) Å, c = 11.4392(3) Å, β = 94.582(2)°, V = 1200.39(6) Å^3, Z = 2, ρ = 1.374 g cm^{-3}, μ = 3.309 mm^{-1}, F(000) = 524, 4661 reflections ($2\theta_{max}$ = 146.872°) measured (3414 unique, R_{int} = 0.0221, completeness = 99.9 %), final R indices ($I > 2\sigma(I)$): R_1 = 0.0335, wR_2 = 0.0865, R indices (all data): R_1 = 0.0353, wR_2 = 0.0882. GOF = 1.030 for 308 parameters and 1 restraint, largest diff. peak and hole 0.279/−0.260 eÅ^{-3}.

psRot (CCDC 2332802): Crystal size 0.26x0.15x0.12 mm, $C_{89}H_{99}N_7O_8S_2{\cdot}CH_2Cl_2$, M = 1543.79, monoclinic, space group $P2_1/n$, a = 16.5888(4) Å, b = 15.0832(4) Å, c = 16.7501(3) Å, β = 98.607(2)°, V = 4143.88(17) Å^3, Z = 2, ρ = 1.237 g cm^{-3}, μ = 1.652 mm^{-1}, F(000) = 1640, 15124 reflections ($2\theta_{max}$ = 146.428°) measured (8047 unique, R_{int} = 0.0286, completeness = 99.5 %), final R indices ($I > 2\sigma(I)$): R_1 = 0.1006, wR_2 = 0.2526, R indices (all data): R_1 = 0.1119, wR_2 = 0.2605. GOF = 1.085 for 792 parameters and 290 restraints, largest diff. peak and hole 0.824/−0.382 eÅ^{-3}.

Section S5: Electrochemical Data

Electrochemical Switching

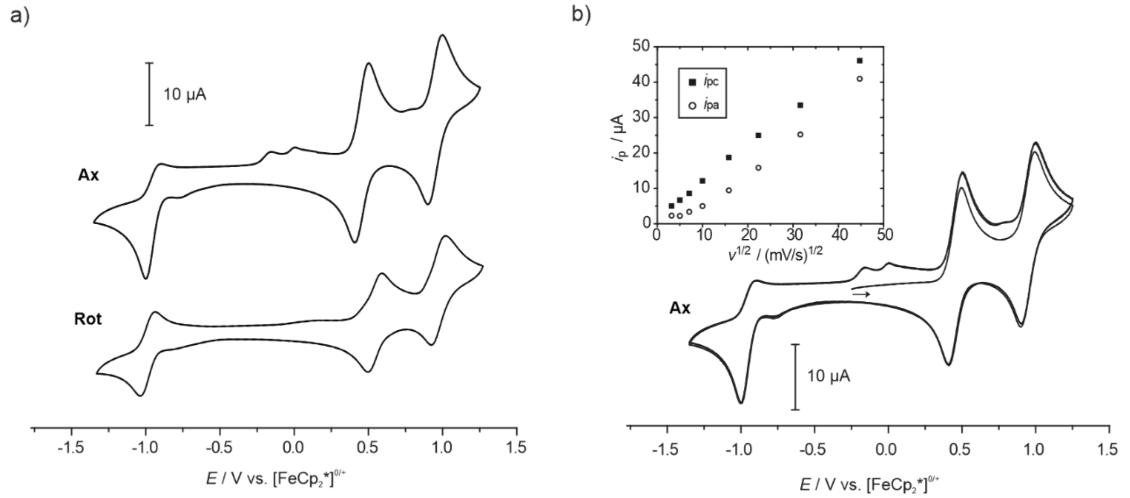

*Figure S26: a) Cyclic voltammograms (100 mV/s) of **Ax** and **Rot**. The second scan cycle is shown. b) Cyclic voltammogram (100 mV/s) of **Ax**. Shown are the first three scan cycles. The starting potential of the first scan is indicated by an arrow. The inset shows a plot of cathodic and anodic peak currents (i_{pc} and i_{pa}, respectively) against the square root of the scan rate for the reduction process. Note the linear relation of the i_{pc} values according to the RANDLES-ŠEVČÍK equation. The additional peaks at approximately −0.1 V in the second scan cycle, the i_{pa} / i_{pc} ratio, and the non-linear behavior of i_{pa} at different scan rates (inset) indicate an EC_i mechanism for the one-electron reduction of **Ax**.*

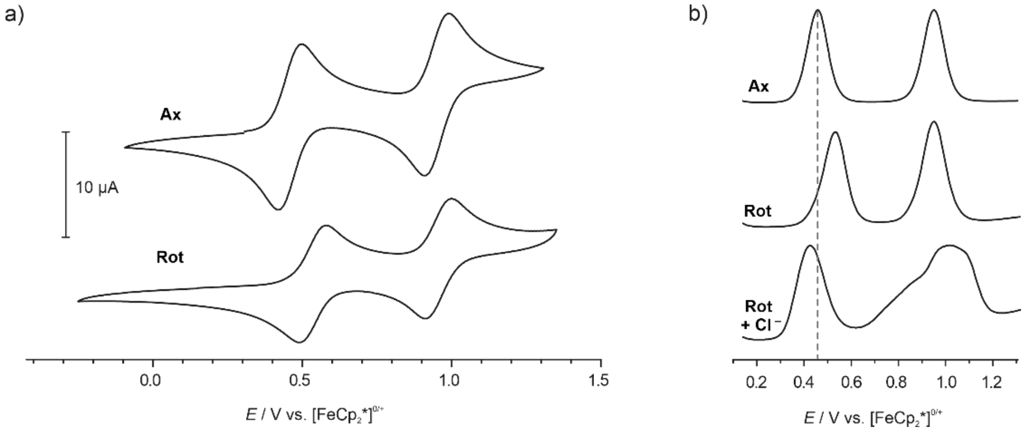

*Figure S27: a) Redox switching of **Rot** by reversible oxidation reactions: cyclic voltammograms (100 mV/s) of **Ax** and **Rot**. (c) Differential pulse voltammograms (10 mV/s scan rate, 25 mV modulation amplitude, 50 ms modulation time, 5 mV step potential, 0.5 s interval time) of **Ax**, **Rot**, and **Rot** after addition of excess n-Bu_4NCl. The second oxidation reaction of **Rot** in presence of chloride ions appears to be irreversible in both DPV and CV.*

*Table S2: Redox potentials of **Ax**, **Rot**, and **Rot** after addition of excess n-Bu_4NCl obtained by DPV (10 mV/s scan rate, 25 mV modulation amplitude, 50 ms modulation time, 5 mV step potential, 0.5 s interval time) and CV (100 mV/s). The redox potentials have been referenced against the $[FeCp_2*]^{0/+}$ couple.*

	**$E_{1/2}^{-1}$ / V**	**$E_{1/2}^{1}$ / V**	**$E_{1/2}^{2}$ / V**
**Ax**	-0.99[1]	0.45	0.95
**Rot**	-0.99	0.54	0.96
**Rot** + 50 equiv. *n*-Bu_4NCl[2]	/	0.43	/

[1] Due to the non-reversible reduction of **Ax**, potentials are extracted from the cyclic voltammograms and given for the cathodic peaks (E_{pc}^{-1}). [2] Only the reversible first one-electron oxidation reaction is considered.

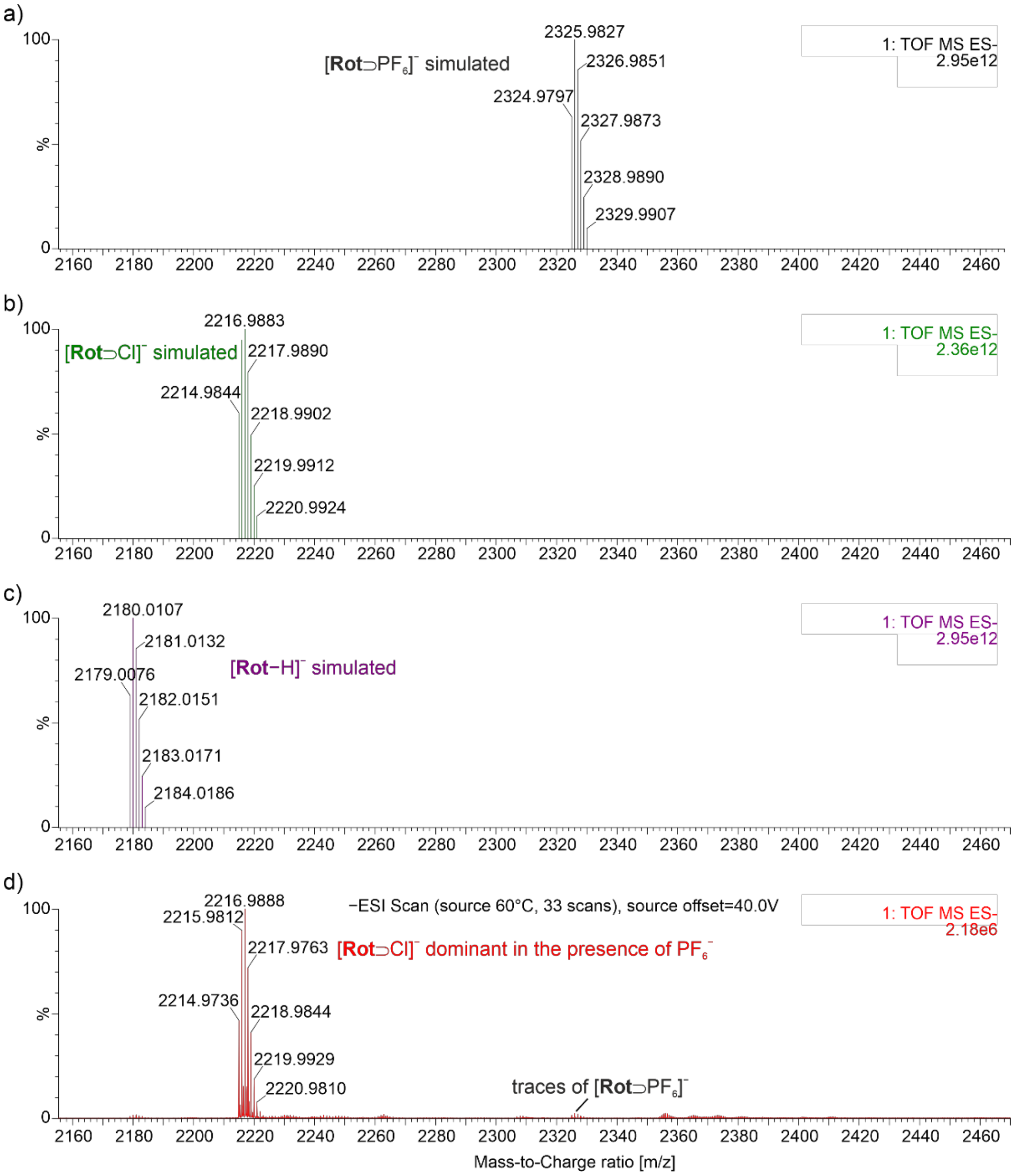

Figure S28: ESI-MS investigation of the binding of Cl^- to ***Rot*** *in the presence of PF_6^-. a) Simulated spectrum of [**Rot**⊃ PF_6]$^-$. b) Simulated spectrum of [**Rot**⊃ Cl]$^-$. c) Simulated spectrum of [**Rot**-H]$^-$. d) Measured spectrum of an equimolar mixture of **Rot⁰**, n-Bu_4NCl & n-Bu_4NPF_6 at c = 8 · 10^{-5} mol/L. The most intense signal in the spectrum is PF_6^-. The dominant species in the mass region [m/z 2000-2500] is the chloride adduct, while only traces of a PF_6^- are observed, clearly indicating the preferential binding of chloride in the presence of PF_6^-.*

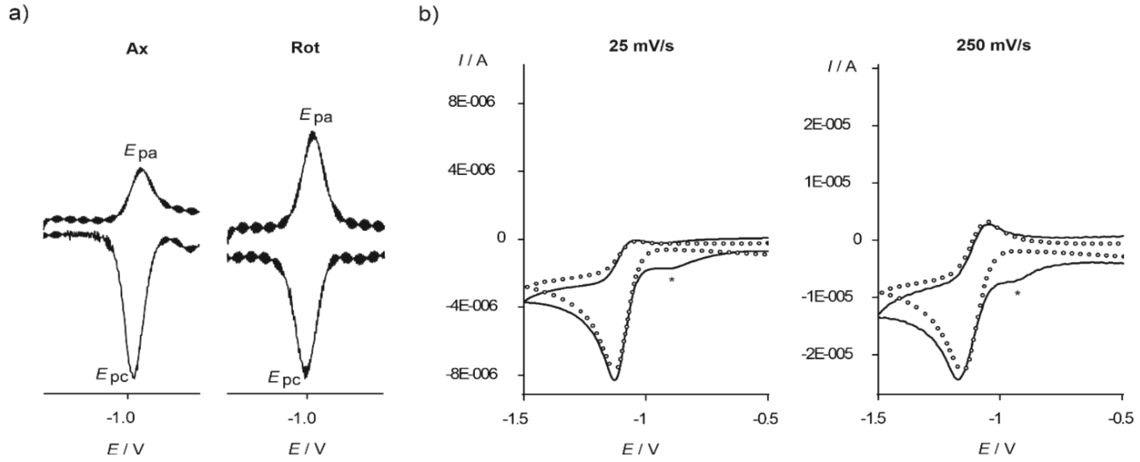

*Figure S29: a) Semi-differential plots, normalized on the cathodic peak current i_{pc}, based on cyclic voltammograms (100 mV/s) of **Ax** and **Rot**. The decreased anodic peak current (i_{pa}) for **Ax** compared to **Rot** indicates an irreversible chemical reaction and a stabilization of the radical anion by rotaxanation. b) Experimental data (line) and simulation (circles) of axle reduction at scan rates of 25 and 250 mV/s. The shoulder on the anodic side relative to the squaraine reduction peak is marked with an asterisk and addressed to an unknown decomposition product or traces of oxygen. It was not observed in a control measurement using only solvent and electrolyte.*

Electron Paramagnetic Resonance (EPR) Spectroelectrochemistry

EPR spectra at X-band frequency (ca. 9.5 GHz) were obtained with a Magnettech MS-5000 benchtop EPR spectrometer equipped with a rectangular TE 102 cavity. The measurements were carried out in quartz glass tubes. For EPR spectroelectrochemistry a three-electrode setup was employed: 1) a teflon-coated platinum wire (0.005” bare, 0.008” coated) as working electrode, 2) a teflon-coated platinum wire (0.005” bare, 0.008” coated) as counter electrode and 3) a Teflon-coated silver wire (0.005” bare, 0.007” coated) as pseudoreference electrode. Spectral simulations were carried out using EasySpin 6.0.0-dev.53 and MatLab 2023b.

Table S3: Hyperfine coupling constants and number of equivalent nuclei used for the EPR simulation.

Nucleus	Number of nuclei	A [mT]
^{14}N	2	0.30
^{1}H	2	0.33
^{1}H	2	0.21
^{1}H	8	0.16

Section S6: Data on Oxidized Axle **Ax**$^{2+}$

Axle **Ax**^{0} is oxidized by $NOPF_6$ (10 equiv.) in anhydrous dichloromethane and **Ax**$^{2+}$$[PF_6^-]_2$ separates as an oil from the mixture. Lithium tetrakis(perfluoro-^{tert}butoxy)aluminate (Li[pf]) (3.5 equiv.) was added to increase the solubility of the oxidized squaraine by anion metathesis with the weakly coordinating [pf$^-$] anion to form **Ax**$^{2+}$[pf$^-$]$_2$. The orange-red solutions of **Ax**$^{2+}$[pf$^-$]$_2$ in anhydrous dichloromethane are stable for days without signs of decomposition as investigated by UV-Vis or ^{1}H NMR spectroscopy.

Rotational barriers of the C^2-C^3-bonds and N-C^6-bonds in squaraines (Figure S30 and Table S4) are low in the Sq^0 state, resulting in fast interconversion of rotamers in solution on the NMR-timescale. However, oxidation into the Sq^{2+} state leads to a quinoid electronic structure (section S9) with higher rotational barriers. Indeed, the ^{1}H NMR spectrum of **Ax**$^{2+}$ reveals the presence of six rotamers in the squaraine system (Figure S31-S33). No equilibration of the rotamers could be observed using EXSY experiments.

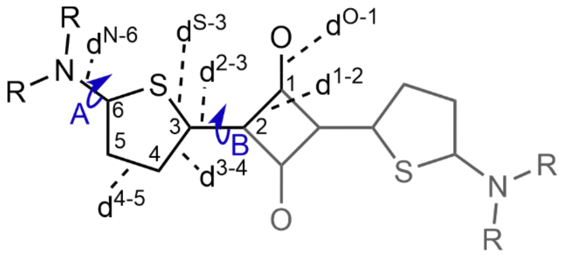

Figure S30: Annotation of discussed bond lengths and rotational barriers within the bis(aminothienyl)squaraine core. As the π-bond character varies through the oxidation states, only the single-bond scaffold of Sq is shown.

The rotational barriers of **Ax**^{0} and **Ax**$^{2+}$ were investigated by DFT (Tables S4 & S5), comparing the relative electronic energies of the rotational isomers and the rotational barriers that connect them. There seems to be no preferred rotamer in its free form as relative conformational stabilities are all below 4 kJ/mol independent of the charge of the axle (section S9). On the other hand, transition state optimization reveals a significant four-fold increase in the rotational barrier upon oxidation of the neutral (~ 47 kJ/mol) to the doubly charged species (~ 157 kJ/mol) rationalizing that no interconversion can be expected on the NMR timescale for the dication. The increasing double bond character of the C^2-C^3-bonds upon oxidation is revealed by shortening from 1.38 Å in **Ax**^{0} to 1.36 Å in **Ax**$^{\bullet+}$ to 1.34 Å in **Ax**$^{+2}$ and a similar trend is seen for the N-C^6-bonds (Table S4, Figure S30). The six rotamers of **Ax**$^{2+}$ are depicted in Figure S31.

*Table S4: Selected bond-lengths within the XRD structure (first column) and within the calculated most stable co-conformations (all further columns) of **Ax** and rotational barriers of **Ax**^{0} and **Ax**$^{2+}$ (DFT).*

state	**Ax**^{0} (XRD)	**Ax**$^{\bullet-}$	**Ax**^{0}	**Ax**$^{\bullet+}$	**Ax**$^{2+}$
**d^{1-2}** [Å]	1.467	1.461	1.459	1.471	1.490
**d^{2-3}** [Å]	1.381	1.399	1.381	1.364	1.340
**d^{3-4}** [Å]	1.402	1.382	1.394	1.416	1.444
**d^{4-5}** [Å]	1.376	1.412	1.382	1.360	1.340
**d^{S-3}** [Å]	1.757	1.756	1.752	1.751	1.747
**d^{N-6}** [Å]	1.346	1.356	1.331	1.309	1.290
**d^{O-1}** [Å]	1.229	1.229	1.222	1.212	1.198
rot. barrier A [kJ/mol]	-	-	41.2	-	159.5
rot. barrier B [kJ/mol]	-	-	53.6	-	155.2

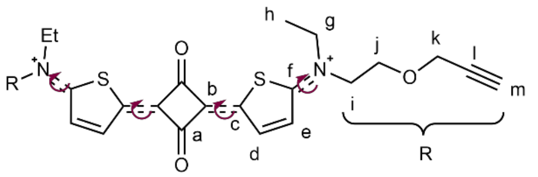

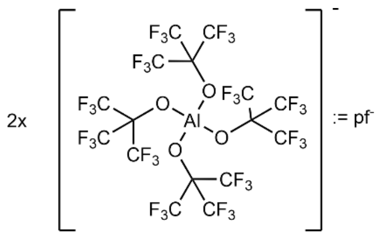

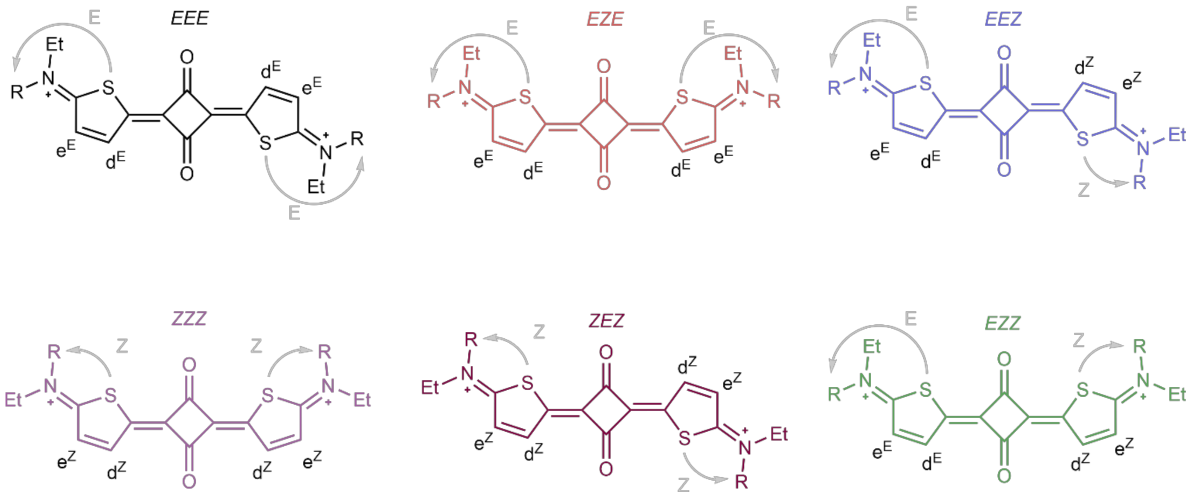

Figure S31: Rotamers of $\boldsymbol{Ax^{2+}}$ *resulting from E-Z isomerism of four double bonds.*

Sections of the ^{1}H NMR spectrum of $\mathbf{Ax^{2+}}$[pf^{-}]$_2$ in anhydrous CD_2Cl_2 are depicted in Figure S32 (full spectrum in section S1). The chemical shift changes upon oxidation can be seen in a stack of ^{1}H NMR spectra of $\mathbf{Ax^{0}}$ and $\mathbf{Ax^{2+}}$[pf^{-}]$_2$ in CD_2Cl_2 (Figure S33). The signals indexed with *E* are listed underlined, other species present in the solution are listed in grey.

^{1}**H NMR** (700 MHz, CD_2Cl_2, ppm): δ = 8.36-8.41 (3xd, *J* = 6.2 Hz, 2H, $\mathbf{d^{Z}}$), 8.25-8.29 (3xd, *J* = 6.2 Hz, 2H*x*2, $\mathbf{d^{E}}$), 7.77-7.80 (3xd, *J* = 6.2 Hz, 2H*x*2, $\mathbf{e^{E}}$), 7.65-7.68 (3xd, *J* = 6.2 Hz, 2H, $\mathbf{e^{Z}}$), 4.29 (t, *J* = 4.8 Hz, 4H*x*2, $\mathbf{i^{E}}$), 4.24 ($\mathbf{k^{Z}}$), 4.22-4.26 (d+t+q, 4H+4H+4H, $\mathbf{k^{Z}}$ & $\mathbf{i^{Z}}$ & $\mathbf{g^{Z}}$), 4.21 (d, *J* = 2.4 Hz, 4H*x*2, $\mathbf{k^{E}}$), 4.08-4.12 (q+t, 4H*x*2+4H, $\mathbf{g^{E}}$ & $\mathbf{j^{Z}}$), 4.00 (t, *J* = 4.8 Hz, 4H*x*2, $\mathbf{j^{E}}$), 3.83 (s, *H*O-C(CF_3)$_3$), 2.77 (s, H_3O^{+}), 2.59 (t, *J* = 2.4 Hz, 2H, $\mathbf{m^{z}}$), 2.58 (t, *J* = 2.4 Hz, 2H*x*2, $\mathbf{m^{E}}$), 1.65 (2xt, *J* = 7.2 Hz, 6H*x*2, $\mathbf{h^{E}}$), 1.60 (t, *J* = 7.2 Hz, 6H, $\mathbf{h^{Z}}$).

The assignment "3xd", for the group of signals between 8.36-8.41 ppm, for example, refers to a group of three independent doublets. As we cannot fully assign which doublet corresponds to which individual rotamer, we have grouped these signals according to the *E*/*Z* configuration of the adjacent N=C(thiopene) bond.

^{13}**C{**^{1}**H} NMR** (175 MHz, CD_2Cl_2, ppm): δ = 185.5, 185.5, 177.5, 158.4, 148.0, 147.9, 146.3, 146.2, 141.4, 141.3, 136.4, 136.4, 136.2, 136.1, 134.2, 134.1, 133.9, 133.9, 122.7 (q, $^{1}J_{CF}$ = 293.4 Hz, [pf]$^{-}$: *C*F_3), 121.3, 119.7, 78.0, 76.8, 76.7, 65.4, 65.2, 61.9, 59.3, 58.2, 58.1, 14.9, 12.7.

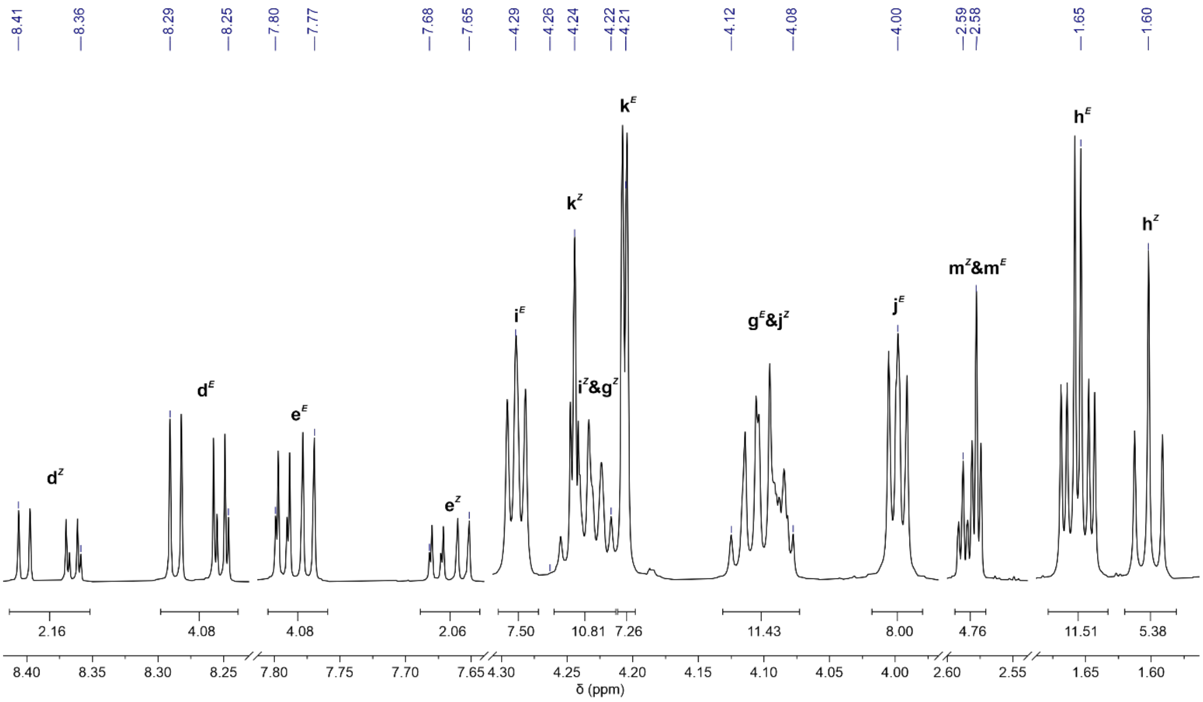

*Figure S32: Selected regions of the ^{1}H NMR spectrum of **Ax**$^{2+}$[pf$^-$]$_2$ in anhydrous CD_2Cl_2, 700 MHz, 298 K.*

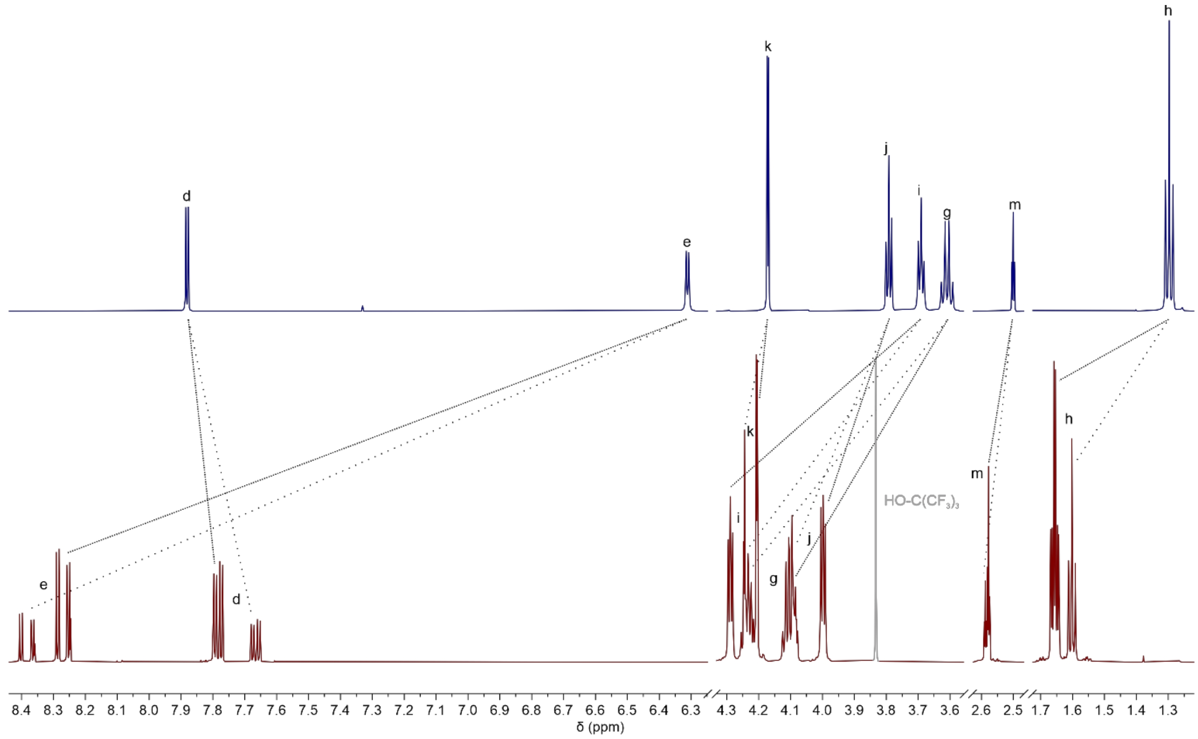

*Figure S33: Selected regions of the ^{1}H NMR spectra of **Ax**^{0} and **Ax**$^{2+}$[pf$^-$]$_2$ in anhydrous CD_2Cl_2, 700 MHz, 298 K.*

Section S7: Data on Oxidized Rotaxane $\textbf{Rot}^{2+}$

Rotaxane $\textbf{Rot}^{0}$ was oxidized in the same way as $\textbf{Ax}^{0}$ with $NOPF_6$ (10 equiv.) in anhydrous dichloromethane in the presence of Li[pf] (3.5 equiv.) to form $\textbf{Rot}^{2+}[pf^-]_2$. The orange-red solutions of $\textbf{Rot}^{2+}[pf^-]_2$ in anhydrous dichloromethane can be reduced successively back to the neutral rotaxane by tin powder (Figure S34).

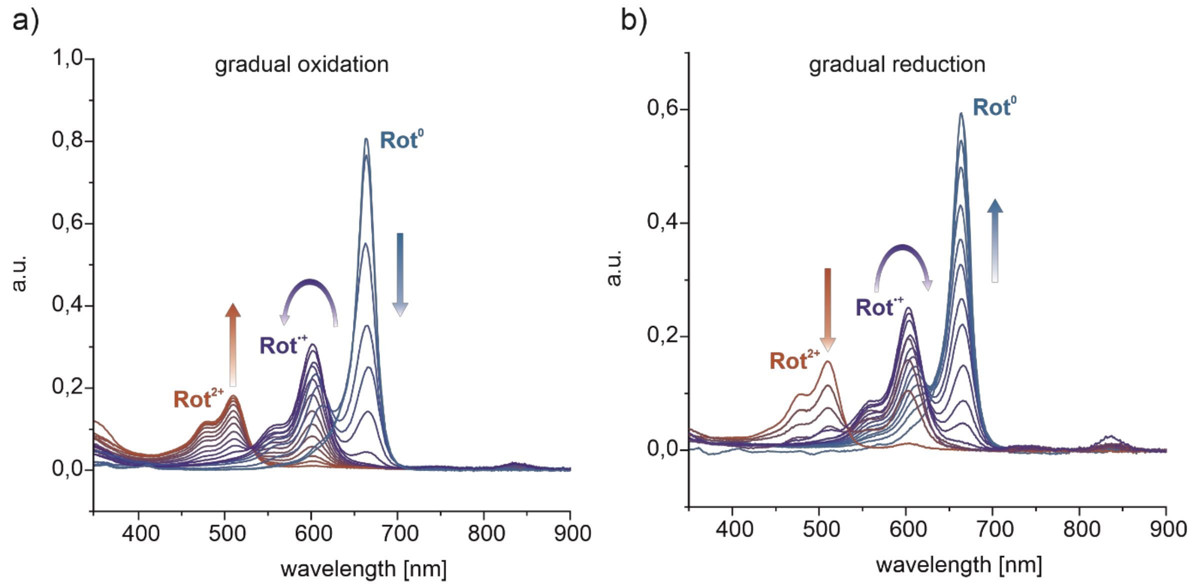

*Figure S34: Reversible gradual oxidation and reduction of **Rot**. a) UV-Vis titration of $\textbf{Rot}^{0}$ with NO[pf]. b) Reduction of the resulting solution of $\textbf{Rot[pf]}_2$ with tin powder.*

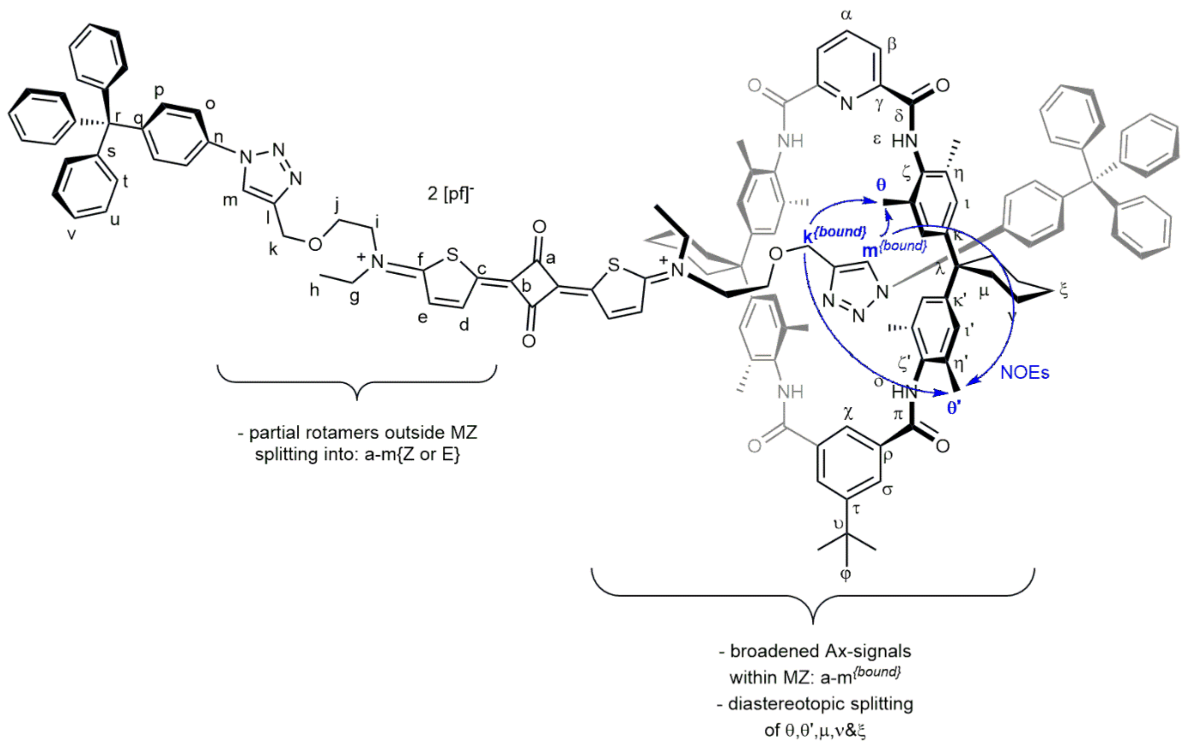

Figure S35: NOE-correlations indicative of the most stable co-conformation of $\textbf{Rot}^{2+}$.

The relevant signals were assigned by 2D NMR spectroscopy by NOESY (Figure S36), TOCSY (Figure S37), HSQC (Figure S38) and HMBC (Figure S39) experiments. Some NOE cross signals, indicative of the co-conformation of $\textbf{Rot}^{2+}$, are depicted in Figure S35.

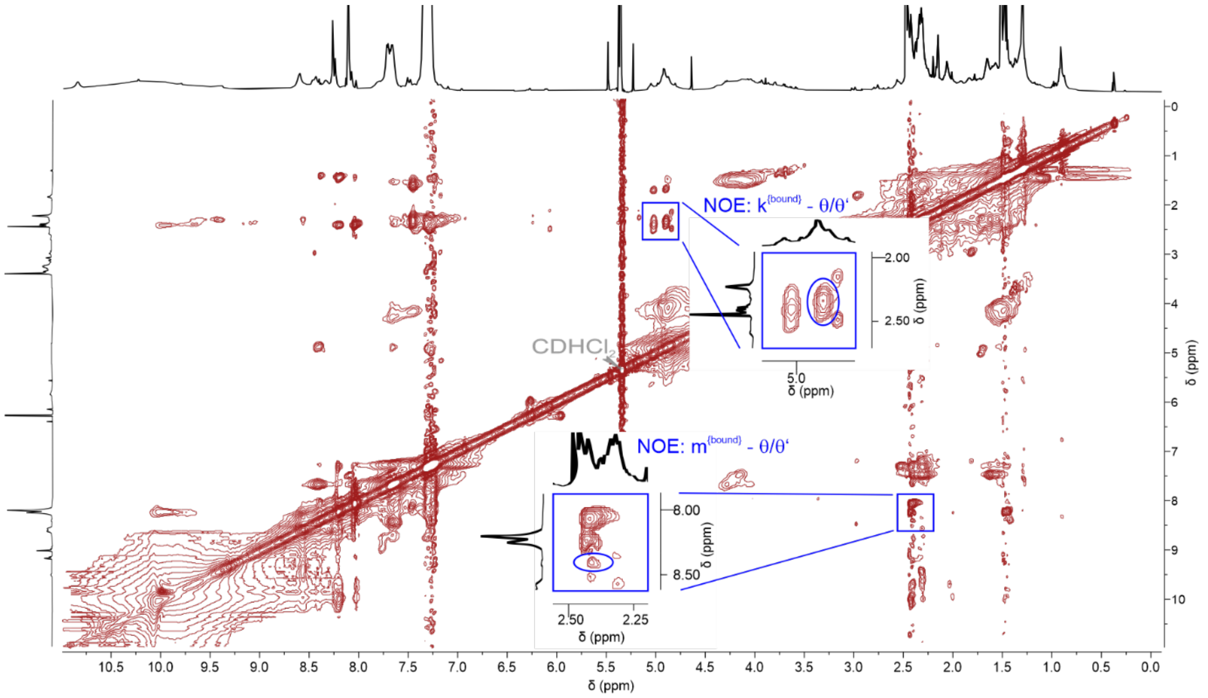

*Figure S36: ^{1}H,^{1}H NOESY spectrum of **Rot^{2+}**, 700 MHz, CD_2Cl_2, 298 K, 32 scans, 2048 x 448 complex points, mixing time 120 ms, recycle delay 1.5 s. Note that the NOE cross peak between the protons k$^{\{bound\}}$ and θ/θ' clearly evidences the proximity of the axle triazole and the methyl groups of the wheel, while the NOE between the protons m$^{\{bound\}}$ and θ/θ' is quite weak and should be seen as support but not clear evidence on its own.*

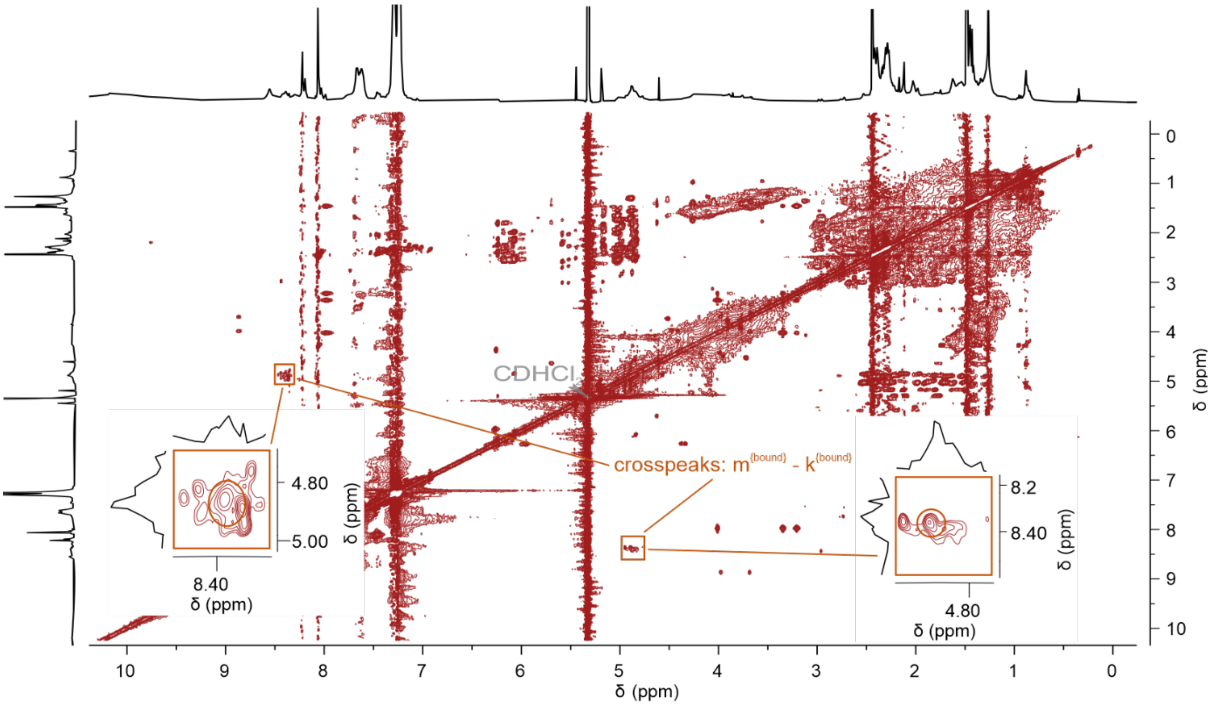

*Figure S37: ^{1}H,^{1}H TOCSY spectrum of **Rot^{2+}**, 700 MHz, CD_2Cl_2, 298 K, 4 scans, 2048 x 512 complex points, mixing time 80 ms, recycle delay 1.5 s.*

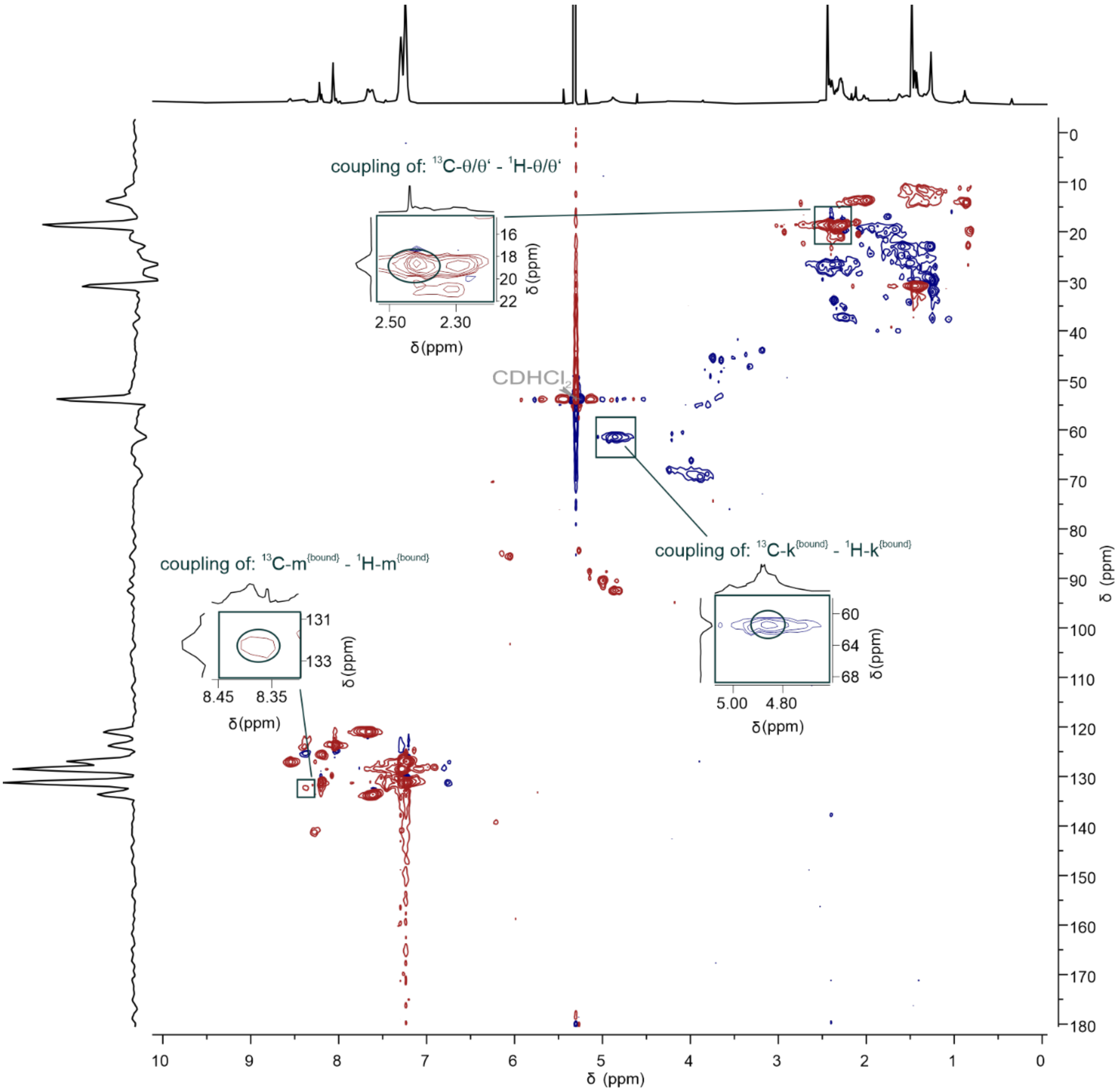

*Figure S38: ^{1}H,^{13}C HSQC spectrum of **Rot^{2+}**, 700 MHz, CD_2Cl_2, 298 K, 12 scans, 1024 x 400 complex points, recycle delay 1.5 s.*

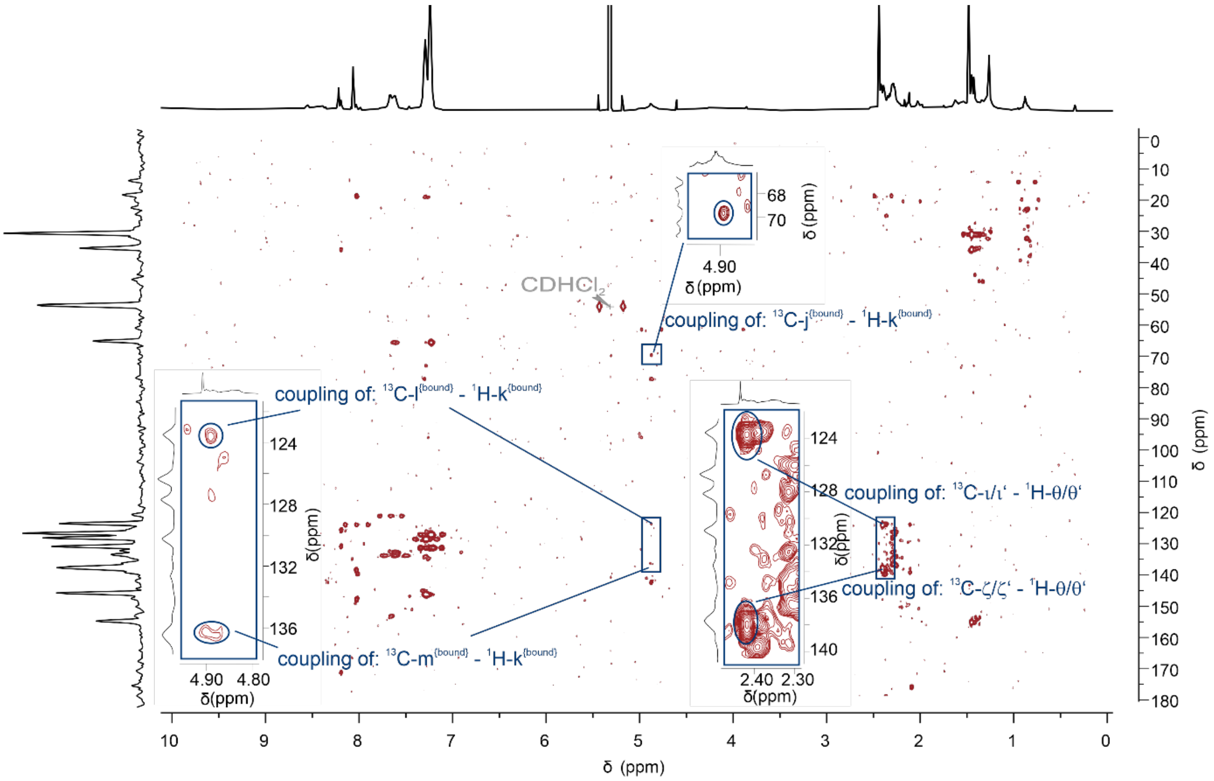

*Figure S39: $^{1}H,^{13}C$ HMBC spectrum of **Rot^{2+}**, 700 MHz, CD_2Cl_2, 298 K, 16 scans, 4096 x 512 complex points, recycle delay 1.5 s.*

Section S8: Low temperature ^{1}H NMR studies of Ax^{0} & Rot^{0}

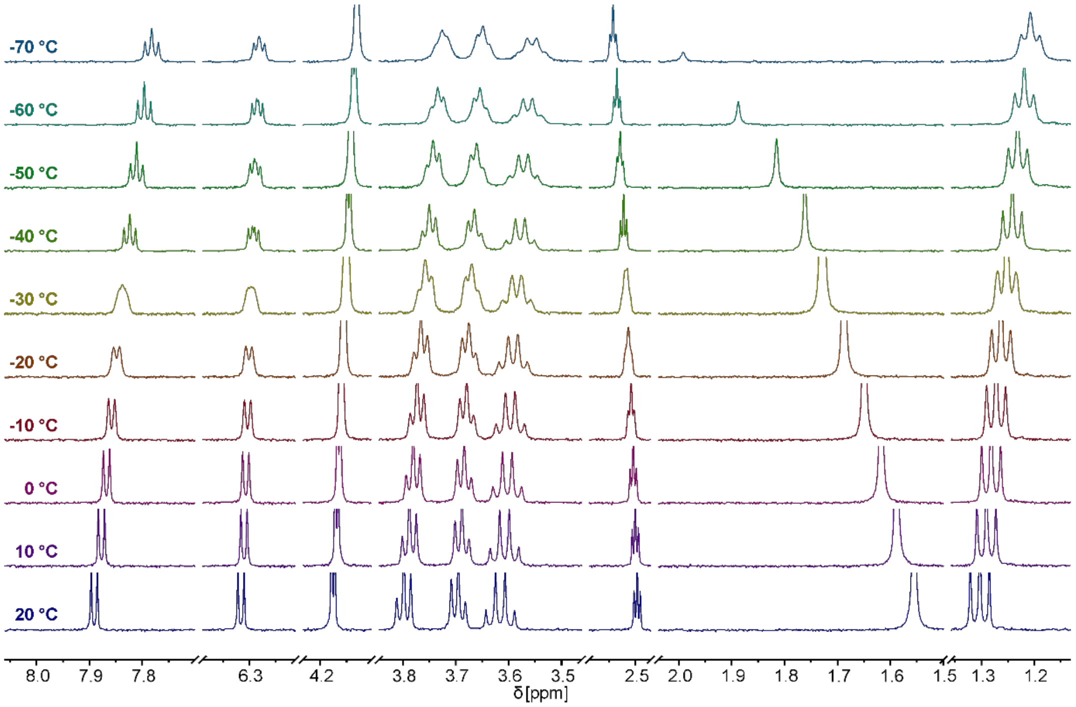

Figure S40: Stacked partial ^{1}H NMR spectra of ***Ax^{0}*** *at temperatures between −70 °C – 20 °C.*

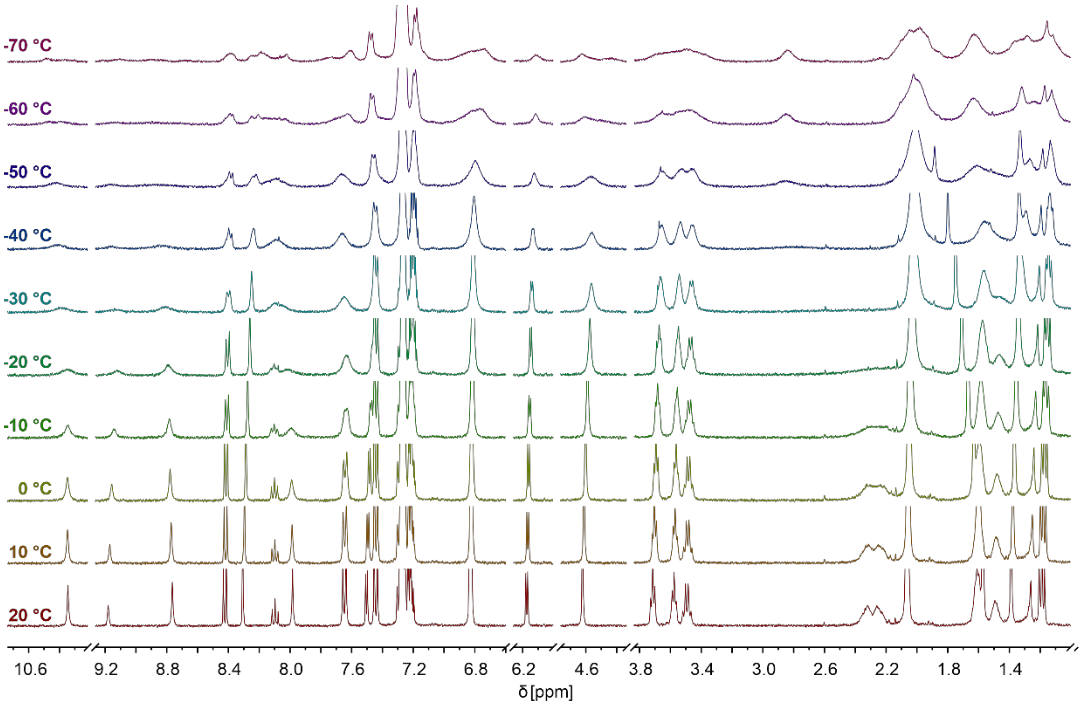

Figure S41: Stacked partial ^{1}H NMR spectra of ***Rot^{0}*** *at temperatures between −70 °C – 20 °C.*

Section S9: Quantum Chemical Calculations

*Table S5: Rotational barriers of **Ax** in two oxidation states around the N-C^6-bond (A) and the C^2-C^3-bond (B) using different density functional approximations. All values are given in kJ/mol.*

state	barrier	B3LYP-D3(BJ)	PBE0-D3(BJ)	BHLYP-D3(BJ)	wB97X-D3	average
**Ax^0**	A	43.2	44.2	39.8	37.4	41.2
	B	62.7	62.0	50.5	39.4	53.6
**Ax^{2+}**	A	156.6	159.8	160.7	161.0	159.5
	B	142.7	142.8	173.0	162.5	155.2

*Table S6: Calculated UV-Vis transitions of the squaraine core in four different oxidation states, calculated for **Ax**, and in two different oxidation states for **Rot**, * calculation was not feasible due to interfering "ghost states".*

state	functional	bright state [nm]	oscillator str.	other [nm]	oscillator str.
**$Ax^{\bullet-}$**	B3LYP	499.4 (S0->S2)	1.36	702.1 (S0->S1)	0.09
	PBE0	482.8 (S0->S2)	1.38	675.8 (S0->S1)	0.10
	BHLYP	438.6 (S0->S2)	1.30	598.9 (S0->S1)	0.13
	wB97X	441.7 (S0->S2)	1.22	624.1 (S0->S1)	0.19
**Ax^0**	B3LYP	582.8 (S0->S1)	1.71		
	PBE0	577.3 (S0->S1)	1.75		
	BHLYP	567.3 (S0->S1)	1.86		
	wB97X	596.0 (S0->S1)	1.76		
**$Ax^{\bullet+}$**	B3LYP	582.8 (S0->S3)	1.67	827.8 (S0->S1)	0.0008
	PBE0	577.4 (S0->S3)	1.72	794.8 (S0->S1)	0.00003
	BHLYP	562.0 (S0->S2)	1.80	668.1 (S0->S1)	0.0004
	wB97X	562.0 (S0->S2)	1.72	695.6 (S0->S1)	0.07
**Ax^{2+}**	B3LYP	537.3 (S0->S2)	1.12		
	PBE0	520.0 (S0->S2)	1.39		
	BHLYP	443.4 (S0->S2)	1.95		
	wB97X	445.8 (S0->S2)	1.86		
**Rot^0**	B3LYP	593.1 (S0->S1)	1.24		
	PBE0	586.8 (S0->S1)	1.27		
	BHLYP	572.5 (S0->S1)	1.35		
	wB97X-D3	599.8 (S0->S1)	1.30		
**Rot^{2+}**	B3LYP	*	*		
	PBE0	*	*		
	BHLYP	463.7 (S0->S5)	1.54		
	wB97X-D3	471.2 (S0->S2)	1.55		

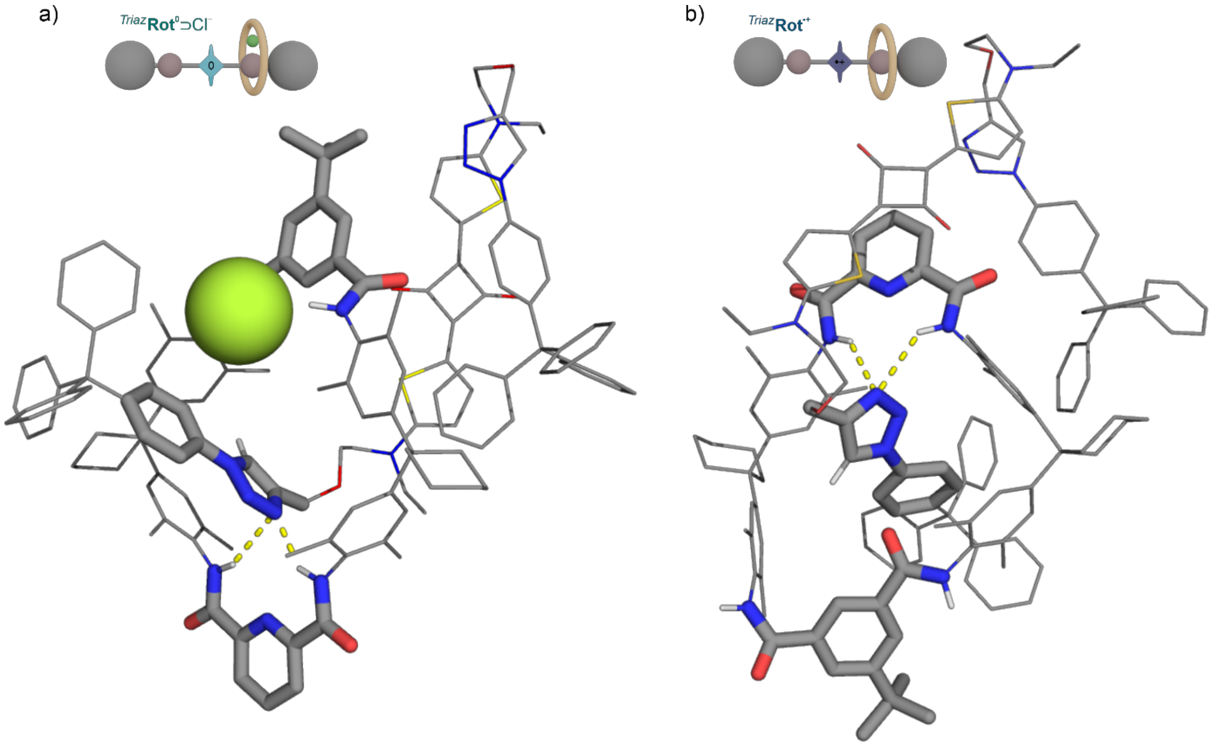

*Figure S42: Binding motifs between the triazol station and **TLM** in a) ^{Triaz}**Rot^0**⊃Cl$^-$ and b) ^{Triaz}**Rot$^{\bullet+}$** in the calculated most stable coconformations using CREST.*

*Table S7: Relative stabilities ($\Delta E = E(^{Triaz}$**Rot**$) - E(^{Sq}$**Rot**$)$) of two co-conformations of **Rot** in four different oxidation states using different density functional approximations. All values are given in kJ/mol.*

state	B3-LYP-D3(BJ)	PBE0-D3(BJ)	BHLYP-D3(BJ)	wB97X-D3	average
^{Sq}**Rot**$^{\bullet-}$	66.86	74.98	78.87	70.17	72.72
^{Sq}**Rot**^{0}	20.54	28.53	29.83	38.83	29.41
^{Triaz}**Rot**$^{\bullet+}$	-7.36	-3.64	0.29	-3.39	-3.51
^{Triaz}**Rot**$^{2+}$	-18.54	-16.65	-15.10	-19.96	-17.57

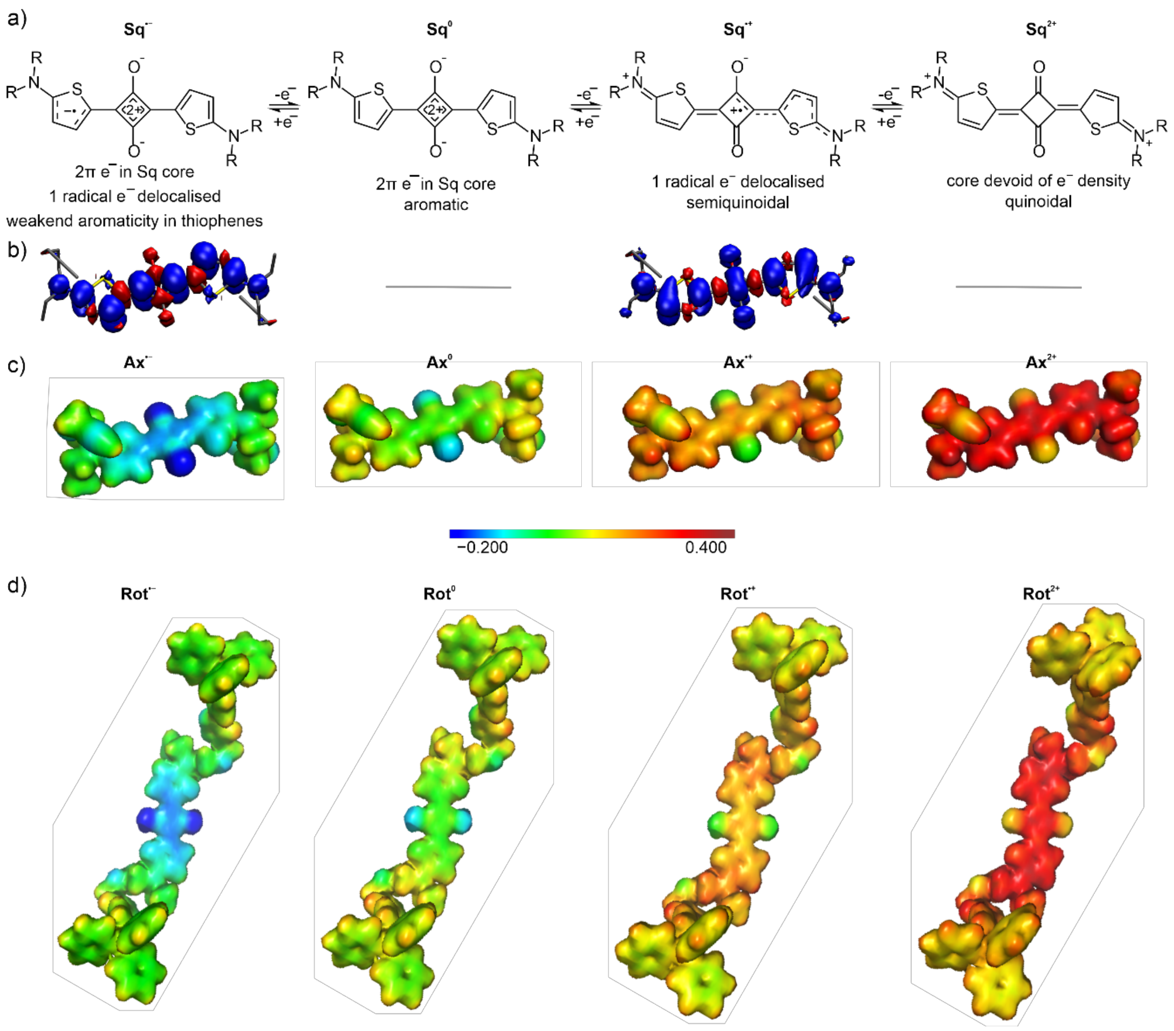

Figure S43: a) Resonance structures of the squaraine core in four different oxidation states. Only one relevant resonance structure is depicted for each state. b) Spin-density plots of the radical species **Ax**$^{\bullet-}$ *and* **Ax**$^{\bullet+}$*, isovalue: 0.02 a.u.. c) Electrostatic potential surface maps of* **Ax** *in four different oxidation states. d) Electrostatic potential surface maps of the stoppered axle as present in* **Rot** *in four different oxidation states. For better visibility, the wheel was removed from the ESPs of* **Rot** *to display the charge distribution of the axle component.*

Section S10: Stability measurements of **Ax⁰**, **Rot⁰**, **Ax²⁺** and **Rot²⁺**

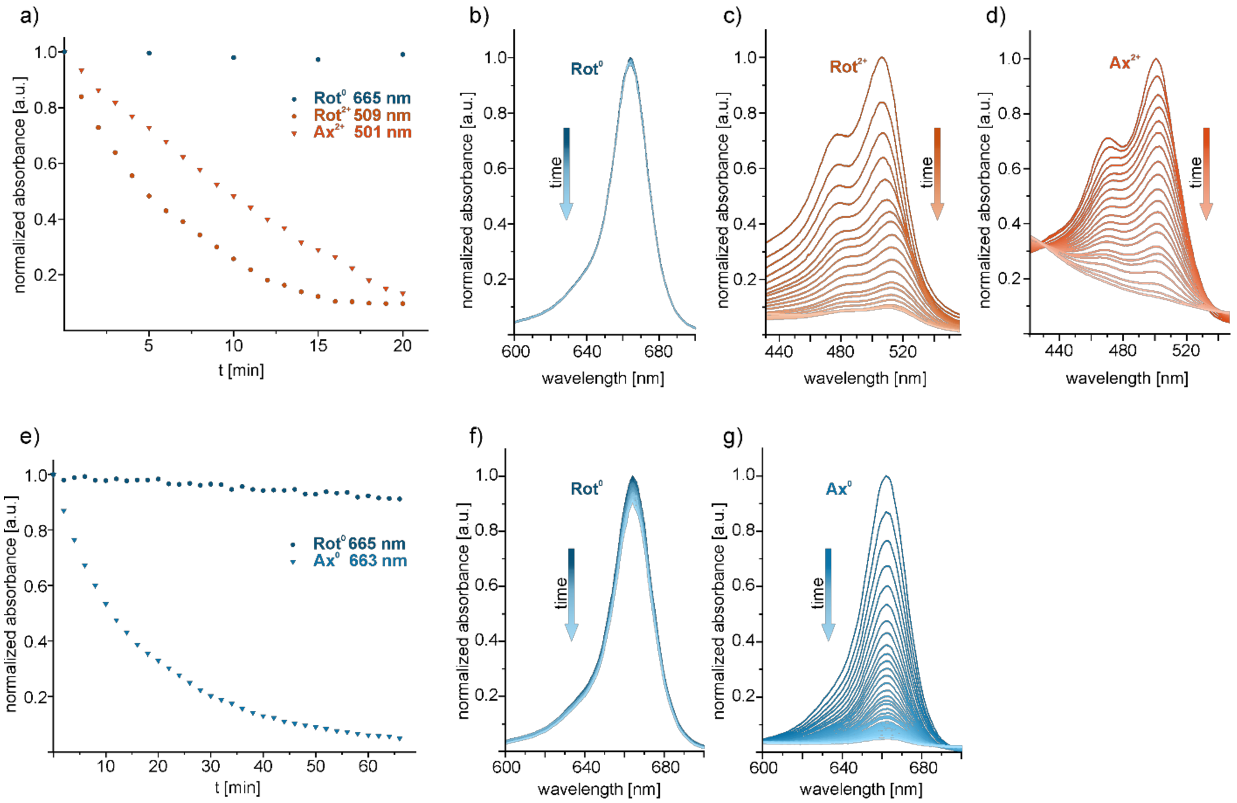

*Figure S44: Bleaching study of protected and unprotected squaraine species by nucleophiles. a) Bleaching of **Ax²⁺**, **Rot²⁺** and **Rot⁰** in a saturated solution of $NMe_4OH \cdot 3H_2O$ in CH_2Cl_2. b) Stacked UV-Vis spectra of **Rot⁰**. c) Stacked UV-Vis spectra of **Rot²⁺**. d) Stacked UV-Vis spectra of **Ax²⁺**. e) Bleaching of **Ax⁰** and **Rot⁰** in a saturated solution of KO^tBu in CH_2Cl_2 95% and ^tBuOH 5%. f) Stacked UV-Vis spectra of **Rot⁰**. g) Stacked UV-Vis spectra of **Ax⁰**.*

To conduct the bleaching experiments of **Ax²⁺**, **Rot²⁺** and **Rot⁰**, solutions of the respective squaraine in dichloromethane were saturated with tetramethylammonium hydroxide trihydrate and UV-Vis spectra were recorded every minute in the case of **Ax²⁺** and **Rot²⁺**, and every five minutes in the case of **Rot⁰**. The normalised relative absorbance at the corresponding absorption maximum of the compounds was plotted against the reaction time.

The bleaching experiments of **Ax⁰** and **Rot⁰** required a higher concentration of nucleophile and hydroxide was substituted with the more sterically demanding *ᵗ*butoxide. To increase the solubility of potassium *ᵗ*butoxide, a mixture of 95% dichloromethane and 5% of *ᵗ*BuOH was used as solvent. To the solutions of the respective squaraine, 5 mg of KOᵗBu were added and UV-Vis spectra were recorded every two minutes. The normalised relative absorbance at the corresponding absorption maximum of the compounds was plotted against the reaction time.

References

1. Hübler, C. SupraFit – An Open Source Qt Based Fitting Application to Determine Stability Constants from Titration Experiments**. *Chemistry - Methods* **2,** e202200006; 10.1002/cmtd.202200006 (2022).
2. Thordarson, P. Determining association constants from titration experiments in supramolecular chemistry. *Chem. Soc. Rev.* **40,** 1305–1323; 10.1039/C0CS00062K (2011).
3. Brynn Hibbert, D. & Thordarson, P. The death of the Job plot, transparency, open science and online tools, uncertainty estimation methods and other developments in supramolecular chemistry data analysis. *Chem. Commun.* **52,** 12792–12805; 10.1039/C6CC03888C (2016).
4. Supramolecular.org - Binding Constant Calculators | Supramolecular. Available at http://supramolecular.org/ (2024).
5. *CrysAlisPro* (Rigaku Oxford Diffraction Ltd., Yarnton (Oxfordshire, England), 2015).
6. Sheldrick, G. M. SHELXT - integrated space-group and crystal-structure determination. *Acta Crystallogr. A* **71,** 3–8; 10.1107/S2053273314026370 (2015).
7. Sheldrick, G. M. Crystal structure refinement with SHELXL. *Acta Crystallogr. C* **71,** 3–8; 10.1107/S2053229614024218 (2015).
8. Allen, F. H. & Bruno, I. J. Bond lengths in organic and metal-organic compounds revisited: X-H bond lengths from neutron diffraction data. *Acta Crystallogr. B* **66,** 380–386; 10.1107/S0108768110012048 (2010).
9. Macrae, C. F. *et al.* Mercury 4.0: from visualization to analysis, design and prediction. *J. Appl. Crystallogr.* **53,** 226–235; 10.1107/S1600576719014092 (2020).
