# Supplementary material for: Switchable protection and exposure of a sensitive squaraine dye within a redox active rotaxane
Source: Commun Chem. 2024 Oct 4;7:229. doi: 10.1038/s42004-024-01312-1 (PMC11452610; doi:10.1038/s42004-024-01312-1)
Supplement: Supplementary file 2 — Description of Additional Supplementary Files [file 42004_2024_1312_MOESM2_ESM.pdf]

Description of Additional Supplementary Files

File name: Supplementary Data 1

Description: XRD Ax

File name: Supplementary Data 2

Description: XRD psRot

File name: Supplementary Data 3

Description: Checkcif Ax

File name: Supplementary Data 4

Description: Checkcif psRot

File name: Supplementary Data 5

Description: Calculated structures
