# Supplementary material for: Switchable protection and exposure of a sensitive squaraine dye within a redox active rotaxane
Source: Commun Chem. 2024 Oct 4;7:229. doi: 10.1038/s42004-024-01312-1 (PMC11452610; doi:10.1038/s42004-024-01312-1)

checkCIF/PLATON report

Structure factors have been supplied for datablock(s) Ax

THIS REPORT IS FOR GUIDANCE ONLY. IF USED AS PART OF A REVIEW PROCEDURE FOR PUBLICATION, IT SHOULD NOT REPLACE THE EXPERTISE OF AN EXPERIENCED CRYSTALLOGRAPHIC REFEREE.

No syntax errors found.   CIF dictionary   Interpreting this report

Datablock: Ax

```
Bond precision:   C-C = 0.0044 A          Wavelength=1.54184

Cell:             a=9.6863(3)     b=10.8682(3)       c=11.4392(3)
                  alpha=90        beta=94.582(2)     gamma=90
Temperature:      120 K

                 Calculated                   Reported
Volume           1200.39(6)                   1200.39(6)
Space group      P 21                         P 21
Hall group       P 2yb                        P 2yb
Moiety formula   C26 H28 N2 O4 S2             C26 H28 N2 O4 S2
Sum formula      C26 H28 N2 O4 S2             C26 H28 N2 O4 S2
Mr               496.62                       496.62
Dx,g cm-3        1.374                        1.374
Z                2                            2
Mu (mm-1)        2.309                        2.309
F000             524.0                        524.0
F000'            526.73
h,k,lmax         12,13,14                     11,13,14
Nref             4825[ 2546]                  3414
Tmin,Tmax        0.758,0.871                  0.811,1.000
Tmin'            0.741

Correction method= # Reported T Limits: Tmin=0.811 Tmax=1.000
AbsCorr = GAUSSIAN

Data completeness= 1.34/0.71       Theta(max)= 73.436

R(reflections)= 0.0335( 3270)                          wR2(reflections)=
                                                       0.0882( 3414)

S = 1.030                   Npar= 308
```

```
The following ALERTS were generated. Each ALERT has the format
       test-name_ALERT_alert-type_alert-level.
Click on the hyperlinks for more details of the test.
```

Alert level B

```
PLAT915_ALERT_3_B No Flack x Check Done: Low Friedel Pair Coverage         42 %
```

Author Response: Eventhough Ax is symmetric and achiral, the structure can't be solved in a centrosymmetric space group. Thus it was solved and refined as a racemic twin (TWIN/BASF) in P21.

Alert level C

```
PLAT340_ALERT_3_C Low Bond Precision on  C-C Bonds ...............     0.0044 Ang.
PLAT911_ALERT_3_C Missing FCF Refl Between Thmin & STh/L=    0.600          2 Report
               -2 12  5,  -2 11  7,
```

Alert level G

```
PLAT033_ALERT_4_G Flack x Value Deviates > 3.0 * sigma from Zero .      0.296 Note
PLAT883_ALERT_1_G No Info/Value for _atom_sites_solution_primary .     Please Do !
PLAT899_ALERT_4_G SHELXL2018 is Deprecated and Succeeded by SHELXL     2019/3 Note
PLAT912_ALERT_4_G Missing # of FCF Reflections Above STh/L=  0.600         88 Note
PLAT933_ALERT_2_G Number of HKL-OMIT Records in Embedded .res File          2 Note
                2 -1  0,   1  1  2,
PLAT941_ALERT_3_G Average HKL Measurement Multiplicity ..........         1.9 Low
PLAT978_ALERT_2_G Number C-C Bonds with Positive Residual Density.          8 Info
```

```
   0 ALERT level A = Most likely a serious problem - resolve or explain
   1 ALERT level B = A potentially serious problem, consider carefully
   2 ALERT level C = Check. Ensure it is not caused by an omission or oversight
   7 ALERT level G = General information/check it is not something unexpected

   1 ALERT type 1 CIF construction/syntax error, inconsistent or missing data
   2 ALERT type 2 Indicator that the structure model may be wrong or deficient
   4 ALERT type 3 Indicator that the structure quality may be low
   3 ALERT type 4 Improvement, methodology, query or suggestion
   0 ALERT type 5 Informative message, check
```

It is advisable to attempt to resolve as many as possible of the alerts in all categories. Often the minor alerts point to easily fixed oversights, errors and omissions in your CIF or refinement strategy, so attention to these fine details can be worthwhile. In order to resolve some of the more serious problems it may be necessary to carry out additional measurements or structure refinements. However, the purpose of your study may justify the reported deviations and the more serious of these should normally be commented upon in the discussion or experimental section of a paper or in the "special_details" fields of the CIF. checkCIF was carefully designed to identify outliers and unusual parameters, but every test has its limitations and alerts that are not important in a particular case may appear. Conversely, the absence of alerts does not guarantee there are no aspects of the results needing attention. It is up to the individual to critically assess their own results and, if necessary, seek expert advice.

Publication of your CIF in IUCr journals

A basic structural check has been run on your CIF. These basic checks will be run on all CIFs submitted for publication in IUCr journals (*Acta Crystallographica*, *Journal of Applied Crystallography*, *Journal of Synchrotron Radiation*); however, if you intend to submit to *Acta Crystallographica Section C* or *E* or *IUCrData*, you should make sure that full publication checks are run on the final version of your CIF prior to submission.

Publication of your CIF in other journals

Please refer to the *Notes for Authors* of the relevant journal for any special instructions relating to CIF submission.

PLATON version of 29/11/2023; check.def file version of 14/09/2023

Datablock Ax - ellipsoid plot

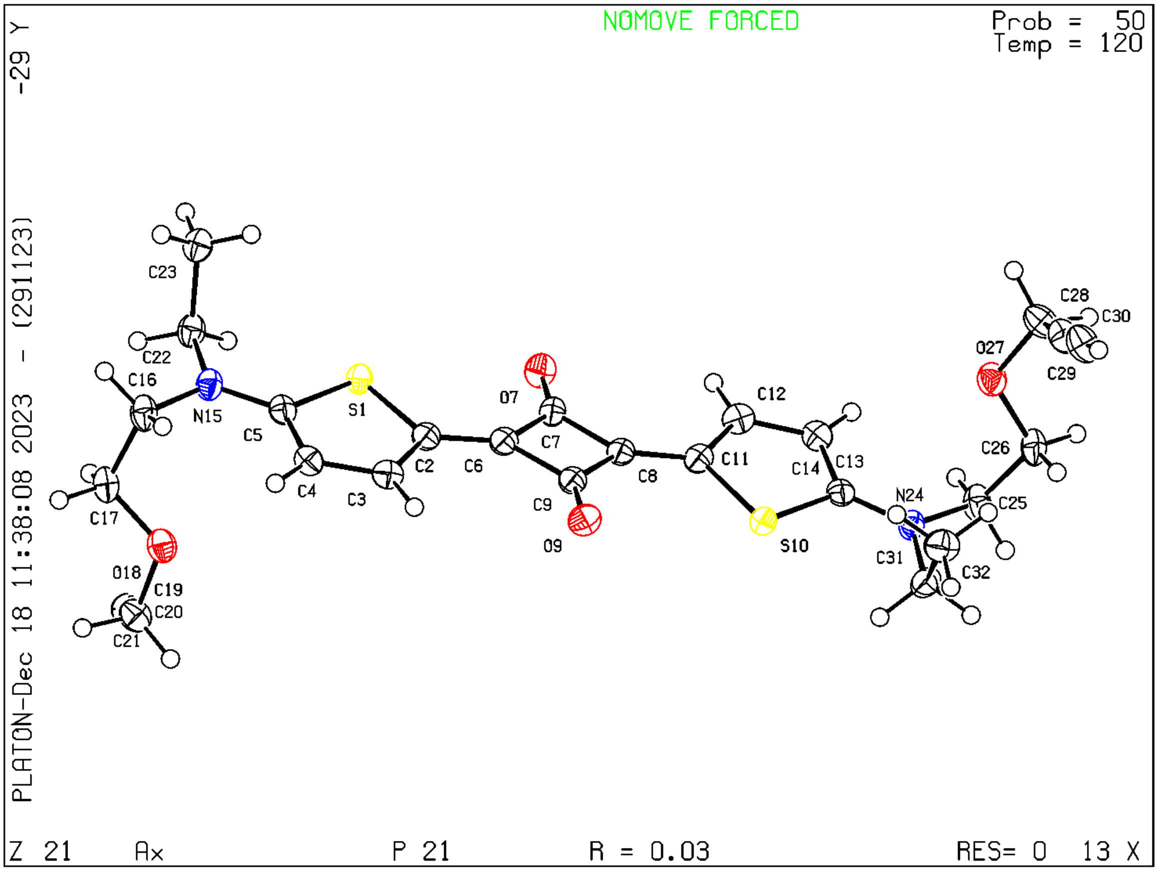

Supplement: Supplementary file 5 — Supplementary Data 3 [file 42004_2024_1312_MOESM5_ESM.pdf]
