# Supplementary material for: Switchable protection and exposure of a sensitive squaraine dye within a redox active rotaxane
Source: Commun Chem. 2024 Oct 4;7:229. doi: 10.1038/s42004-024-01312-1 (PMC11452610; doi:10.1038/s42004-024-01312-1)

checkCIF/PLATON report

Structure factors have been supplied for datablock(s) Pseu

THIS REPORT IS FOR GUIDANCE ONLY. IF USED AS PART OF A REVIEW PROCEDURE FOR PUBLICATION, IT SHOULD NOT REPLACE THE EXPERTISE OF AN EXPERIENCED CRYSTALLOGRAPHIC REFEREE.

No syntax errors found. CIF dictionary Interpreting this report

Datablock: psRot

Bond precision:	C-C = 0.0072 A		Wavelength=1.54184
Cell:	a=16.5888(4)	b=15.0832(4)	c=16.7501(3)
	alpha=90	beta=98.607(2)	gamma=90
Temperature:	120 K		

	Calculated	Reported
Volume	4143.88(17)	4143.88(17)
Space group	P 21/n	P 21/n
Hall group	-P 2yn	-P 2yn
Moiety formula	C63 H71 N5 O4, C26 H28 N2 O4 S2, C H2 Cl2	C63 H71 N5 O4, C26 H28 N2 O4 S2, C H2 Cl2
Sum formula	C90 H101 Cl2 N7 O8 S2	C90 H101 Cl2 N7 O8 S2
Mr	1543.80	1543.79
Dx,g cm-3	1.237	1.237
Z	2	2
Mu (mm-1)	1.652	1.652
F000	1640.0	1640.0
F000'	1647.05	
h,k,lmax	20,18,20	20,18,20
Nref	8318	8047
Tmin,Tmax	0.743,0.820	0.568,0.738
Tmin'	0.651	

Correction method= # Reported T Limits: Tmin=0.568 Tmax=0.738
AbsCorr = ANALYTICAL

Data completeness= 0.967 Theta(max)= 73.214

R(reflections)= 0.1006(6722) wR2(reflections)= 0.2596(8047)

S = 1.084 Npar= 795

The following ALERTS were generated. Each ALERT has the format
test-name_ALERT_alert-type_alert-level.
Click on the hyperlinks for more details of the test.

Alert level C

```
PLAT084_ALERT_3_C High wR2 Value (i.e. > 0.25) ...................       0.26 Report
PLAT094_ALERT_2_C Ratio of Maximum / Minimum Residual Density ....       2.15 Report
PLAT215_ALERT_3_C Disordered N1B       has ADP max/min Ratio .....        4.0 Note
PLAT234_ALERT_4_C Large Hirshfeld Difference O47      --C48B     .       0.17 Ang.
PLAT250_ALERT_2_C Large U3/U1 Ratio for Average U(i,j) Tensor ....        2.3 Note
PLAT334_ALERT_2_C Small <C-C> Benzene Dist.   C1       -C6       .       1.37 Ang.
PLAT340_ALERT_3_C Low Bond Precision on  C-C Bonds ..............    0.00721 Ang.
PLAT906_ALERT_3_C Large K Value in the Analysis of Variance ......     14.317 Check
PLAT906_ALERT_3_C Large K Value in the Analysis of Variance ......      3.274 Check
PLAT906_ALERT_3_C Large K Value in the Analysis of Variance ......      2.021 Check
PLAT911_ALERT_3_C Missing FCF Refl Between Thmin & STh/L=    0.600         34 Report
               11 15  0, -11 15  1,  -9  0  1,  12 14  1,  -9 16  2,  12 14  2,
               -9 16  3,   2  0  4,  10  9  4, -19  0  5, -19  1  5,  10  9  5,
              -18  0  6, -18  1  6,   1  6  6,   1  7  6,   2  8  6, -17  0  7,
              -17  1  7,   1  6  7,   2  7  7,   3  7  7, -15  1  8,   2  6  8,
              -14  9 12, -12 10 13, -11 10 14,  -9 10 15,   5  0 15,   5 10 15,
                6  9 15,  -7 10 16,   4  9 16,   1  0 19,
```

Alert level G

```
PLAT002_ALERT_2_G Number of Distance or Angle Restraints on AtSite         51 Note
PLAT003_ALERT_2_G Number of Uiso or Uij Restrained non-H Atoms ...         39 Report
PLAT083_ALERT_2_G SHELXL Second Parameter in WGHT  Unusually Large       7.81 Why ?
PLAT172_ALERT_4_G The CIF-Embedded .res File Contains DFIX Records          4 Report
PLAT176_ALERT_4_G The CIF-Embedded .res File Contains SADI Records         15 Report
PLAT178_ALERT_4_G The CIF-Embedded .res File Contains SIMU Records          8 Report
PLAT188_ALERT_3_G A Non-default SIMU Restraint Value has been used     0.0100 Report
PLAT188_ALERT_3_G A Non-default SIMU Restraint Value has been used     0.0100 Report
PLAT188_ALERT_3_G A Non-default SIMU Restraint Value has been used     0.0100 Report
PLAT188_ALERT_3_G A Non-default SIMU Restraint Value has been used     0.0100 Report
PLAT188_ALERT_3_G A Non-default SIMU Restraint Value has been used     0.0100 Report
PLAT188_ALERT_3_G A Non-default SIMU Restraint Value has been used     0.0100 Report
PLAT188_ALERT_3_G A Non-default SIMU Restraint Value has been used     0.0100 Report
PLAT188_ALERT_3_G A Non-default SIMU Restraint Value has been used     0.0100 Report
PLAT300_ALERT_4_G Atom Site Occupancy of O7         Constrained at        0.5 Check
PLAT300_ALERT_4_G Atom Site Occupancy of O7B        Constrained at        0.5 Check
PLAT300_ALERT_4_G Atom Site Occupancy of O21        Constrained at        0.5 Check
PLAT300_ALERT_4_G Atom Site Occupancy of O21B       Constrained at        0.5 Check
PLAT300_ALERT_4_G Atom Site Occupancy of N1B        Constrained at        0.5 Check
PLAT300_ALERT_4_G Atom Site Occupancy of N8         Constrained at        0.5 Check
PLAT300_ALERT_4_G Atom Site Occupancy of N8B        Constrained at        0.5 Check
PLAT300_ALERT_4_G Atom Site Occupancy of C1         Constrained at        0.5 Check
PLAT300_ALERT_4_G Atom Site Occupancy of C2         Constrained at        0.5 Check
PLAT300_ALERT_4_G Atom Site Occupancy of C2B        Constrained at        0.5 Check
PLAT300_ALERT_4_G Atom Site Occupancy of C3         Constrained at        0.5 Check
PLAT300_ALERT_4_G Atom Site Occupancy of C3B        Constrained at        0.5 Check
PLAT300_ALERT_4_G Atom Site Occupancy of C4         Constrained at        0.5 Check
PLAT300_ALERT_4_G Atom Site Occupancy of C4B        Constrained at        0.5 Check
PLAT300_ALERT_4_G Atom Site Occupancy of C5         Constrained at        0.5 Check
PLAT300_ALERT_4_G Atom Site Occupancy of C5B        Constrained at        0.5 Check
```

```
PLAT300_ALERT_4_G Atom Site Occupancy of C6         Constrained at        0.5 Check
PLAT300_ALERT_4_G Atom Site Occupancy of C6B        Constrained at        0.5 Check
PLAT300_ALERT_4_G Atom Site Occupancy of C7         Constrained at        0.5 Check
PLAT300_ALERT_4_G Atom Site Occupancy of C7B        Constrained at        0.5 Check
PLAT300_ALERT_4_G Atom Site Occupancy of C9         Constrained at        0.5 Check
PLAT300_ALERT_4_G Atom Site Occupancy of C9B        Constrained at        0.5 Check
PLAT300_ALERT_4_G Atom Site Occupancy of C10        Constrained at        0.5 Check
PLAT300_ALERT_4_G Atom Site Occupancy of C10B       Constrained at        0.5 Check
PLAT300_ALERT_4_G Atom Site Occupancy of C11        Constrained at        0.5 Check
PLAT300_ALERT_4_G Atom Site Occupancy of C11B       Constrained at        0.5 Check
PLAT300_ALERT_4_G Atom Site Occupancy of C12        Constrained at        0.5 Check
PLAT300_ALERT_4_G Atom Site Occupancy of C12B       Constrained at        0.5 Check
PLAT300_ALERT_4_G Atom Site Occupancy of C13        Constrained at        0.5 Check
PLAT300_ALERT_4_G Atom Site Occupancy of C13B       Constrained at        0.5 Check
PLAT300_ALERT_4_G Atom Site Occupancy of C14        Constrained at        0.5 Check
PLAT300_ALERT_4_G Atom Site Occupancy of C14B       Constrained at        0.5 Check
PLAT300_ALERT_4_G Atom Site Occupancy of C21        Constrained at        0.5 Check
PLAT300_ALERT_4_G Atom Site Occupancy of C21B       Constrained at        0.5 Check
PLAT300_ALERT_4_G Atom Site Occupancy of C23        Constrained at        0.5 Check
PLAT300_ALERT_4_G Atom Site Occupancy of C23B       Constrained at        0.5 Check
PLAT300_ALERT_4_G Atom Site Occupancy of C24        Constrained at        0.5 Check
PLAT300_ALERT_4_G Atom Site Occupancy of C24B       Constrained at        0.5 Check
PLAT300_ALERT_4_G Atom Site Occupancy of C25        Constrained at        0.5 Check
PLAT300_ALERT_4_G Atom Site Occupancy of C25B       Constrained at        0.5 Check
PLAT300_ALERT_4_G Atom Site Occupancy of C26        Constrained at        0.5 Check
PLAT300_ALERT_4_G Atom Site Occupancy of C26B       Constrained at        0.5 Check
PLAT300_ALERT_4_G Atom Site Occupancy of C27        Constrained at        0.5 Check
PLAT300_ALERT_4_G Atom Site Occupancy of C27B       Constrained at        0.5 Check
PLAT300_ALERT_4_G Atom Site Occupancy of C28        Constrained at        0.5 Check
PLAT300_ALERT_4_G Atom Site Occupancy of C28B       Constrained at        0.5 Check
PLAT300_ALERT_4_G Atom Site Occupancy of C29        Constrained at        0.5 Check
PLAT300_ALERT_4_G Atom Site Occupancy of C29B       Constrained at        0.5 Check
PLAT300_ALERT_4_G Atom Site Occupancy of C30        Constrained at        0.5 Check
PLAT300_ALERT_4_G Atom Site Occupancy of C30B       Constrained at        0.5 Check
PLAT300_ALERT_4_G Atom Site Occupancy of C31        Constrained at        0.5 Check
PLAT300_ALERT_4_G Atom Site Occupancy of C31B       Constrained at        0.5 Check
PLAT300_ALERT_4_G Atom Site Occupancy of C32        Constrained at        0.5 Check
PLAT300_ALERT_4_G Atom Site Occupancy of C32B       Constrained at        0.5 Check
PLAT300_ALERT_4_G Atom Site Occupancy of C33        Constrained at        0.5 Check
PLAT300_ALERT_4_G Atom Site Occupancy of C34        Constrained at        0.5 Check
PLAT300_ALERT_4_G Atom Site Occupancy of C35        Constrained at        0.5 Check
PLAT300_ALERT_4_G Atom Site Occupancy of C36        Constrained at        0.5 Check
PLAT300_ALERT_4_G Atom Site Occupancy of H1         Constrained at        0.5 Check
PLAT300_ALERT_4_G Atom Site Occupancy of H3         Constrained at        0.5 Check
PLAT300_ALERT_4_G Atom Site Occupancy of H3B        Constrained at        0.5 Check
PLAT300_ALERT_4_G Atom Site Occupancy of H4B        Constrained at        0.5 Check
PLAT300_ALERT_4_G Atom Site Occupancy of H5         Constrained at        0.5 Check
PLAT300_ALERT_4_G Atom Site Occupancy of H5B        Constrained at        0.5 Check
PLAT300_ALERT_4_G Atom Site Occupancy of H8         Constrained at        0.5 Check
PLAT300_ALERT_4_G Atom Site Occupancy of H8B        Constrained at        0.5 Check
PLAT300_ALERT_4_G Atom Site Occupancy of H11        Constrained at        0.5 Check
PLAT300_ALERT_4_G Atom Site Occupancy of H11B       Constrained at        0.5 Check
PLAT300_ALERT_4_G Atom Site Occupancy of H13        Constrained at        0.5 Check
PLAT300_ALERT_4_G Atom Site Occupancy of H13B       Constrained at        0.5 Check
PLAT300_ALERT_4_G Atom Site Occupancy of H25        Constrained at        0.5 Check
PLAT300_ALERT_4_G Atom Site Occupancy of H25B       Constrained at        0.5 Check
PLAT300_ALERT_4_G Atom Site Occupancy of H27        Constrained at        0.5 Check
```

```
PLAT300_ALERT_4_G Atom Site Occupancy of H27B       Constrained at        0.5 Check
PLAT300_ALERT_4_G Atom Site Occupancy of H29A       Constrained at        0.5 Check
PLAT300_ALERT_4_G Atom Site Occupancy of H29B       Constrained at        0.5 Check
PLAT300_ALERT_4_G Atom Site Occupancy of H29C       Constrained at        0.5 Check
PLAT300_ALERT_4_G Atom Site Occupancy of H29D       Constrained at        0.5 Check
PLAT300_ALERT_4_G Atom Site Occupancy of H29E       Constrained at        0.5 Check
PLAT300_ALERT_4_G Atom Site Occupancy of H29F       Constrained at        0.5 Check
PLAT300_ALERT_4_G Atom Site Occupancy of H30A       Constrained at        0.5 Check
PLAT300_ALERT_4_G Atom Site Occupancy of H30B       Constrained at        0.5 Check
PLAT300_ALERT_4_G Atom Site Occupancy of H30C       Constrained at        0.5 Check
PLAT300_ALERT_4_G Atom Site Occupancy of H30D       Constrained at        0.5 Check
PLAT300_ALERT_4_G Atom Site Occupancy of H30E       Constrained at        0.5 Check
PLAT300_ALERT_4_G Atom Site Occupancy of H30F       Constrained at        0.5 Check
PLAT300_ALERT_4_G Atom Site Occupancy of H31A       Constrained at        0.5 Check
PLAT300_ALERT_4_G Atom Site Occupancy of H31B       Constrained at        0.5 Check
PLAT300_ALERT_4_G Atom Site Occupancy of H31C       Constrained at        0.5 Check
PLAT300_ALERT_4_G Atom Site Occupancy of H31D       Constrained at        0.5 Check
PLAT300_ALERT_4_G Atom Site Occupancy of H31E       Constrained at        0.5 Check
PLAT300_ALERT_4_G Atom Site Occupancy of H31F       Constrained at        0.5 Check
PLAT300_ALERT_4_G Atom Site Occupancy of H32A       Constrained at        0.5 Check
PLAT300_ALERT_4_G Atom Site Occupancy of H32B       Constrained at        0.5 Check
PLAT300_ALERT_4_G Atom Site Occupancy of H32C       Constrained at        0.5 Check
PLAT300_ALERT_4_G Atom Site Occupancy of H32D       Constrained at        0.5 Check
PLAT300_ALERT_4_G Atom Site Occupancy of H32E       Constrained at        0.5 Check
PLAT300_ALERT_4_G Atom Site Occupancy of H32F       Constrained at        0.5 Check
PLAT300_ALERT_4_G Atom Site Occupancy of H34A       Constrained at        0.5 Check
PLAT300_ALERT_4_G Atom Site Occupancy of H34B       Constrained at        0.5 Check
PLAT300_ALERT_4_G Atom Site Occupancy of H34C       Constrained at        0.5 Check
PLAT300_ALERT_4_G Atom Site Occupancy of H35A       Constrained at        0.5 Check
PLAT300_ALERT_4_G Atom Site Occupancy of H35B       Constrained at        0.5 Check
PLAT300_ALERT_4_G Atom Site Occupancy of H35C       Constrained at        0.5 Check
PLAT300_ALERT_4_G Atom Site Occupancy of H36A       Constrained at        0.5 Check
PLAT300_ALERT_4_G Atom Site Occupancy of H36B       Constrained at        0.5 Check
PLAT300_ALERT_4_G Atom Site Occupancy of H36C       Constrained at        0.5 Check
PLAT300_ALERT_4_G Atom Site Occupancy of C48        Constrained at        0.5 Check
PLAT300_ALERT_4_G Atom Site Occupancy of C48B       Constrained at        0.5 Check
PLAT300_ALERT_4_G Atom Site Occupancy of C49        Constrained at        0.5 Check
PLAT300_ALERT_4_G Atom Site Occupancy of C49B       Constrained at        0.5 Check
PLAT300_ALERT_4_G Atom Site Occupancy of C50        Constrained at        0.5 Check
PLAT300_ALERT_4_G Atom Site Occupancy of C50B       Constrained at        0.5 Check
PLAT300_ALERT_4_G Atom Site Occupancy of H48A       Constrained at        0.5 Check
PLAT300_ALERT_4_G Atom Site Occupancy of H48B       Constrained at        0.5 Check
PLAT300_ALERT_4_G Atom Site Occupancy of H48C       Constrained at        0.5 Check
PLAT300_ALERT_4_G Atom Site Occupancy of H48D       Constrained at        0.5 Check
PLAT300_ALERT_4_G Atom Site Occupancy of H50        Constrained at        0.5 Check
PLAT300_ALERT_4_G Atom Site Occupancy of H50B       Constrained at        0.5 Check
PLAT300_ALERT_4_G Atom Site Occupancy of Cl3B       Constrained at       0.15 Check
PLAT300_ALERT_4_G Atom Site Occupancy of Cl4B       Constrained at       0.15 Check
PLAT300_ALERT_4_G Atom Site Occupancy of C55B       Constrained at       0.15 Check
PLAT300_ALERT_4_G Atom Site Occupancy of H55C       Constrained at       0.15 Check
PLAT300_ALERT_4_G Atom Site Occupancy of H55D       Constrained at       0.15 Check
PLAT300_ALERT_4_G Atom Site Occupancy of Cl53       Constrained at       0.35 Check
PLAT300_ALERT_4_G Atom Site Occupancy of Cl54       Constrained at       0.35 Check
PLAT300_ALERT_4_G Atom Site Occupancy of C55        Constrained at       0.35 Check
PLAT300_ALERT_4_G Atom Site Occupancy of H55A       Constrained at       0.35 Check
PLAT300_ALERT_4_G Atom Site Occupancy of H55B       Constrained at       0.35 Check
PLAT301_ALERT_3_G Main Residue  Disorder .............(Resd  1  )         81% Note
```

```
PLAT302_ALERT_4_G Anion/Solvent/Minor-Residue Disorder (Resd  2  )        18% Note
PLAT302_ALERT_4_G Anion/Solvent/Minor-Residue Disorder (Resd  3  )       100% Note
PLAT302_ALERT_4_G Anion/Solvent/Minor-Residue Disorder (Resd  4  )       100% Note
PLAT304_ALERT_4_G Non-Integer Number of Atoms in ..... (Resd  3  )       0.75 Check
PLAT304_ALERT_4_G Non-Integer Number of Atoms in ..... (Resd  4  )       1.75 Check
PLAT410_ALERT_2_G Short Intra H...H Contact  H11      ..H16A     .       2.05 Ang.
                                                    x,y,z  =      1_555 Check
PLAT410_ALERT_2_G Short Intra H...H Contact  H13      ..H20B     .       2.14 Ang.
                                                    x,y,z  =      1_555 Check
PLAT410_ALERT_2_G Short Intra H...H Contact  H13B     ..H20B     .       2.01 Ang.
                                                    x,y,z  =      1_555 Check
PLAT410_ALERT_2_G Short Intra H...H Contact  H16A     ..H25      .       2.12 Ang.
                                               1-x,2-y,-z  =      3_675 Check
PLAT410_ALERT_2_G Short Intra H...H Contact  H16A     ..H25B     .       2.03 Ang.
                                               1-x,2-y,-z  =      3_675 Check
PLAT410_ALERT_2_G Short Intra H...H Contact  H20B     ..H27      .       2.11 Ang.
                                               1-x,2-y,-z  =      3_675 Check
PLAT410_ALERT_2_G Short Intra H...H Contact  H20B     ..H27B     .       2.07 Ang.
                                               1-x,2-y,-z  =      3_675 Check
PLAT410_ALERT_2_G Short Intra H...H Contact  H45B     ..H48D     .       2.12 Ang.
                                                    x,y,z  =      1_555 Check
PLAT410_ALERT_2_G Short Intra H...H Contact  H46A     ..H48C     .       1.96 Ang.
                                                    x,y,z  =      1_555 Check
PLAT411_ALERT_2_G Short Inter H...H Contact  H3B      ..H51B     .       2.08 Ang.
                                        -1/2+x,3/2-y,1/2+z  =      4_576 Check
PLAT414_ALERT_2_G Short Intra D-H..H-X       H1       ..H22      .       1.95 Ang.
                                                    x,y,z  =      1_555 Check
PLAT432_ALERT_2_G Short Inter X...Y Contact  C40      ..C50      .       3.20 Ang.
                                               1-x,1-y,-z  =      3_665 Check
PLAT779_ALERT_4_G Suspect or Irrelevant (Bond) Angle(s) in CIF ...       8.00 Deg.
              C26  -C15  -C26B    3_675   1_555   3_675 ......     #   93 Check
PLAT811_ALERT_5_G No ADDSYM Analysis: Too Many Excluded Atoms ....          ! Info
PLAT860_ALERT_3_G Number of Least-Squares Restraints ............         287 Note
PLAT912_ALERT_4_G Missing # of FCF Reflections Above STh/L=  0.600        237 Note
PLAT933_ALERT_2_G Number of HKL-OMIT Records in Embedded .res File          1 Note
                2  0  4,
PLAT941_ALERT_3_G Average HKL Measurement Multiplicity ..........         1.9 Low
PLAT978_ALERT_2_G Number C-C Bonds with Positive Residual Density.          1 Info
PLAT992_ALERT_5_G Repd & Actual _reflns_number_gt Values Differ by          4 Check
```

```
   0 ALERT level A = Most likely a serious problem - resolve or explain
   0 ALERT level B = A potentially serious problem, consider carefully
  11 ALERT level C = Check. Ensure it is not caused by an omission or oversight
 169 ALERT level G = General information/check it is not something unexpected

   0 ALERT type 1 CIF construction/syntax error, inconsistent or missing data
  20 ALERT type 2 Indicator that the structure model may be wrong or deficient
  18 ALERT type 3 Indicator that the structure quality may be low
 140 ALERT type 4 Improvement, methodology, query or suggestion
   2 ALERT type 5 Informative message, check
```

It is advisable to attempt to resolve as many as possible of the alerts in all categories. Often the minor alerts point to easily fixed oversights, errors and omissions in your CIF or refinement strategy, so attention to these fine details can be worthwhile. In order to resolve some of the more serious problems it may be necessary to carry out additional measurements or structure refinements. However, the purpose of your study may justify the reported deviations and the more serious of these should normally be commented upon in the discussion or experimental section of a paper or in the "special_details" fields of the CIF. checkCIF was carefully designed to identify outliers and unusual parameters, but every test has its limitations and alerts that are not important in a particular case may appear. Conversely, the absence of alerts does not guarantee there are no aspects of the results needing attention. It is up to the individual to critically assess their own results and, if necessary, seek expert advice.

Publication of your CIF in IUCr journals

A basic structural check has been run on your CIF. These basic checks will be run on all CIFs submitted for publication in IUCr journals (*Acta Crystallographica*, *Journal of Applied Crystallography*, *Journal of Synchrotron Radiation*); however, if you intend to submit to *Acta Crystallographica Section C* or *E* or *IUCrData*, you should make sure that full publication checks are run on the final version of your CIF prior to submission.

Publication of your CIF in other journals

Please refer to the *Notes for Authors* of the relevant journal for any special instructions relating to CIF submission.

PLATON version of 29/11/2023; check.def file version of 14/09/2023

Datablock Pseu - ellipsoid plot

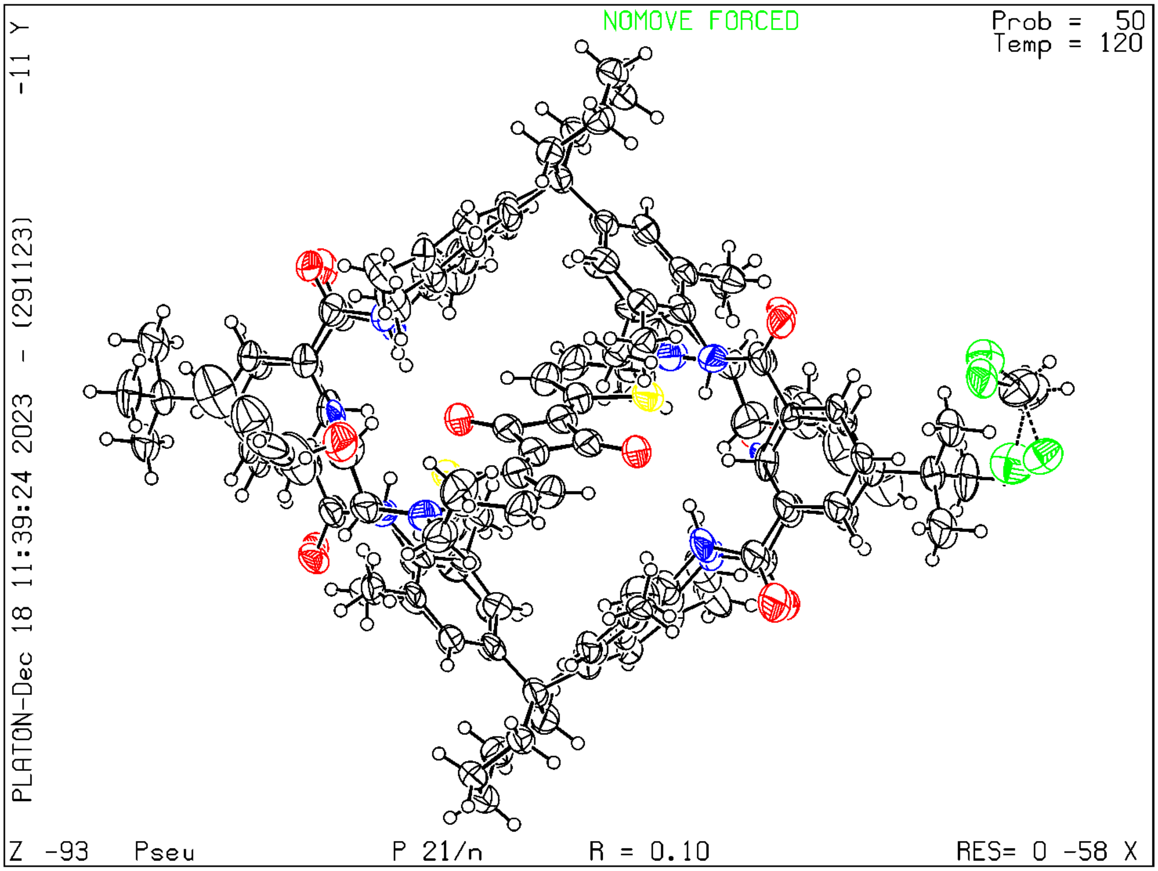

Supplement: Supplementary file 6 — Supplementary Data 4 [file 42004_2024_1312_MOESM6_ESM.pdf]
